# Bridging Place-Based Astrobiology Education with Genomics, Including Descriptions of Three Novel Bacterial Species Isolated from Mars Analog Sites of Cultural Relevance

**DOI:** 10.1089/ast.2023.0072

**Published:** 2023-12-20

**Authors:** Rebecca D. Prescott, Yvonne L. Chan, Eric J. Tong, Fiona Bunn, Chiyoko T. Onouye, Christy Handel, Chien-Chi Lo, Karen Davenport, Shannon Johnson, Mark Flynn, Jennifer A. Saito, Herb Lee, Kaleomanuiwa Wong, Brittany N. Lawson, Kayla Hiura, Kailey Sager, Mia Sadones, Ethan C. Hill, Derek Esibill, Charles S. Cockell, Rosa Santomartino, Patrick S.G. Chain, Alan W. Decho, Stuart P. Donachie

**Affiliations:** ^1^ Department of Biology, University of Mississippi, University, Mississippi, USA.; ^2^ School of Life Sciences, University of Hawai‘i at Mānoa, Honolulu, Hawai‘i, USA.; ^3^ National Aeronautics and Space Administration, Johnson Space Center, Houston, Texas, USA.; ^4^ Office of Community Science, ‘Iolani School, Honolulu, Hawai‘i, USA.; ^5^ UK Centre for Astrobiology, School of Physics and Astronomy, University of Edinburgh, United Kingdom.; ^6^ Los Alamos National Laboratory, Biosciences Division, Los Alamos, New Mexico, USA.; ^7^ Pacific American Foundation, Kailua, Hawai‘i, USA.; ^8^ Kauluakalana, Kailua, Hawai‘i, USA.; ^9^ Department of Environmental Health Sciences, Arnold School of Public Health, University of South Carolina, Columbia, South Carolina, USA.

**Keywords:** Place-based education, MinION, Lava caves, *Bradyrhizobium*, *Brenneria*, *Paraflavitalea.*

## Abstract

Democratizing genomic data science, including bioinformatics, can diversify the STEM workforce and may, in turn, bring new perspectives into the space sciences. In this respect, the development of education and research programs that bridge genome science with “place” and world-views specific to a given region are valuable for Indigenous students and educators. Through a multi-institutional collaboration, we developed an ongoing education program and model that includes Illumina and Oxford Nanopore sequencing, free bioinformatic platforms, and teacher training workshops to address our research and education goals through a place-based science education lens. High school students and researchers cultivated, sequenced, assembled, and annotated the genomes of 13 bacteria from Mars analog sites with cultural relevance, 10 of which were novel species. Students, teachers, and community members assisted with the discovery of new, potentially chemolithotrophic bacteria relevant to astrobiology. This joint education-research program also led to the discovery of species from Mars analog sites capable of producing *N*-acyl homoserine lactones, which are quorum-sensing molecules used in bacterial communication. Whole genome sequencing was completed in high school classrooms, and connected students to funded space research, increased research output, and provided culturally relevant, place-based science education, with participants naming three novel species described here. Students at St. Andrew's School (Honolulu, Hawai‘i) proposed the name *Bradyrhizobium prioritasuperba* for the type strain, BL16A^T^, of the new species (DSM 112479^T^ = NCTC 14602^T^). The nonprofit organization Kauluakalana proposed the name *Brenneria ulupoensis* for the type strain, K61^T^, of the new species (DSM 116657^T^ = LMG = 33184^T^), and Hawai‘i Baptist Academy students proposed the name *Paraflavitalea speifideiaquila* for the type strain, BL16E^T^, of the new species (DSM 112478^T^ = NCTC 14603^T^).

## 1. Introduction

Genomics research has emerged as a key tool in space biology and astrobiology. Recent space-related genomic studies include microbial studies aboard the International Space Station (ISS) (Castro-Wallace *et al.,* 2017; Haveman *et al.,* 2021) that are engaged in the search for extraterrestrial life (Bashir *et al.,* 2021; Payler *et al.,* 2019), and studies of the origins of life on Earth (Arriola *et al.,* 2020; Harris *et al.,* 2021; Maggiori *et al.,* 2021). These topics are of interest to a large portion of the public that wish to participate in the scientific process, and their participation could lead to further exploration and understanding of space and biology.

Genomics research has not commonly been taught in communities or secondary classrooms and has been slow to trickle into undergraduate programs. There also has been little effort to connect specifically with communities of Indigenous and minoritized peoples from genomic science educators. This lack of education and community involvement is partly due to the specialized equipment required for genomic work (*i.e.,* DNA sequencers, pipettes). Genomics also requires the use of bioinformatic tools, an interdisciplinary field that develops and applies computer algorithms to analyze genomic data, and communities and school teachers often have limited training in these techniques. These issues are compounded by a lack of access to computers and high-speed internet connections in many schools and communities, and may be worse in remote areas or in minoritized communities.

Within minoritized and Indigenous communities there can also be a lack of trust in genomics researchers due to prior negative experiences (James *et al.,* 2014; Guglielmi, 2019; Morgan *et al.,* 2019; McCartney *et al.,* 2022). For example, in some Indigenous communities, DNA samples that were collected for human genomics–related research for a specific, tribal-approved study were then also used for other research topics (*i.e.,* migration patterns, etc.) without the consent of the tribe or individuals, and without cultural awareness and sensitivity to those research questions (Guglielmi, 2019).

The development of easily portable DNA sequencing technologies, such as Oxford Nanopore's MinION, and the creation of user-friendly bioinformatics platforms with complete workflows (see EDGE Bioinformatics https://edgebioinformatics.org, Li *et al.,* 2017; and Kbase https://www.kbase.us, Arkin *et al.,* 2018) have provided an avenue to break down these barriers. They can help build trust and interest in genomics through community use and education (Cervantes *et al.,* 2021; De Vivo *et al.,* 2022) as well as more directly involve communities in ongoing research, building their local needs into research projects and research questions. Such programs are more likely to encourage students and the community to be actively engaged with the scientific community and will lead to a more diverse STEM workforce.

Additionally, genomics research and education projects that work with community to incorporate local, Indigenous, and cultural perspectives into their design can bridge genomics with “place” and provide new perspectives and knowledge. These Indigenous ways of knowing and doing are often less connected to our contemporary education and research systems. This can limit our exploration of science, which often excluded traditional ecological knowledge. We define TEK here as a system of knowledge, traditions, or practices that are heavily dependent on “place” and focused on relationships, including humans, as part of the system, rather than individual components of a system (Molnar and Babai, 2021). Our education model is defined here as *Place-Based Science Education* (PBSE), which combines modern scientific knowledge with TEK and place (Semken, 2005; Reyhner *et al.,* 2011) and can provide a platform for the inclusion of epistemologies and TEK within the scientific process and education (Davidson-Hunt and O'Flaherty, 2007; Bang and Medin, 2010; carr *et al.,* 2017; Rayne *et al.,* 2022).

Astronomy, astrobiology, environmental science, biology, and other disciplines have had success teaching topics through a PBSE lens (Barney-Nez *et al.,* 2016; Johnson and Elliott, 2020; Alexiades *et al.,* 2021). The fields of genomics and bioinformatics would benefit from similar efforts. For example, in this study, the genomes of bacteria isolated from sites of cultural significance were studied, with discussion of those sites in a cultural context included in education and research through PBSE practices. This can include discussion and recognition of origin stories, place history, cultural sensitivity, and the ethical collection and use of genomic data and samples in modern research.

Here, we present results from an ongoing joint research-education program that investigates the diversity and potential functional roles of bacteria from Mars analog sites, many with cultural relevance. Specific research goals included identification of *N*-acyl homoserine lactone signals (AHLs) from extreme environments. AHLs are chemical signaling molecules used by microorganisms to coordinate gene expression in response to environmental stimuli, in a process called quorum sensing (Papenfort and Bassler, 2016; Prescott and Decho, 2020). That process may enhance the ability to survive in extreme conditions, such as on the Moon and Mars. Such coordinated activities may also influence and expand the capabilities of some organisms to survive through chemolithotrophic metabolisms, with community members interacting to access nutrients and energy for growth.

Other research goals focused on identification of organisms associated with basalts and their ability to utilize minerals in the rock as an energy source through chemolithotrophic pathways and bioleaching. This includes the European Space Agency's BioRock project, which demonstrated biological extraction of elements from volcanic rocks (basalt) and asteroidal material in microgravity conditions on board the ISS, and in simulated martian gravity conditions for the purposes of future human space settlement (Cockell *et al.,* 2020, 2021; Santomartino *et al.,* 2020).

In the present study, we describe our three-pronged approach for building connections between these research programs, space agencies, and communities, with the use of experiential learning and a PBSE model in education. These include the following: (1) teacher and researcher training workshops in genomics and PBSE, (2) an educational program that utilizes the MinION (or other technologies for sequencing in the classroom) and bioinformatic platforms, and (3) a system to facilitate the long-term active participation and engagement among the community, students, and researchers, including cultural perspectives and place-based knowledge.

## 2. Methods

### 2.1. Teacher/Mentor training workshops

Teacher/Mentor training workshops were organized in 2018 and 2019 as one piece of our overall program, and are required to train educators and mentors in genomics and PBSE. Workshops focused on genomic data science in a microbial ecology, astrobiology, and sustainable resource management context and stressed both Western and Hawaiian worldviews, as well as “place” associated history and stories. Three-day teacher training workshops in 2018 were held at the University of Hawai‘i at Mānoa and ‘Iolani School. Four-day training workshops in 2019 were hosted by the Hawai‘i Institute of Marine Biology and the Pacific American Foundation at their 400-year-old Native Hawaiian aquaculture site, Waikalua Loko Iʻa. Participants included high school teachers, undergraduate students, postdoctoral researchers, and university staff and professors. Workshops comprised hands-on activities, classroom lessons, and presentations from various people involved in the program. The aims of the workshops were to teach (1) genomics and microbial ecology and the tools used in genomics research; (2) application of genomics research to the fields of space sciences, natural resources, and sustainability; and (3) principles and methods in PBSE (see Supplementary Information S1 for workshop agendas).

### 2.2. Educational program: ʻĀina-Informatics Network (AIN)

To administer and develop a long-term genomics and PBSE program that included secondary schools, researchers worked with the ʻĀina-Informatics Network (AIN) initiative based at ʻIolani School on the island of Oʻahu, Hawaiʻi (https://www.communityscience.iolani.org/ainainformatics). Researchers and teachers worked together to develop the program and bring genomic data science to local high school classrooms, which is still ongoing. The program's objective is to present and develop place-based curricula that encourages ethical practices in genomic science, centered on Hawaiʻi's unique relationships to the ‘āina (land), while generating original data through a modified citizen science approach. High school science teachers from both public and private schools across Hawaiʻi participate, as do their students, and partner researchers.

**FIG. 1.**
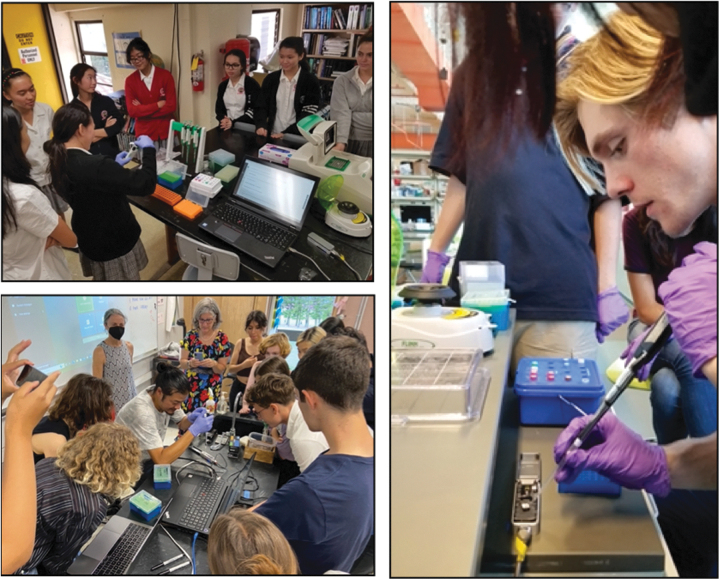
ʻĀina-Informatics Network (AIN) lab: genome sequencing in the classroom, Hawaiʻi. Top left: Students at St. Andrew's School sequencing BL16A^T^, a novel *Bradyrhizobium* bacteria isolated from Kaūmana cave, on the Island of Hawaiʻi. Bottom left: Eric ʻIwakeliʻi Tong with students and teachers sequencing. Right: Students at ʻIolani school load a MinION flow cell.

AIN developed a mobile sequencing lab capable of running DNA extraction, PCR, gel electrophoresis, and Oxford Nanopore MinION sequencing, and can complete whole genome sequencing in high school classrooms, as well as genome assembly and use of other bioinformatic tools in genomics (Fig. 1). Lessons and protocols have been developed that employ next generation sequencing technologies and use the same methods researchers employ. Overall, AIN addresses research questions related to food security, indigenous practices, space research, and local biodiversity, and it works directly with researchers on current, funded, research projects. The mobile lab equipment inventory (including reagents) is made available to AIN teachers and includes micropipettes, PCR thermocyclers, electrophoresis equipment, and centrifuges. In addition, the program provides the computer, sequencer, flow cells and library preparation kits required to run MinION sequencing in the classroom.

The bacterial cultures and DNA sequenced in AIN classrooms discussed here were obtained from the laboratory of Dr. Stuart Donachie, University of Hawaiʻi at Mānoa in the 2017–2019 school years. Details of the bacterial cultures, research methods, astrobiology, and cultural significance are described below. Currently, AIN has also established a long-term program to continue to assist with sequencing of cave microbes and encourage more direct engagement with community and students by allowing participants to name novel microbial species that are discovered (https://www.communityscience.iolani.org/ainainformatics/lavacave).

### 2.3. Sample collection

Lava caves from the 1922 lava flow in Kīlauea Caldera, Hawaiʻi, were sampled between 2006 and 2009 under permit #HAVO-2009-SCI-0029 to Stuart P. Donachie by the Hawaiʻi Volcanoes National Park, granted through a process that includes assessment and approval by a committee of Native Hawaiians (National Parks Service, Department of the Interior). Epilithic biofilms were collected by pushing uncapped sterile cryovials into the biofilm, without penetration of the underlying rock (Table 1). Subsamples were aseptically transferred in the field to liquid media for cultivation of heterotrophic and photoautotrophic bacteria. Subsamples were then subsequently transferred from the liquid media to solid media in Petri dishes, and colonies that arose were purified through repeated transfers on the same medium. Culture purity was checked by consistency of colony characteristics, Gram stains, and wet mounts. Pure cultures were maintained by transfer to fresh media at 2–12 week intervals, or they were stored in liquid medium containing 30% v/v glycerol at -80°C until retrieved for the work described here, beginning in 2017. Each culture's nearest validly published taxonomic neighbors were determined by BLAST and EzBioCloud comparisons of part of the culture's 16S rRNA gene nucleotide sequence (Altschul *et al.,* 1997; Yoon *et al.,* 2017).

**Table 1.** List of Cultured Microbes That Were Sequenced, Their Identification Based on 16S rRNA and Whole Genome Sequencing Using Digital DNA-DNA Hybridization Methods, the Nearest Known Species for Novel Strains, and the Location Strain Was Collected

Sample ID	Species ID	Nearest known species/strain	Site
BIC5C	*Paenibacillus* sp. nov.	*Paenibacillus taichungensis* DSM 19942^T^	Kīlauea caldera caves, Hawai‘i
BIC8F	*Cupriavidus* sp. nov.	*Cupriavidus necator* N-1	Kīlauea caldera caves, Hawai‘i
BIC9C	*Pseudomonas* sp. nov.	*Pseudomonas jessenii* DSM 17150^T^	Kīlauea caldera caves, Hawai‘i
BL16A^*^	*Bradyrhizobium prioritasuperba* sp. nov.	*Bradyrhizobium algeriense* RST89^T^	Kaūmana cave
BL16E^*^	*Paraflavitalea speifideiaquila* sp. nov.	*Paraflavitalea soli* KACC 17331^T^	Kaūmana cave
BL38	*Dermacoccus abyssi*	*Dermacoccus abyssi*	Pāhoa cave
C9-3	*Pseudomonas* sp. nov.	*Pseudomonas nitritireducens* WZBFD3-5A2^T^	Kīlauea caldera caves, Hawai‘i
JS2	*Fischerella* sp.	*Fischerella sesquitii* JSC-11	Kīlauea caldera caves, Hawai‘i
JS3	*Kovacikia* sp. nov.	*Crocosphaera subtropica* ATCC 51142^T^	Kīlauea caldera caves, Hawai‘i
K61^*^	*Brenneria ulupoensis* sp. nov.	*Brenneria rubrifaciens* ATCC 29291^T^	Ulupō lo‘i, Hawai‘i
SD	*Sphingomonas desiccabilis*	*Sphingomonas desiccabilis*	Colorado Plateau, USA
Y38-1Y	*Sphingomonas* sp. nov.	*Sphingomonas yantingensis* DSM 27244^T^	Salt Pond, San Salvador Island, Bahamas
Y88A	*Rhodococcus kroppenstedtii*	*Rhodococcus kroppenstedtii*	Salt Pond, San Salvador Island, Bahamas

^*^ Proposed name with descriptions of new species are presented here.

Heterotrophic bacteria were also cultivated from samples collected in 2017 from Pāhoa cave, a lava tube on the windward side of the island of Hawaiʻi, and Kaūmana Cave located near Hilo, Hawaiʻi (Table 1). The collection of samples was completed with a local guide from the Hawaiʻi Cave Conservancy with expertise and knowledge of kapu caves, which were not entered due to Hawaiian burials and artifacts. Pāhoa cave was created in flows 300–750 years old, with the cave itself thought to be older than 500 years. Kaūmana cave is much younger, ∼130 years old, and was created in flows from Mauna Loa in 1881.

Samples from Pāhoa and Kaūmana caves were collected by scraping a 1 cm^2^ area of cave wall with a sterile spatula into a sterile cryovial, without collection of, or damage to, the rock. Samples were stored in a cooler until their arrival the same day at the University of Hawaiʻi at Mānoa, where samples were immediately spread on Glucose Yeast Extract medium (GE; 10 g glucose, 10 g yeast extract, 14.2 g Difco agar per liter), R2A (Difco, Sparks, MD, USA), YM (10 g dextrose, 3 g yeast extract, 3 g malt extract, 5 g Bacto peptone, 14 g Difco agar per liter), YM plus 2.5% w/v sodium chloride, ZoBell's 2216E Marine Agar (MA; Difco), M1 (25 g sodium chloride, 3 g magnesium chloride, 1 g magnesium sulfate heptahydrate, 1 g calcium sulfate, 1 g potassium sulfate, 0.5 g calcium carbonate, 3.5 g Bacto peptone; adjust pH to 7.0–7.2 per liter; Watanabe, 1959), BG-11 for cyanobacteria (Rippka *et al.,* 1979), and Alga-Gro Freshwater Medium (Carolina Biological, Burlington, NC, USA). Colonies that arose were re-streaked for purification on the same media. Culture purity was checked by consistency of colony characteristics, Gram stains, and wet mounts, followed by whole genome sequencing attributes.

Two bacterial cultures, Y38-1Y and Y88A, were also isolated from a small piece of microbial mat collected from a hypersaline pond on San Salvador Island, Bahamas (originally Guanahaní Island; Table 1). Mats were desiccated and stored in a low-humidity environment by using desiccant beads at room temperature for 10 years in the laboratory of Dr. Alan Decho. A section of mat material, ∼2.5 cm^2^ in size, was rehydrated in 30 mL of 0.2 micron filter–sterilized seawater for 7 days in a sterile Petri dish. After incubation, live cells were observed in 10 μL of water that was collected from the hydrated mat or water in the Petri dish, under a 100 × oil immersion objective. 100 μL of the fluid from rehydrated mats or pieces of hydrated mat were spread on M1 media, MA +10% NaCl, BG11 + 10% w/v sodium chloride, and *Halobacterium* medium at pH 7.4 [per liter of distilled water: casamino acids (7.5 g), yeast extract (10 g), trisodium citrate (3 g), potassium chloride (2 g), magnesium sulfate heptahydrate (20 g), iron sulfate heptahydrate (0.05 g), manganese (II) sulfate monohydrate (0.20 mg), sodium chloride (250 g), and Bacto agar (20 g)]. In nature, these mats can be desiccated for months between rainfall events. Salinities of water overlying the mats range from 10 to 340 g/L (NaCl), and temperatures range seasonally between 37°C and 54°C (Preisner, 2018). The mats also host diverse and highly structured communities of archaea and bacteria (Preisner, 2018).

Other bacterial cultures included K61^T^, which was isolated from samples taken from a kalo wetland loʻi on the island of Oʻahu at Ulupō. At the time of collection in 2015, kalo (*Colocasia esculenta*) was planted in the loʻi. Finally, DNA from *Sphingomonas desiccabilis* CP1D^T^ was also sequenced. *Sphingomonas desiccabilis* was used in experiments on board the ISS for the BioRock experiment. This DNA was provided by Charles Cockell (University of Edinburgh). The organism was originally isolated from the soil crusts of the Colorado Plateau (Reddy and Garcia-Pichel, 2007) and is adapted to the extreme mineral and environmental conditions found within surface soil crusts of that region.

### 2.4. Astrobiology and cultural relevance of study sites/strains

Most of the bacterial strains studied here were cultivated from Hawaiian lava caves, Bahamas hypersaline mats, or a traditional Hawaiian agricultural site. Lava caves are an analog for potentially habitable places on the Moon and Mars, and may provide shelter for astronauts in upcoming missions. Recent data from the Moon suggest that some lava caves may be at a steady 17.2°C (Horvath *et al.,* 2022). Geologically, volcanoes and lava tubes in Hawaiʻi are the most similar to those on Mars. In addition, the discovery of a subsurface biosphere on Earth raised the possibility that life on Mars may have retreated to subsurface “oases” as climate conditions on the surface became unfavorable during the Hesperian era (Schulze-Makuch *et al.,* 2005), and therefore may be places for the discovery of extinct or extant life on Mars. The Bahamas hypersaline mats are also of relevance to Mars-related research because brines may exist today below the planet's surface (Gayen *et al.,* 2020; Nazari-Sharabian *et al.,* 2020). Native Hawaiian agriculture and crops, grown in basalt-type soils across the Hawaiian islands, are also relevant to future space missions and the development of sustainable agriculture on Mars and the Moon.

Many of these study sites are also of cultural significance, and this is important to include in research and lessons, through a PBSE model. This includes recognition and discussion of origin stories, place history, cultural sensitivity, and the ethical collection and use of genomic data and samples in modern research.

One site (Ulupō), where the bright pink culture K61^T^ was isolated, sits on the edge of what was the second largest Native Hawaiian fishpond, Kawainui, and houses the largest remaining Hawaiian Temple on Oʻahu. Ulupō Heiau, which measures 43 by 55 m, with walls to ∼9 m in height, is surrounded by natural springs that feed flooded plots (lo‘i) of kalo (*Colocasia esculenta*), a plant of significant cultural and agricultural importance. K61^T^ was isolated from one of these lo‘i. Kalo is a representation of the Hawaiian people's ancestry and a life giver. Also, Kawainui fishpond had mud at the bottom of the pond that was sought after for food. The mud, called lepoʻai (edible mud), was speckled pink and gelatinous (Keliikanakaole, 1872), suggesting that it was possibly a benthic microbial mat.

Lava caves are also places of historical and cultural significance in Hawaiʻi (Kennedy and Brady, 1997; Stone *et al.,* 2005). Water, filtered through porous lava rock, drips from the ceiling in many lava tubes and would have served as an important source of fresh, clean water for Native Hawaiians. Some lava tubes were used as burial sites, and many are *kapu* (forbidden) and protected today. Others likely were places of refuge during war or natural disasters, and some caves contain significant archeological remains (Stone *et al.,* 2005). In Native Hawaiian oral traditions, Pele, the Goddess of the volcanoes and of island building, was taught how to make fire in a lava cave by her uncle, Lonomakua, and tasked with keeping the fires of Kīlauea and Mauna Loa volcanoes active so that life above would be sustained through time (see TedTalk MauiX: https://www.youtube.com/watch?v=8PDipPnD2d8, 2012, for more details). These caves and their oral traditions represent volcanic eruptions that happen over and over through time in the Hawaiian Islands, and are where the land and life begins: they are a link to the geological history of the islands and to the People of the Hawaiian Islands.

### 2.5. DNA extraction and Sanger sequencing for initial strain identification

DNA was extracted from cultivated bacteria with the Qiagen PowerSoil Kit (Germantown, MD, USA) in the Donachie Lab. Initial sequencing of a fragment of the 16S rRNA gene in each microbe was then amplified in polymerase chain reactions (PCR) with primers 27F (5'-AGAGTTTGATCMTGGCTCAG-3') and 1492R (5'-GGYTACCTTGTTACGACTT-3'), GoTaq Green Master Mix (Promega, Madison, WI, USA), and 1 μL of template DNA. PCR conditions were an initial denaturation at 95°C for 5 min (1 cycle), followed by 30 cycles of 95°C (30 s), 55°C (30 s), 72°C (90 s), and a single final extension at 72°C for 7 min. PCR products were purified by using the Qiagen PCR purification kit and sequenced in an ABI 3730XL in the Advanced Studies in Genomics, Proteomics and Bioinformatics (ASGPB) core DNA sequencing facility at the University of Hawaiʻi at Mānoa, with primers 27F, 981R (5'-GGGTTGCGCTCGTTGCGGG-3), 530R (5'-GTATTACCGCGGCTGCTG-3'), 515F (5'-GTGCCAGCMGCCGCGGTAA-3'), and 1492R. Sequences were manually edited and assembled in SeqMan II v5.03 (DNASTAR, Madison, WI, USA) and compared to 16S rRNA gene sequences of those in type strains through BLAST searches at the NCBI, and in EZBioCloud. Cultures considered likely to represent novel species (based on a low identity match of the 16S rRNA gene sequence with that of their nearest neighbors in BLAST and EzBioCloud searches) were selected for whole genome sequencing and assembly in collaboration with AIN. Additionally, strains identified as AHL producers or of importance in other astrobiology research were also provided to the AIN program.

### 2.6. Whole genome sequencing: Illumina sequencing

Whole-genome libraries were prepared for each culture's DNA with the Nextera XT DNA Library Prep Kit (Illumina, San Diego, CA, USA). Library fragment size was determined on a Bioanalyzer High Sensitivity DNA chip (Agilent Technologies, Santa Clara, CA, USA). Libraries were quantified by using the Quant-iT PicoGreen dsDNA Assay Kit (Invitrogen, Carlsbad, CA, USA) and then normalized and pooled. Each library was sequenced on an Illumina MiSeq in the ASGPB to generate 301 bp paired-forward and reverse reads. We aimed for 50X coverage of each culture's genome. This was based on genome sizes of the culture's nearest neighbors, as well as anticipation of long-read data generated by MinION Nanopore sequencing that would increase the coverage.

### 2.7. ʻĀina-Informatics Network DNA extraction and MinION sequencing

AIN extracted DNA from cultures for MinION sequencing using the Machery-Nagel NucleoSpin DNA Stool Kit (Düren, Germany), a different DNA extraction protocol than what was used for Sanger or Illumina sequencing. This was to increase the yield of DNA, as MinION devices and protocols require large amounts of DNA for sequencing. All strains were sequenced by using the Rapid Barcoding Kit (SQK-RBK004), with flow cell type R9.4, except BL38, which was run on a Flongle (ONT, Oxford, UK). Run times varied, and some strains were multiplexed with up to three other strains, while others used the whole flow cell for one strain (see Supplementary Information S2 for specific Nanopore sequencing details for each culture).

### 2.8. Genome assembly and annotation

Sequencing data from both Illumina and MinION platforms were imported into the EDGE Bioinformatics platform (https://edgebioinformatics.org; Li *et al.,* 2017) and assembled with three assemblers: SPAdes, using Illumina data only (Bankevich *et al.,* 2012); LRASM, using the Miniasm algorithm with MinION Nanopore data only (Li, 2016); UniCycler, using Illumina and MinION data together (Wick *et al.,* 2017). One culture, JS3, was also assembled using LRASM-wtdbg2 (Ruan and Li, 2020) to improve its assembly. Students completed assemblies by using SPAdes and LRASM methods and were completed in EDGE Bioinformatics whole genome assembly pipelines. Researchers checked genome assemblies generated by AIN students and teachers, and adjusted as needed to improve assemblies. Since students were often able to generate very large amounts of MinION Nanopore data for each strain, those data were subsampled by using the tool seqtk (https://github.com/lh3/seqtk) at 10–50% of data, and assemblies were compared to determine the optimum subsample data size for Nanopore reads. Researchers completed assemblies with Unicycler in 2020, when Unicycler became available in EDGE. Unicycler was not available on EDGE at the time students were completing sessions in AIN.

For SPAdes assembly, Illumina reads were trimmed with FaQCs (Lo and Chain, 2014), and reads with a quality score of 30 or higher were retained and assembled. Reads of a minimum 50 bp were retained, a minimum 200 bp contig length was selected, and Burrows–Wheeler aligner-maximum exact matches (BWA-MEM; Li and Durbin, 2009) was used as a validation aligner. For MinION only data, using Miniasm or wtdbg2 assembly tools, Racon was used for error correction (Vaser *et al.,* 2017), and Minimap2 was used as validation aligner (Li, 2018). For UniCycler assembly, we applied normal bridging, using long reads with a minimum of 1000 bp and generating a minimum contig length of 2000 bp with BWA-MEM to validate the alignment. Using the .fastg files from SPAdes and LSRAM-Miniasm assembly tools in EDGE, assembly graphs were created with Bandage (Wick *et al.,* 2015). Various assemblies were then compared for completeness, accuracy, and contamination (*i.e.,* potentially more than one bacterial species in the sequencing data) using a number of statistics and graphics generated in this process. These metrics included the following: number of contigs, %GC content, assembly graphs, and percentage of reads mapped back to contigs. Assemblies that were considered the best were then run through CheckM (Parks *et al.,* 2015) on the Kbase platform to evaluate their completeness and contamination. CheckM uses a set of marker genes from genomes of closely related species, and looks for duplication of these genes to determine a percentage of contamination. It also estimates the genome's completeness by evaluating missing marker genes.

Annotation of genomes was completed in EDGE Bioinformatics using PROKKA (Seemann, 2014). This was followed by evaluation of genomes using FeGenie (Garber *et al.,* 2020) for iron metabolism, acquisition and storage associated genes, HMS-S-S, a tool for the identification of sulphur metabolism-related genes (Tanabe and Dahl, 2022), and an in-house hidden Markov model (HMM) for *N*-acyl homoserine lactone-based quorum sensing in bacteria.

Three of the strains in this study were not novel, and reference genomes were available in the NCBI database. Reference genome comparisons for the nearest neighbors of strains Y88A, BL38, and SD were completed in the EDGE Bioinformatics pipeline for mapping a genome to a reference genome. Since reference genomes in the database are not as contiguous as the genomes assembled in this study, our genome assemblies were used as the “reference genome” in EDGE, and NCBI reference genomes were input as contig files. This comparison allowed us to evaluate our assemblies against previously sequenced genomes.

### 2.9. Phylogenetic tree construction

For the three novel species described here, phylogenetic trees were constructed to help identify the strain, and its taxonomic placement in relation to its closest genetic neighbors using DSMZ phylogenomics pipeline adapted to single genes (Meier-Kolthoff *et al.,* 2014). Maximum-likelihood trees were constructed using the 16S rRNA gene sequences extracted from the genomes of BL16A^T^, BL16E^T^, and K61^T^, as well as closely related type strains. Each 16S rRNA gene sequence was aligned with other 16S rRNA sequences of related type strains: pairwise sequence similarities were calculated for 16S rRNA genes available through the GGDC web server (http://ggdc.dsmz.de; Meier-Kolthoff *et al.,* 2013, 2022). A multiple sequence alignment was created with MUSCLE, and ML and MP trees inferred from the alignment with RAxML and TNT, respectively (Edgar, 2004; Goloboff *et al.,* 2008; Stamatakis, 2014). Rapid bootstrapping in conjunction with the autoMRE bootstopping criterion and subsequent search for the best tree was used for maximum-likelihood (Pattengale *et al.,* 2010). One thousand bootstrapping replicates were used in conjunction with tree-bisection-and-reconnection branch swapping, and 10 random sequence addition replicates for maximum-parsimony. Sequences were checked for a compositional bias using the χ^2^ test as implemented in PAUP* (Swofford, 2002). The ML tree was inferred under the GTR+GAMMA model and rooted by midpoint-rooting (Hess and De Moraes Russo, 2007).

## 3. Results

### 3.1. Genome sequencing

Students and teachers in the AIN program, working with researchers at the University of Hawaiʻi at Mānoa, University of Edinburgh, and Los Alamos National Laboratory, sequenced and assembled 13 bacterial genomes (Supplementary Information S3 for all assembly graphs). All assembled genomes are publicly available and have been deposited at DDBJ/EMBL/GenBank under accession numbers SAMN37483209–SAMN37483221 within BioProject PRJNA1019399.

Unicycler produced a more complete genome assembly than assembly with MinION or Illumina data alone (see Supplementary Information S3 for comparative assembly graphs). Estimates of genome completeness ranged from 89.48% (JS3) to 99.85% (BIC5C), with 11 of the 13 strains having a greater than 99% genome completeness estimate (Table 2). Genome contamination, an estimated number of duplicate copies of universal single copy genes (SCGs) within an assembled genome, was very low for all strains, ranging from 0.0% for BL38 and BIC5C, to 1.7% for C9-3 (Table 2).

**Table 2.** Genome Assembly Metrics for the 13 Bacteria Genomes Sequenced by Students and Researchers

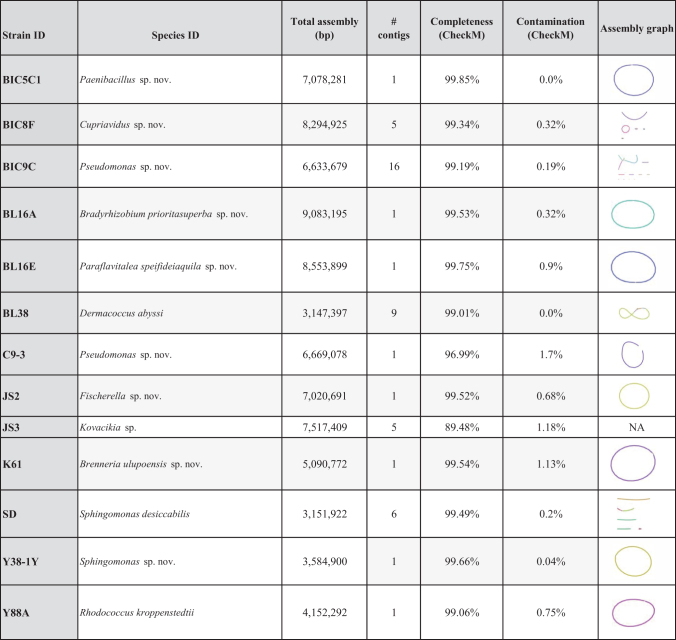

Total genome assembly sizes are given in number of base pairs (bp). Genome assembly completeness was assessed through CheckM. Assembly graphs of hybrid assemblies are provided to illustrate completeness and potential plasmids.

For MinION sequencing, most samples were multiplexed with up to three strains on a single MinION flow cell (see Supplementary Information S2), which at times did not generate enough data to produce a near-complete genome. However, students produced 12.96 Gb and 14 Gb of data for BL16A^T^ and SD, respectively (see Supplementary Information Table S4 for data generated), but on average generated 3.94 Gb of data per strain using the MinION.

### 3.2. Functional gene annotation

**FIG. 2.**
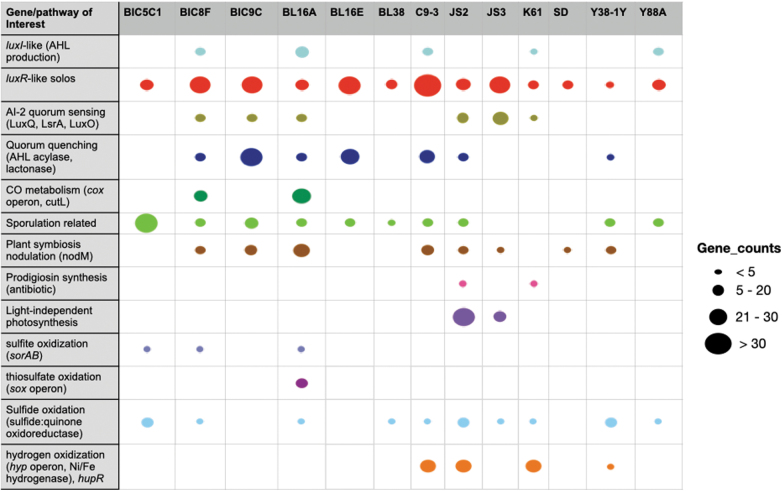
Genes and pathways of interest from whole genome sequencing and annotation of the 13 bacterial genomes described here. Annotations were completed using PROKKA within the EDGE Bioinformatics pipeline, as well as tools FeGenie, HMM-S-S, and an in-house Hidden Markov Model (HMM) for the detection of putative luxI and luxR genes involved in quorum sensing, a type of bacterial chemical signaling process that uses the molecule *N*-acyl homoserine lactone (AHLs). The strain ID is listed at the top of each column, with the various genes of interest in each row. Each pathway of interest is represented by a different color, and larger colored circles represent a higher gene count in that species. For example, luxR-like gene homologs, which make the receptor proteins for various AHL signaling molecules, and up or down regulating genes controlled by a particular AHL signaling molecule, ranged in gene count from 10 luxRs in Y38-1Y to 43 in C9-3.

Functional gene annotation identified thousands of coding regions for each strain, many of relevance to the various research projects underlying this study. At least five strains had the potential to express quorum sensing signals (*luxI* gene; Fig. 2), allowing further study of AHL production through mass spectrometry (data not shown). For example, a complex *N*-acyl-homoserine lactone type quorum sensing system was identified in the genome of BL16A^T^ through in-house HMM models developed for *Bacteria luxI*- and *luxR*-like genes. A cognate pair of *lux*-like genes were located in the genome, which may encode the production of 4-coumaroyl-homoserine lactone synthase (RpaI) and its receptor protein (RpaR). Two copies of the *rsaR* gene were identified by PROKKA annotation and in-house HMMs. A *lasI* gene that may produce *N*-3-oxododecanoyl homoserine lactone, an autoinducer molecule which binds to *lasR,* was also identified. A third *luxI* homolog was identified in the genome, along with 16 potential *luxR* homologs, suggesting this strain is capable of receiving multiple quorum sensing signals.

All strains had the ability to detect AHL molecules (*luxR* solos), and counts of *luxR* solos within each genome ranged from 10 (Y38-1Y) to 43 (C9-3; Fig. 2). At least seven strains had the ability to disrupt quorum sensing signals in a process called quorum quenching (AHL acylase, lactonase; Fig. 2), including three strains with *luxI*-like genes that encode production of AHL quorum sensing molecules. Annotation of the JS2 and JS3 genomes, both cyanobacteria, did not find AHL producing genes (*luxI*), but did find Autoinducer-2 (AI-2) sensor kinase/phosphatase genes (*luxQ*), a different type of quorum sensing system used by many bacteria (Waters and Bassler, 2005). Both JS2 and JS3 had putative *luxR-*like solo genes, giving them the potential to respond to AHL signals, although they may not make such signals. Four other strains, including three with AHL-type quorum sensing ability, also had AI-2 quorum sensing capabilities (Fig. 2).

Other chemolithotrophic pathways of interest that were detected included complete or near complete hydrogen oxidizing pathways in four strains (*hyp* operon, *hupR,* Ni/Fe hydrogenase, quinone-reactive Ni/Fe hydrogenase; Fig. 2). Hydrogen redox pathways are of interest in resource recovery and pollutant removal, including CO_2_ capture (Lin *et al.,* 2022). Potential sulfur metabolisms were also examined (see Fig. 2 and Supplementary Information S5 for full results of sulfur-related pathways). The gene Sulfide:quinone reductase (SQR), whose product catalyzes sulfide oxidation, was found. This enzyme is common in phototrophic or lithotrophic bacteria (Ghosh and Dam, 2009) and may contribute to sulfur oxidation chemolithotrophy. Three of the cave strains (BL16A^T^, BIC5C1, and BIC8F), and one strain from the salt ponds (Y38-1Y), possessed genes for sulfite dehydrogenases (*sorAB*), suggesting the potential for sulfur oxidizing chemolithotrophy (Fig. 2 and Supplementary Information S5; Tanabe and Dahl, 2022). BL16A^T^ also had a complete *sox* operon cluster (s*ox*XAYZBCD), which governs the Kelly–Friedrich pathway for thiosulfate oxidation, central to facultative sulfur-oxidizing metabolisms of alphaproteobacteria (Friedrich *et al.,* 2001). BIC8F had an incomplete sox cluster and does not have the soxX gene, a monoheme subunit of the heterodimeric periplasmic c-type cytochrome (*soxXA*). This microbe is from the Betaproteobacteria, which have a less well-characterized mechanism of sulfur oxidation. Both BL16A^T^ and BIC8F have the *aprM* gene, which completes the sulfur oxidation pathway with a reversible APS reductase activity.

Other genes of interest included plant root nodulation-symbiosis genes (*nodM* and related genes) and sporulation related genes. BIC5C1 had 49 spore related genes, including endospore-genes (Fig. 2). Two strains (BL16A^T^, BIC8F) had a complex carbon monoxide metabolism pathway (*cox* operon, *cutL* genes), suggesting they are able to use carbon monoxide as a carbon source in chemolithoautotrophy (Fig. 2).

**FIG. 3.**
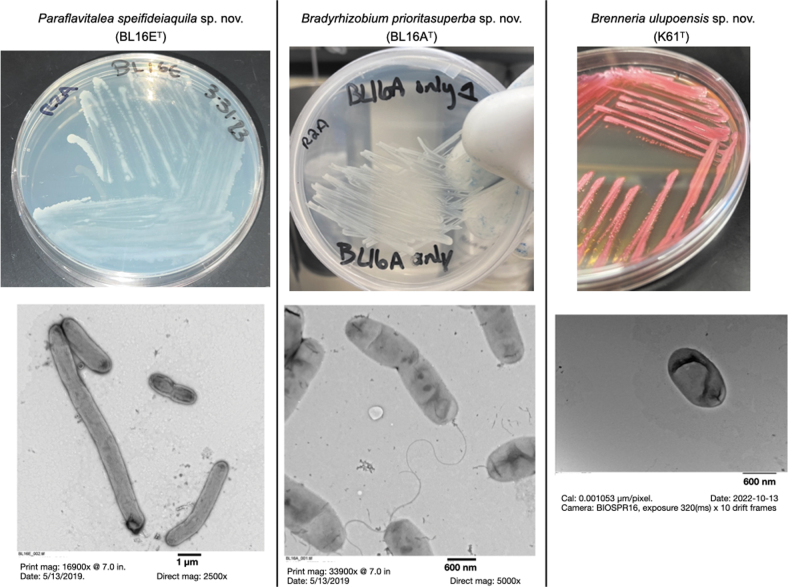
Images of three novel species described here, along with their transmission electron microscopy (TEM) images: BL16E^T^, BL16A^T^, and K61^T^. **Left**, top and bottom panels: BL16E^T^ with TEM image (direct magnification 2500 × ). **Center**, top and bottom panels: BL16A^T^ with TEM image. **Right**, top and bottom panels: K61^T^ with TEM image (scale bar = 600 nm). TEM images: cells were negatively stained with uranyl acetate and viewed on a 120 kV Hitachi HT7700 transmission electron microscope in the Biological Electron Microscope Facility at the University of Hawai‘i at Mānoa.

The cyanobacteria JS2 and JS3 have light-independent photosynthesis pathways (ferredoxin:protochlorophyllide reductase and related pathways), which are considered evolutionarily older than light-dependent photosynthesis (Fig. 2). These pathways are not thought to function in an oxygenated atmosphere. JS2 also had a gene associated with the production of the pink pigment and antibiotic, prodigiosin (*pigC* gene; Islan *et al.,* 2022). K61^T^ also produces prodigiosin and has prodigiosin synthesis-related genes. K61^T^ produces bright pink colonies on several media (Fig. 3).

**FIG. 4.**
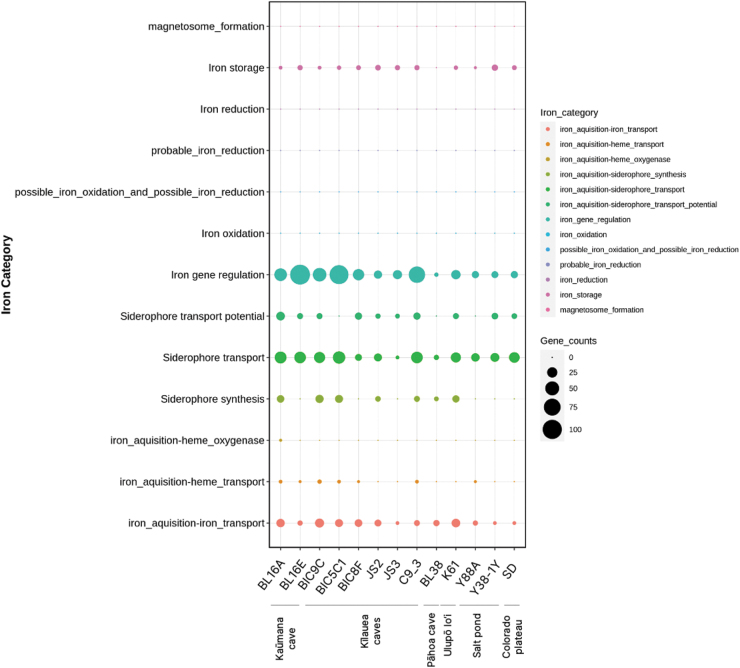
Dot-plot from FeGenie bioinformatics tool, which helps identify iron-related pathways and genes in the genomes of the 13 bacteria discussed here. Bacterial strain ID is given on the *x-*axis and is grouped by the location they were cultivated from. Iron-related gene categories are on the *y-*axis and represented by different colors in the key on the right of the dot-plot. Larger circles represent a higher count of genes in a given category, with a size key on the right side of the plot.

A number of strains had potential chemolithotrophic pathways and bioremediation-related genes, both of interest in space research. Six of the nine strains isolated from caves have biosynthesis pathways for the production of siderophores (Fig. 4), as did K61^T^ from Ulupō lo‘i. Siderophores are small molecular iron chelators secreted by microbes, which support iron acquisition in combination with active uptake mechanisms. While the remaining strains lacked their own siderophore biosynthesis pathways, all strains had the capability for siderophore transport systems, which suggests that all strains could commensally benefit from siderophore production by neighboring species or cells. No iron reduction or oxidation pathways were identified. These strains could use novel, non-homologous iron redox metabolisms or alternative electron donors in such pathways, or they are simply not iron redox-type chemolithotrophs.

### 3.3. Strain novelty

All but three of the strains discussed here represent novel species. Analysis of the 16S rRNA gene and genomes of Y88A and BL38 suggest that these strains belong to *Rhodococcus kroppenstedtii* and *Dermacoccus abyssi,* respectively. Strains were identified through digital DNA:DNA hybridizations (dDDH) of each genome assembly with genomes in the Type (Strain) Genome Server (TYGS; Meier-Kolthoff and Göker, 2019). Pairwise comparison of the BL38 genome assembly with type strain genomes in TYGS concluded our assembly was an 80.1% match to *D. abyssi* MT1.1^T^, the type strain of the species originally isolated from a sample collected at ∼11,000 m depth in the Mariana Trench's Challenger Deep (Abdel-Mageed *et al.,* 2020). Comparison with the reference genome of *D. abyssi* MT1.1^T^ (RefSeq:GCF_003515945.1) found 38 (69.09%) of 55 contigs in the reference genome mapped to our assembly of BL38, with >85% identity. Our assembly of BL38 has four contigs scaffolded together, with a total assembly size 3,147,397 bp (Table 2 and Supplementary Information S3). Average identity of contigs that mapped to our assembly was 96.59%. Our assembly included 168 gaps, 49,514 single nucleotide polymorphisms (SNPs), and 1,699 insertion–deletion mutations (indels). An orthoANIu comparison of the BL38 hybrid assembly with the genome of *Dermacoccus abyssi* MT1.1^T^ through EzBioCloud gave an OrthoANIu value of 97.59%: a value of >95% indicates the organisms from which the respective genomes were derived likely belong to the same species (Yoon *et al.,* 2017).

Pairwise comparison by dDDH of Y88A with type strains in TYGS concluded our assembly of Y88A was a 76.9% match to *Rhodococcus kroppenstedtii* DSM 44908^T^. A comparison of the reference genome for *R. kroppenstedtii* DSM 44908^T^ (RefSeq:GCF_900111805.1) revealed 28 of 30 contigs in the reference genome mapped to our assembly of Y88A, with >85% identity. Our assembly comprises one contig of 4,152,292 bp, and checkM estimated the assembly to be 99.06% complete (Table 2). The average identity of contigs that mapped to our assembly was 93.85%. There were 167 gaps, 82,125 SNPs, and 3,027 indels in the comparison to the reference genome. An orthoANIu comparison of the hybrid Y88A assembly with the genome of *R. kroppenstedtii* DSM 44908^T^ through EzBioCloud gave an orthoANIu value of 97.28%, evidence that the genomes of these organisms likely belong to the same species (Yoon *et al.,* 2017). A BLAST comparison of 1379 nt of the Y88A 16S rRNA gene showed the nearest type strain neighbors are *R. kroppenstedtii* DSM 44908^T^ (99.42%, 1364/1372 nt), and *Rhodococcus corynebacteroides* DSM 20151^T^ (99.20%, 1371/1382 nt).

Culture SD, provided by the Cockell lab, is a strain of *Sphingomonas desiccabilis* used in experiments aboard the ISS (Santomartino *et al.,* 2020). The taxonomic affiliation was confirmed through the TYGS database, which reported our assembled SD genome was a 100% match to the genome of *S. desiccabilis* CP1D^T^ in pairwise comparisons. The SD genome assembly was compared to the genome of *S. desiccabilis* strain CP1D^T^ reference genomes (NZ_SDPT01000004_1, NZ_SDPT01000001_1, NZ_SDPT01000002_1, NZ_SDPT01000003_1, NZ_SDPT01000005_1) using EDGE Bioinformatics. At least two of the three contigs from SD matched to *S. desiccabilis* strain CP1D^T^ reference genomes, with no gaps, indels, or SNPs. Node 1 and Node 2 of the *S. desiccabilis* strain CP1D^T^ reference genome had some gaps, but all five contigs matched to the SD assembled genome.

Ten strains were found to represent potentially novel species (Table 1). Three of the novel strains (BL16A^T^, BL16E^T^, and K61^T^) are described below, with additional descriptive data in Supplementary Information S6. These strains are proposed as type strains of new species; a name for each of these was proposed by students or community members associated with this study (Fig. 3). Descriptions and proposals of names for additional novel species will be formally presented elsewhere.

## 4. Novel Species Descriptions

### *4.1. Description of* Bradyrhizobium prioritasuperba *sp. nov.*

*Bradyrhizobium prioritasuperba* (pri.o.ri.ta.su.per'ba, N.L. nom. *prior* superior; adj. *superbus* proud, from “Priory Pride,” the nickname of St. Andrew's Schools, Honolulu, Hawai‘i, where the strain's genome was first sequenced).

Cells of BL16A^T^ stain Gram negative and are straight rods of 0.6–0.7 × 1.6–2.9 μm, motile by a single polar or subpolar flagellum. Colonies are off-white to beige, opaque, 1–2 mm in diameter, convex, smooth, shiny, and entire, and develop overnight at 30°C on Potato Dextrose Agar and R2A Agar. The strain is oxidase positive, catalase negative, and grows aerobically, microaerophilically, and anaerobically. BL16A^T^ grows on R2A Agar between 12°C and 35°C, but not on R2A or LB at 37°C. The pH range for growth in R2A Broth is 4–10. BL16A^T^ is viable in 30 mL sterile MilliQ (18 MΩ) water with 0.3 g of powdered basalt rock under anaerobic conditions (no shaking) for at least 6 months, with growth of BL16A^T^ on R2A media from those cultures after 6 months. Major fatty acids in whole cells are one or both of *cis*-11-octadecenoic acid and *cis*-12-octadecenoic acid, and octadecanoic acid.

The type strain, BL16A^T^ ( = DSM 112479^T^ = NCTC 14602^T^), was isolated from an epilithic biofilm in Kaūmana Cave on the island of Hawai‘i, USA. The DNA G + C content is 63.05%. The GenBank accession number for the BL16A^T^ 16S rRNA gene nucleotide sequence is MZ486441. The genome sequence of BL16A^T^ has been deposited at DDBJ/EMBL/GenBank (accession number SAMN37483212) under BioProject PRJNA1019399.

**FIG. 5.**
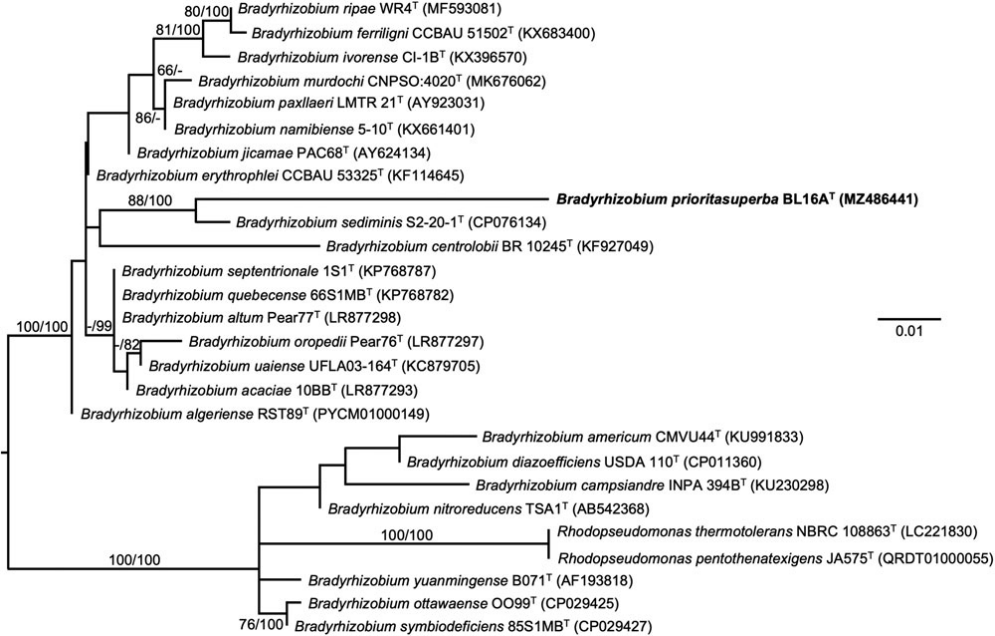
Maximum-likelihood tree based on 16S rRNA gene sequences extracted from the genomes of BL16A^T^ and related type strains, inferred under the GTR+GAMMA model and rooted by midpoint-rooting. Branches are scaled in terms of the expected number of substitutions per site. Scale bar represents 0.01 nucleotide substitutions per site. Numbers above branches are support values for the maximum likelihood tree (left) and maximum parsimony tree (right). Species and strain are followed in parentheses by the GenBank accession number of the 16S rRNA sequence for that strain. The ML bootstrapping did not converge, so 1000 replicates were conducted: average support was 49.83%.

Based on 1479 nucleotides in the 16S rRNA gene, the nearest type strain neighbor of BL16A^T^ in the EZBioCloud 16S database is *Bradyrhizobium algeriense* RST89^T^ (1274/1301, 97.92%). The nearest type strain neighbor of BL16A^T^ in BLAST searches at the NCBI is *B. valentinum* LmjM3^T^ (1443/1479, 97.57%). Such levels of nucleotide identity between the 16S rRNA genes in BL16A^T^ and its nearest neighbors support the establishment of a new species (Stackebrandt and Goebel, 1994). A maximum-likelihood tree based on 16S rRNA gene sequences extracted from the genomes of BL16A^T^ and related type strains on TYGS shows BL16A^T^ close to *B. sediminis* S2-20-1^T^ (Fig. 5). This strain is also the nearest neighbor through pairwise comparisons of genomes in dDDH (Fig. 5).

The Unicycler assembly of the BL16A^T^ genome using Illumina and MinION data comprised a single contig, with 99.4% of the sequencing reads mapping back to the genome assembly. The assembled genome was uploaded to TYGS for a whole genome-based taxonomic analysis by comparison through the MASH algorithm with the genomes of all published type strains in the TYGS database (Ondov *et al.,* 2016; Meier-Kolthoff and Göker, 2019); the analysis concluded that BL16A^T^ is a potential new species. The annotated genome comprises 8386 coding regions, and shows that, like many *Bradyrhizobium* spp., BL16A^T^ is capable of symbiotic interactions with plant roots through nodulation. Genes *nodD2* and *nodM* were found in the genome, but nif genes for nitrogen fixation were not annotated; NodD2 (nodulation protein 2) regulates the expression of the *nodABCFE* genes which encode other nodulation proteins in many *Bradyrhizobium* spp. (Ormeno-Orrillo and Martinez-Romero, 2019). However, PROKKA annotation did not locate *nodABCFE*. *NodD* is also a negative regulator of its own expression and binds flavonoids as inducers. *NodM* produces an isomer of glutamine-fructose-6-phosphate aminotransferase, which is involved in the production of the root hair deformation (HAD) factor in the nodulation process in *Rhizobium meliloti.* One *fixJ* gene was identified, a transcriptional regulator that induces the expression of both *nifA,* required for activation of classical *nif* and *fixJ*-genes, and *fixK,* required for *fixN* activation. This suggests that *nif* genes are present in this strain's genome, although they were not identified in the annotation.

BL16A^T^ shares a number of phenotypic and chemotaxonomic characteristics with extant *Bradyrhizobium* spp., such as cell shape, pigmentation, and temperature range for growth (see Supplementary Information S6, Table 1 within). The predominant fatty acids in BL16A^T^ are also consistent with those in related *Bradyrhizobium* spp., but with a smaller proportion of hexadecanoic acid, and relatively larger proportions of *cis*-9,10-methylenehexadecanoate and octadecanoic acid in BL16A^T^ (Supplementary Information S6, Table 2 within). However, these type strains can be distinguished from each other by their growth or absence of growth in the presence of 1% (w/v) sodium chloride in the culture medium, and the G + C content of their genomes (Supplementary Information S6 Table 1). We propose that BL16A^T^ is the type strain of a novel species in the genus *Bradyrhizobium,* for which the name *Bradyrhizobium prioritasuperba* sp. nov. is proposed.

### *4.2. Description of* Paraflavitalea speifideiaquila *sp. nov.*

*Paraflavitalea speifideiaquila* (spei.fi.dei.a.qui.la N.L. fem. *spes* hope; L. gen. *fides* faith; L. nom. *aquila* eagle, from “eagle of faith and hope,” selected by students of the Hawai‘i Baptist Academy, Honolulu, Hawai‘i, who sequenced the strain's genome).

Cells stain Gram negative and are non-motile, straight to slightly curved rods of 0.8–0.9 × 1.5–10 μm. Colonies are off-white to beige, opaque, ∼1–2 mm in diameter, convex, smooth, mucoid, and entire, and develop overnight on R2A at 30°C. This strain is catalase negative and oxidase positive. The strain is aerobic and not halotolerant. The temperature range for growth on R2A agar is 11–37°C. The pH range for growth in R2A Broth is 5–8. Predominant fatty acids are 13-methyl tetradecanoic acid and 3-hydroxy, 15-methyl hexadecanoic acid.

The type strain, BL16E^T^ ( = DSM 112478^T^ = NCTC 14603^T^), was isolated from an epilithic biofilm in Kaūmana Cave on the island of Hawai‘i, USA. The DNA G + C content of the strain's genome is 45.81%. The GenBank accession number for the BL16E^T^ 16S rRNA gene nucleotide sequence is MH894271. The strain's genome sequence has been deposited at DDBJ/EMBL/GenBank (accession number SAMN37483213) in BioProject PRJNA1019399.

**FIG. 6.**
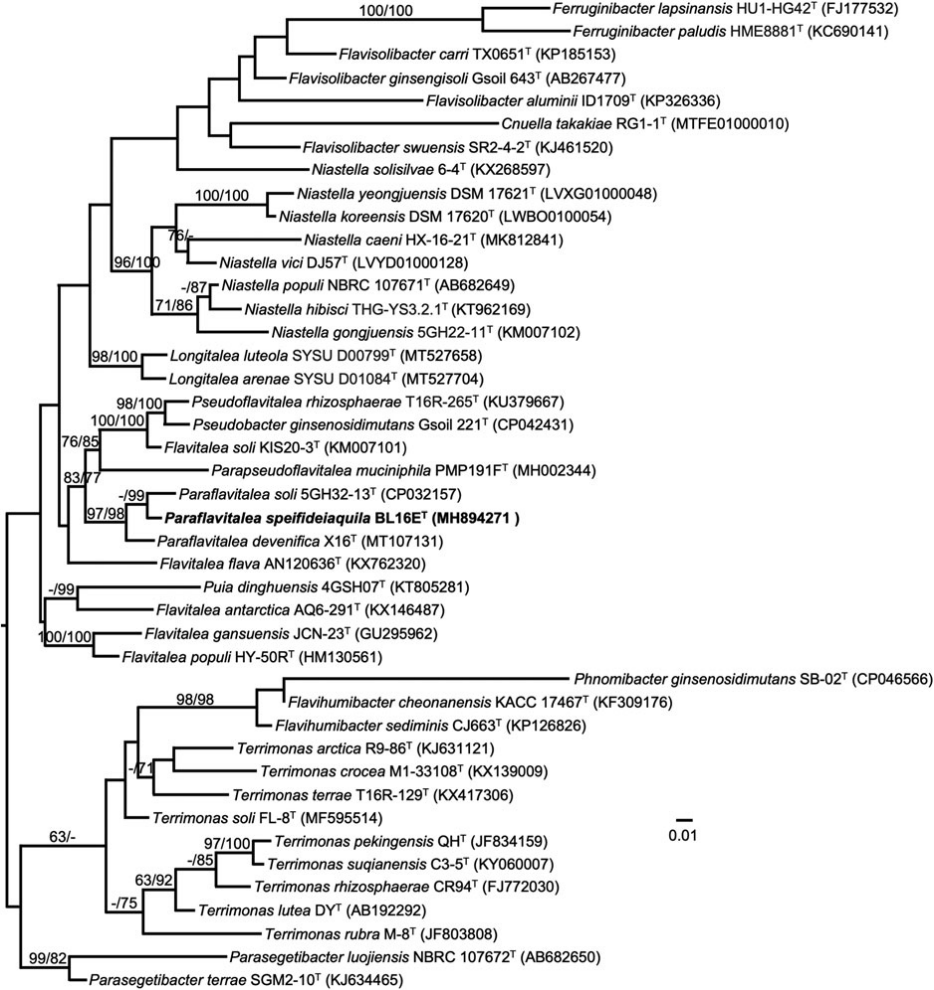
Maximum-likelihood tree based on 16S rRNA gene sequences extracted from the genomes of BL16E^T^ and related type strains, inferred under the GTR+GAMMA model and rooted by midpoint-rooting. Branches are scaled in terms of the expected number of substitutions per site. Scale bar represents 0.01 nucleotide substitutions per site. Numbers above branches are support values for the maximum-likelihood tree (left) and maximum parsimony tree (right). Species and strain are followed in parentheses by the GenBank accession number of the 16S rRNA sequence for that strain. The maximum likelihood bootstrapping converged after 900 replicates; average support was 56.70%.

Based on 1418 nucleotides in the 16S rRNA gene, the nearest type strain neighbor of BL16E^T^ in the EZBioCloud 16S database is *Paraflavitalea soli* 5GH32-12^T^ (1388/1408, 98.58%). The nearest type strain neighbor of BL16E^T^ in BLAST searches at the NCBI is *Flavitalea flava* AN120636^T^ (1352/1420, 95.21%). Such levels of nucleotide identity between the 16S rRNA genes in BL16E^T^ and its nearest neighbors support the establishment of a new species (Stackebrandt and Goebel, 1994). A maximum likelihood tree based on 16S rRNA gene sequences extracted from the genomes of BL16E^T^ and related type strains on TYGS shows BL16E^T^ in a distinct cluster with *Paraflavitalea soli* KACC 17331^T^ and *Paraflavitalea devenifica* X16^T^ (Fig. 6); these strains are also the two nearest neighbors through pairwise comparisons of the strains' genomes in dDDH (Fig. 6). The assembled genome was uploaded to TYGS for a whole genome-based taxonomic analysis by comparison through the MASH algorithm with the genomes of all published type strains in the TYGS database (Ondov *et al.,* 2016; Meier-Kolthoff and Göker, 2019); the analysis concluded that BL16E^T^ is a potential new species.

The annotated genome of BL16E^T^ assembled here comprises one chromosome of 8,553,899 bp and 7,031 coding regions. The genome assembly contains genes for pathways associated with utilization of C4-dicarboxylates under anaerobic conditions (*dcuB*), and anaerobic magnesium-protoporphyrin IX monomethyl ester cyclase (*bchE*), a key enzyme in bacteriochlorophyll biosynthesis pathways that is oxygen independent (Boldareva-Nuianzina *et al.,* 2013). BL16E^T^ also has anaerobic nitric oxide reductase transcription regulators (*norR*). This suggests that BL16E^T^ has some ability to function in an anaerobic environment. There are also several metal resistance, metal transport, or metal-associated pathways, including *czcA* and *czcB* genes for Cobalt-zinc-cadmium resistance, which, in *Cupriavidus metallidurans*, can efficiently remove Cd^+^ and Zn^+^ from microbial culture media (Diels *et al.,* 1995). Other metal-associated genes found include nickel-cobalt resistance (*cnrB*), cobalt-magnesium transport, copper export, and Fe^2+^ transport. Additionally, there are FeS cluster assembly proteins that repair oxygen-labile iron-sulfur clusters under oxidative stress and may facilitate iron uptake from extracellular iron chelators. CRISPR-Cas genes were also annotated: type III-associated RAMP protein (Csm3), and CRISPR-associated endonuclease Cas1 and Cas2.

BL16E^T^ shares phenotypic and chemotaxonomic characteristics with extant *Paraflavitalea* spp., such as cell shape, pigmentation, and temperature range for growth (Supplementary Appendix A Table 3). The predominant fatty acids in BL16E^T^ are consistent with those in type strains of species in closely related genera, such as *Pseudoflavitalea* and *Flavitalea* (Supplementary Information S6 Table 4), but the relatively high concentrations in BL16E^T^ and extant *Paraflavitalea* of 13-methyl tetradecanoic acid and 3-hydroxy, 15-methyl hexadecanoic acid, and their comparatively low concentrations of g-pentadecenoic acid and *cis*-9- or *cis*-10-hexadecenoic acids, distinguish them from members of the *Pseudoflavitalea* and *Flavitalea*. Based on the phenotypic, chemotaxonomic and genotypic data presented, we propose that BL16E^T^ is the type strain of a novel species in the genus *Paraflavitalea,* for which the name *Paraflavitalea speifideiaquila* sp. nov. is proposed.

### *4.3. Description of* Brenneria ulupoensis *sp. nov.*

*Brenneria ulupoensis* (u.lu.po.en'sis, N.L. fem. adj. *ulupoensis* originating from Ulupō, the area from which the type strain was isolated, proposed by Kaleomanuiwa Wong, Executive Director of the nonprofit Kauluakalana, which aims to restore and steward Ulupō heiau and Kawainui fishpond).

Cells of K61^T^ stain Gram negative and are rods of 0.7–0.8 × 1.5–2.2 μm. Colonies are opaque white, and pink to maroon, ∼1–2 mm in diameter, convex, smooth, shiny, and entire, and develop overnight on Glucose-Yeast Extract Agar at 30°C. It grows in Glucose Yeast Extract Broth containing 0 to 1% (w/v) NaCl, and on Glucose Yeast Extract Agar, and Potato Dextrose Agar between 10°C and 43°C, but not at 9°C or 44°C. K61^T^ grows optimally in the presence of 0–1% w/v sodium chloride but weakly in concentrations of 2–5% w/v. The strain is facultatively anaerobic, and catalase and oxidase positive. Predominant fatty acids are hexadecanoic acid (C16:0), *cis*-9-hexadecenoic acid (C16:1 ω7c) or *cis*-10-hexadecenoic acid (C16:1 ω6c), and *cis*-9,10-methylenehexadecanoic acid (C17:0 cyclo; see Supplementary Information S6 Table 5).

The type strain, K61^T^ (LMG = 33184^T^ = DSM 116657^T^), was isolated from turbid water in a kalo (*Colocasia esculenta*) lo‘i on O‘ahu, Hawai‘i, USA. The DNA G + C content of strain K61^T^ is 49.27%. The GenBank accession number for the K61^T^ 16S rRNA gene nucleotide sequence is MH894269. The strain's genome sequence has been deposited at DDBJ/EMBL/GenBank (accession number SAMN37483218) under BioProject PRJNA1019399.

**FIG. 7.**
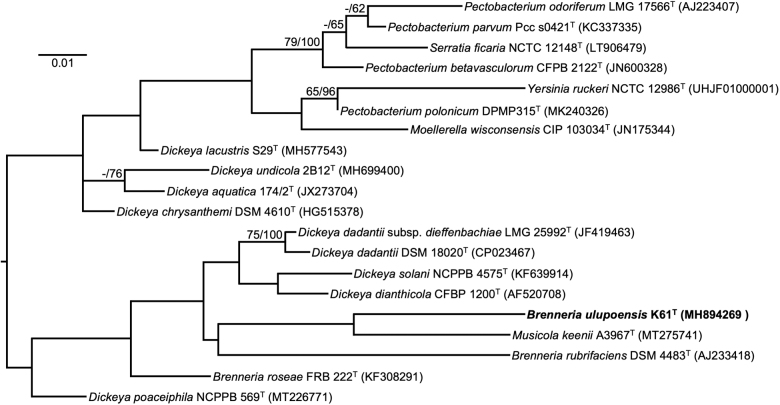
Maximum-likelihood tree based on 16S rRNA gene sequences extracted from the genomes of K61^T^ and related type strains, inferred under the GTR+GAMMA model and rooted by midpoint-rooting. Branches are scaled in terms of the expected number of substitutions per site. Scale bar represents 0.01 nucleotide substitutions per site. Numbers above branches are support values for the maximum-likelihood tree (left) and maximum parsimony tree (right). Species and strain are followed in parentheses by the GenBank accession number of the 16S rRNA sequence for that strain. Maximum-likelihood bootstrapping did not converge, so 1000 replicates were conducted; average support was 42.18%.

Based on 1529 nucleotides in the 16S rRNA gene, the nearest type strain neighbors of K61^T^ in the EZBioCloud 16S database are *Dickeya dadantii* subsp. *dadantii* NCPPB 898^T^ (1412/1462, 96.58%) and *Dickeya dadantii* subsp. *dieffenbachiae* LMG 25992^T^ (1412/1462, 96.6%). The nearest type strain neighbor of K61^T^ in BLAST searches at the NCBI is *Musicola keenii* A3967^T^ (1481/1531, 96.7%). Such levels of nucleotide identity between the 16S rRNA gene in K61^T^ and its nearest neighbors support the establishment of a new species (Stackebrandt and Goebel, 1994). A maximum likelihood phylogenetic tree based on 16S rRNA gene sequences extracted from the genomes of K61^T^ and related type strains on TYGS showed K61^T^ as closest to *Musicola keenii* A3967^T^ and *Brenneria rubrifaciens* ATCC 29291^T^ (Fig. 7). However, *M. keenii* A3967^T^ placed 18th among 19 strains based on percentage dDDH determined in TYGS (Fig. 7). The genus *Musicola* was recently established through reclassification of *Dickeya paradisiaca* as *Musicola paradisiaca* comb. nov., and the description of *Musicola keenii* sp. nov. (Hugouvieux-Cotte-Pattat *et al.,* 2021).

The K61^T^ assembled genome of 5,052,060 bp was uploaded to TYGS for a whole genome-based taxonomic analysis by comparison through the MASH algorithm with the genomes of all published type strains in the TYGS database (Ondov *et al.,* 2016; Meier-Kolthoff and Göker, 2019); the analysis concluded that K61^T^ is a potential new species.

The annotated, assembled genome shows the strain has both aerobic and anaerobic pathways and the ability to utilize various sulfoxide and N-oxide compounds under anaerobic conditions (*dmsBC* genes). There is also an anaerobic sulfite reductase operon (*asrABC*) and the *tusABCDE* sulfur transport operon in the genome. K61^T^ has genes involved in the nitrogen cycle, for example, assimilatory nitrate reductase (*Has* operon), nitrate respiration (*Nar* genes), and nitric oxide reductase genes (*Nor* genes). These pathways are common in plant and soil microbes. No motile cells were observed in liquid media, nor were flagella seen on cells observed by scanning electron microscopy. However, 29 genes involved in flagella formation (*Flh, Flg, Fli* operons) were detected.

K61^T^ shares phenotypic, chemotaxonomic, and genomic characteristics with *Muscicola* spp., *Brenneria* spp., and *Dickeya* spp., such as pigmentation, cell shape, and temperature range for growth. The relative abundances of dodecanoic acid (C12:0), *cis*-9,10-methylenehexadecanoic acid (C17:0 cyclo), and *cis*-10-11-methyleneoctadecanoic acid (C19:0 cyclo ω9c) distinguish these genera and the species within (Supplementary Appendix A Table 5). K61^T^ may also be distinguished by the presence of a C12:0 aldehyde. Further, K61^T^ and *Brenneria* spp. lack *cis*-9-heptadecenoic acid (C17:1ω8c), which is present in some *Dickeya* spp. (Tian *et al.,* 2016). Species in these genera and the *Muscicola* have genomes in the ∼4–5 Mbp range, but that of K61^T^ is the only one whose GC content is <50% (Supplementary Information S6 Table 6). Based on phenotypic, chemotaxonomic, and genotypic data, we propose that K61^T^ is the type strain of a novel species in the genus *Brenneria,* for which the name *Brenneria ulupoensis* sp. nov. is proposed.

## 5. Discussion

Through this ongoing joint-research education program we have sequenced and annotated the genomes of 13 bacteria from environments that are analogs of sites on Mars, many with cultural relevance. The 13 bacteria include putative novel species, and many have potential metabolic functions important in understanding (1) interspecies interactions in extreme environments (*i.e.,* quorum sensing) that may expand any one species' ability to survive extreme conditions, and (2) the ability to survive in basalt terrains, utilizing nutrients from the rock. This research has led to further study of new species that are capable of quorum sensing, as well as a new species of *Bradyrhizobium* (BL16A^T^) that is likely a chemolithotroph. These species are being used in further analyses and experiments in funded space research. Their discovery has greatly advanced our understanding of community interactions in these analog sites, an important aspect of microbial ecology that is still largely not understood in extreme environments.

This study also illustrates that current sequencing technologies (in particular the portable MinION), along with free bioinformatic platforms, can provide a link between research institutions and K-12 education programs with the goal of community-generated data that is a part of funded research projects from around the world. Students and teachers generated data and actively participated in research for a NASA Exobiology grant examining quorum sensing in extreme environments. They also assisted with preliminary research related to bioremediation and sustainable agriculture on Earth and beyond, and contributed to a European Space Agency funded project investigating biomining aboard the ISS. Data generated by AIN was comparable in quality to that generated by researchers working on these grants. These data have also added to microbial genomic databases and knowledge of bacterial species in Mars-related environments. In addition, sequencing and exploration of these strains has led to further studies of how microbes utilize basalts and meteorites as a source of energy, with potential future space biotechnology applications and the examination of novel strains under Mars-like conditions.

The program provided students with a participatory experience in ongoing funded research, creating a sense of belonging to a larger science community. Students and community members sequenced 13 bacterial genomes, mostly of new species from Hawaiian lava caves, and built on our understanding that biodiversity in Hawaiian caves is remarkable (Prescott *et al.,* 2022). Other publications have suggested that diversity is high in lava caves; this has relevance to exploration of the Moon and Mars where lava caves are common and may become human habitats (Fairén *et al.,* 2005; Léveillé and Datta, 2010). Such environments may have harbored life in the past and could also be vulnerable to forward contamination from Earth (Sauro *et al.,* 2020), which is relevant from a planetary protection perspective. Although this may be unavoidable, understanding how to study, preserve, and sustainably utilize these extraterrestrial environments will be key for human space exploration.

Working together, we also developed place-based and culturally responsible lessons related to the places these microbes were cultivated from and, when possible, connections to astrobiology. When such connections between knowledge, place, and local culture are included in education and research programs, the relationships (*pilina*) become the focus of understanding, and a more systems-based approach to science and education that is more diverse in ideas becomes possible. Combining Western-based science with Indigenous knowledge creates a more complex and holistic understanding of a given system, with a focus on the interactions within the system, including interactions with people. This can lead to a better understanding of key components of Earth's complex system and prevent some of the limitations that may be created by reductionist approaches. These methods can also help provide a connection between research institutions and diverse communities with the local environment.

### 5.1. Improvements going forward

Although the program overall has been very successful, improvements can be implemented to better bridge astrobiology and PBSE through genomics. First, ʻIolani is a private school and has a private funding source for their AIN program. ʻIolani has addressed this issue by creating a mobile lab that supplies everything needed for the genomic lessons, and has sought public and private funding to equip schools in their network. However, sequencing is expensive, and outreach programs like AIN require substantial and continued funding to serve their community. Thus, more permanent funding for public and private schools is needed for similar programs. Secondly, joint programs of this nature require continued engagement by scientists and community members, with regular communication and commitment from all parties, as research conclusions can take years to develop. Research findings must be shared with the participating schools and community members, and new research questions and funding should be sought with community participation, particularly in an effort to include TEK. Platforms for dissemination of research results to this audience must be provided and developed and would ideally include individual research projects by K-12 students, undergraduates, and graduate students to facilitate continued contact.

Bioinformatic platforms used in this program provided free, fully developed pipelines for the assembly and annotation of bacterial genomes, along with some additional analyses. However, because these are public resources, running the assemblies and annotations can take several days on public servers, unless the school has the computing resources to host local versions of these servers. AIN has now switched to using a local version of EDGE Bioinformatics to accommodate the shorter time periods needed for students to produce results in the classroom. AIN has also developed an end-to-end whole genome assembly and annotation pipeline for bacterial genomes using only long reads generated from the Oxford MinION platform, called the “Unicellular Long-read Assembler aNd Annotator” or “Ulana”. This pipeline was created as a student friendly introduction to a CLI/terminal based genome assembly and annotation. The source code for the pipeline is at GitHub (https://github.com/ehill-iolani/ulana) and has been created as a Docker image for convenient implementation via Docker containers (https://hub.docker.com/repository/docker/ethill/ulana/general).

Additionally, Oxford Nanopore's tablet-style devices for MinION sequencing and analysis (MinION Mk1C) could help improve access to computing power to complete analyses. However, these devices are currently expensive and limited to use of Oxford Nanopore's bioinformatic workflows, and may not always be suitable for certain analyses. Working to develop devices or joint systems for community use with private companies like Oxford Nanopore Technologies, as well as improving funding from state and federal agencies for such programs, particularly in rural areas, could help overcome these limitations.

## 6. Conclusions

Genomic data science is a growing field that can be applied to a wide range of studies beyond biology, and is interwoven with understanding our origins on Earth and possibly beyond. Environmental science, geology, astrobiology, sustainability science, and human-health-related fields, are all examples in which genomics plays a vital role in understanding ecological and evolutionary processes on Earth, and they may help us design more sustainable systems for Earth and beyond. They are also areas where discussion and inclusion of “place” and TEK can build stronger community connections for all who participate in such studies or programs. As we move further into space, with the development of commercial space stations, the field of genomics will be critical for understanding the effects of the off-world environment on biological systems. This includes food production in space, the impacts on to the human body of spaceflight, and the potential for mining operations on other planetary bodies and in the asteroid belt. Having a connection to our ancestors and life on Earth, supported through genomic studies, as well as “place”, will also become more critical for our well-being as we venture further into space.

Genomic data science is a key component in the conservation of biological systems on Earth, the development of sustainable systems for Earth's future, and even understanding our own bodies' responses to medicines, foods, and allergens. It is, therefore, vital that students, educators, and community members from various education levels understand studies that are based on DNA and RNA, and how these methods can be applied to solve current issues facing our global community. Such information will only become a greater part of our societies. It is also important that researchers and research institutions directly involve their local communities in their research whenever possible, to build scientifically-minded societies and better address problems that those communities are facing in a changing climate. The joint education and research program presented here is a step toward achieving these goals, and the contribution that students and community members have made is not minor. Assessment of the genomes of the organisms in this study is key to understanding how these organisms may respond in Mars or early Earth-like environments, the role of quorum sensing organisms in space-related research, and the functional roles microbes will provide in off-world communities. As a research group, including the students, we are also expanding our understanding of the diversity of microbes in Hawai‘i, one of the most biologically diverse places on Earth.

Fundamentally, content (knowledge), context (place), and the bio-cultural systems that develop in societies over generations a are inextricable. Future plans for the development of human bases on other planetary boundaries must consider this a and include the wide-ranging world-views represented in humanity. Not only will this preserve the diversity of cultures, but that diversity of perspectives and ways of *knowing* will be necessary to problem-solve and adapt to the changing climate on Earth, as well as for human space exploration of the Moon and Mars. When we lose either cultural or biological diversity, we lose a rich reservoir of ideas, potential tools, and bio-cultural systems that may be critical for society as it faces new challenges.

## Supplementary Material

Supplemental data

Supplemental data

Supplemental data

Supplemental data

Supplemental data

Supplemental data

## Abbreviations Used

AHLs: *N*-acyl homoserine lactone signals
AIN: ʻĀina-Informatics Network
ASGPB: Advanced Studies in Genomics, Proteomics and Bioinformatics
BWA-MEM: Burrows–Wheeler aligner-maximum exact matches
dDDH: digital DNA:DNA hybridizations
HMM: hidden Markov model
indels: insertion–deletion mutations
ISS: International Space Station
PBSE: place-based science education
SNPs: single nucleotide polymorphisms
TEK: traditional ecological knowledge
TYGS: Type (Strain) Genome Server

## Notes

Associate Editor: Daniella Scalice

## References

[B1] Abdel-Mageed WM, Juhasz B, Lehri B, *et al.* Whole genome sequence of *Dermacoccus abyssi* MT1.1 isolated from the Challenger Deep of the Mariana Trench reveals phenazine biosynthesis locus and environmental adaptation factors. Mar Drugs 2020;18(3):131; doi: 10.3390/md18030131. 32106586 PMC7143476

[B2] Alexiades AV, Haeffner MA, Reano D, *et al.* Traditional ecological knowledge and inclusive pedagogy increase retention and success outcomes of STEM students. Bull Ecol Soc Am 2021;102(4):e01924; doi: 10.1002/bes2.1924.

[B3] Altschul SF, Madden TL, Schäffer AA, *et al.* Gapped BLAST and PSI-BLAST: A new generation of protein database search programs. Nucleic Acids Res 1997;25:3389–3402; doi: 10.1093/nar/25.17.3389. 9254694 PMC146917

[B4] Arkin AP, Cottingham RW, Henry CS*, et al*. KBase: The United States Department of Energy Systems Biology Knowledgebase. Nat Biotechnol 2018;36:566–569; doi: 10.1038/nbt.4163. 29979655 PMC6870991

[B5] Arriola LA, Cooper A, Weyrich LS. Palaeomicrobiology: Application of ancient DNA sequencing to better understand bacterial genome evolution and adaptation. Front Ecol Evol 2020;8:40; doi: 10.3389/fevo.2020.00040.

[B6] Bang M, Medin D. Cultural processes in science education: Supporting the navigation of multiple epistemologies. Science Ed 2010;94(6):1008–1026; doi: 10.1002/sce.20392.

[B7] Bankevich A, Nurk S, Antipov D, *et al.* SPAdes: A new genome assembly algorithm and its applications to single-cell sequencing. J Comput Biol 2012;19:455–477; doi: 10.1089/cmb.2012.0021. 22506599 PMC3342519

[B8] Barney-Nez A, Carron A, Scalice, D. NASA and the Navajo Nation. A presentation to the NASA AI/AN Working Group; 2016.

[B9] Bashir AK, Wink L, Duller S, *et al.* Taxonomic and functional analyses of intact microbial communities thriving in extreme, astrobiology-relevant, anoxic sites. Microbiome 2021;9:50. 33602336 10.1186/s40168-020-00989-5 PMC7893877

[B10] Boldareva-Nuianzina EN, Blahova Z, Sobotka R, *et al.* Distribution and origin of oxygen-dependent and oxygen-independent forms of Mg-protoporphyrin monomethylester cyclase among phototrophic proteobacteria. Appl Environ Microbiol 2013;79(8):2596–2604; doi: 10.1128/AEM.00104-13. 23396335 PMC3623192

[B11] Castro-Wallace SL, Chiu CY, John KK, *et al.* Nanopore DNA sequencing and genome assembly on the international space station. Sci Rep 2017;7:18022. 29269933 10.1038/s41598-017-18364-0 PMC5740133

[B12] carr t, Kenefic LS, Ranco DJ. Wabanaki Youth in Science (WaYS): A tribal mentoring and educational program integrating traditional ecological knowledge and Western science. Journal of Forestry 2017;115(5):480–483; doi: 10.5849/jof.16-066.

[B13] Cervantes J, Perry C, Wang MC. Teaching next-generation sequencing to medical students with a portable sequencing device. Perspect Med Educ 2021;10(4):252–255; doi: 10.1007/s40037-020-00568-2. 32125679 PMC8368599

[B14] Cockell CS, Santomartino R, Finster K*, et al*. Space station biomining experiment demonstrates rare earth element extraction in microgravity and Mars gravity. Nat Commun 2020;11(1):5523; doi: 10.1038/s41467-020-19276-w. 33173035 PMC7656455

[B15] Cockell CS, Santomartino R, Finster K, *et al.* Microbially-enhanced vanadium mining and bioremediation under micro- and Mars gravity on the international space station. Front Microbiol 2021;12:641387. 33868198 10.3389/fmicb.2021.641387 PMC8047202

[B16] Davidson-Hunt IJ, O'Flaherty MR. Researchers, Indigenous peoples, and place-based learning communities. Society and Natural Resources 2007;20:291–305; doi: 10.1080/08941920601161312.

[B17] De Vivo M, Lee HH, Huang YS, *et al.* Utilisation of Oxford Nanopore sequencing to generate six complete gastropod mitochondrial genomes as part of a biodiversity curriculum. Sci Rep 2022;12(1):9973; doi: 10.1038/s41598-022-14121-0. 35705661 PMC9200733

[B18] Diels L, Dong Q, van der Lelie D, *et al.* The czc operon of *Alcaligenes eutrophus* CH34: From resistance mechanism to the removal of heavy metals. J Ind Microbiol 1995;14(2):142–153; doi: 10.1007/BF0156989. 7766206

[B19] Edgar RC. MUSCLE: Multiple sequence alignment with high accuracy and high throughput. Nucleic Acids Res 2004;32(5):1792–1797; doi:10.1093/nar/gkh340. 15034147 PMC390337

[B20] Fairén AG, Dohm JM, Uceda ER, *et al.* Prime candidate sites for astrobiological exploration through the hydrogeological history of Mars. Planet Space Sci 2005;53(13):1355–1375; doi: 10.1016/j.pss.2005.06.007.

[B21] Friedrich CG, Rother D, Bardischewsky F, *et al.* Oxidation of reduced inorganic sulfur compounds by bacteria: emergence of a common mechanism? Appl Environ Microbiol 2001;67:2873–2882. 11425697 10.1128/AEM.67.7.2873-2882.2001 PMC92956

[B22] Garber AI, Nealson KH, Okamoto A, *et al.* FeGenie: A comprehensive tool for the identification of iron genes and iron gene neighborhoods in genome and metagenome assemblies. Front Microbiol 2020;11:37; doi: 10.3389/fmicb.2020.00037. 32082281 PMC7005843

[B23] Gayen P, Sankarasubramanian S, Ramani VK. Fuel and oxygen harvesting from martian regolithic brine. Proc Natl Acad Sci USA 2020;117(50):31685–31689; doi: 10.1073/pnas.2008613117. 33257545 PMC7749281

[B24] Ghosh W, Dam B. Biochemistry and molecular biology of lithotrophic sulfur oxidation by taxonomically and ecologically diverse bacteria and archaea. FEMS Microbiol Biol 2009;33(6):999–1043; doi: 10.1111/j.1574-6976.2009.00187.x. 19645821

[B25] Goloboff PA, Farris JS, Nixon KC. TNT, a free program for phylogenetic analysis. Cladistics 2008;24:774–786; doi:10.1111/j.1096-0031.2008.00217.x.

[B26] Guglielmi G. Facing up to genome injustice. Nature Briefings 2019;568:290–293. 10.1038/d41586-019-01166-x 30992587

[B27] Harris F, Dobbs J, Atkins D, *et al.* Soil fertility interactions with *Sinorhizobium*-legume symbiosis in a simulated martian regolith; effects on nitrogen content and plant health. PLoS One 2021;16(9):e0257053; doi: 10.1371/journal.pone.0257053. 34587163 PMC8480890

[B28] Haveman NJ, Khodadad CLM, Dixit AR, *et al.* Evaluating the lettuce metatranscriptome with MinION sequencing for future spaceflight food production applications. NPJ Microgravity 2021;7:22. 34140518 10.1038/s41526-021-00151-x PMC8211661

[B29] Hess PN, De Moraes Russo CA. An empirical test of the midpoint rooting method. Biol J Linn Soc 2007;92:669–674; doi:10.1111/j.1095-8312.2007.00864.x. PMC7110036 32287391

[B30] Horvath T, Hayne PO, Paige DA. Thermal and illumination environments of lunar pits and caves: Models and observations from the Diviner Lunar Radiometer experiment. Geophys Res Lett 2022;49(14):e2022GL099710; doi: 10.1029/2022GL099710.

[B31] Hugouvieux-Cotte-Pattat N, Jacot des-Combes CJ, Briolay J, *et al.* Proposal for the creation of a new genus *Musicola* gen. nov., reclassification of *Dickeya paradisiaca* (Samson et al. 2005) as *Musicola paradisiaca* comb. nov. and description of a new species *Musicola keenii* sp. nov. Int J Syst Evol Microbiol 2021;71(10):5037; doi: 10.1099/ijsem.0.005037. 34617878

[B32] Islan GA, Rodenak-Kladniew B, Noacco N, *et al.* Prodigiosin: A promising biomolecule with many potential biomedical applications. Bioengineered 2022;13(6):14227–14258; doi: 10.1080/21655979.2022.2084498. 35734783 PMC9342244

[B33] James R, Tsosie R, Sahota P*, et al*. Exploring pathways to trust: A tribal perspective on data sharing. Genet Med 2014;16(11):820–826; doi: 10.1038/gim.2014.47. 24830328 PMC4224626

[B34] Johnson A, Elliott S. Culturally relevant pedagogy: A model to guide cultural transformation in STEM departments. J Microbiol Biol Educ 2020;21(1):1–12; doi: 10.1128/jmbe.v21i1.2097. PMC7195162 32431767

[B35] Keliikanakaole JB. “Lepo Ai Ia.” Nupepa 1872. Available from https://nupepa-hawaii.com/tag/lepo-ai-ia [Last accessed 11/7/2023]. (Originally published Ka Nupepa Kuokoa, Buke XI, Helu 43, Aoao 2. October 26, 1872.)

[B36] Kennedy J, Brady JE. Into the netherworld of island Earth: A reevaluation of refuge caves in ancient Hawaiian society. Geoarchaeology 1997;12(6):641–655; doi: 10.1002/(SICI)1520-6548(199709)12:6<641::AID-GEA6>3.0.CO;2-Z.

[B37] Léveillé RJ, Datta S. Lava tubes and basaltic caves as astrobiological targets on Earth and Mars: A review. Planet Space Sci 2010;58(4):592–598; doi: 10.1016/j.pss.2009.06.004.

[B38] Li H. Minimap and miniasm: Fast mapping and de novo assembly for noisy long sequences. Bioinformatics 2016;32(14):2103–2110; doi: 10.1093/bioinformatics/btw152. 27153593 PMC4937194

[B39] Li H. Minimap2: Pairwise alignment for nucleotide sequences. Bioinformatics 2018;34(18):3094–3100; doi: 10.1093/bioinformatics/bty191. 29750242 PMC6137996

[B40] Li H, Durbin R. Fast and accurate short read alignment with Burrows-Wheeler transform. Bioinformatics 2009;25(14):1754–1760; doi: 10.1093/bioinformatics/btp324. 19451168 PMC2705234

[B41] Li PE, Lo CC, Anderson JJ, *et al.* Enabling the democratization of the genomics revolution with a fully integrated web-based bioinformatics platform. Nucleic Acids Res 2017;45(1):67–80; doi: 10.1093/nar/gkw1027. 27899609 PMC5224473

[B42] Lin L, Huang H, Zhang X, *et al.* Hydrogen-oxidizing bacteria and their applications in resource recovery and pollutant removal. Sci Total Envir 2022;835:155559; doi: 10.1016/j.scitotenv.2022.155559. 35483467

[B43] Lo CC, Chain P. Rapid evaluation and quality control of next generation sequencing data with FaQCs. BMC Bioinformatics 2014;15(1):366; doi: 10.1186/s12859-014-0366-2. 25408143 PMC4246454

[B44] Maggiori C, Raymond-Bouchard I, Brennan L, *et al.* MinION sequencing from sea ice cryoconites leads to de novo genome reconstruction from metagenomes. Sci Rep 2021;11:21041; doi: 10.1038/s41598-021-00026-x. 34702846 PMC8548342

[B45] McCartney AM, Anderson J, Liggins L, *et al.* Balancing openness with Indigenous data sovereignty: An opportunity to leave no one behind in the journey to sequence all of life. Proc Natl Acad Sci USA 2022;119(4):e2115860119; doi: 10.1073/pnas.2115860119. 35042810 PMC8795560

[B46] Meier-Kolthoff JP, Göker M. TYGS is an automated high-throughput platform for state-of-the-art genome-based taxonomy. Nat Commun 2019;10:2182; doi: 10.1038/s41467-019-10210-3. 31097708 PMC6522516

[B47] Meier-Kolthoff JP, Göker M, Spröer C, *et al.* When should a DDH experiment be mandatory in microbial taxonomy? Arch Microbiol 2013;195:413–418; doi:10.1007/s00203-013-0888-4. 23591456

[B48] Meier-Kolthoff JP, Hahnke RL, Petersen J, *et al.* Complete genome sequence of DSM 30083(T), the type strain (U5/41(T)) of *Escherichia coli,* and a proposal for delineating subspecies in microbial taxonomy. Stand Genomic Sci 2014;10:2 doi:10.1186/1944-3277-9-2. PMC4334874 25780495

[B49] Meier-Kolthoff JP, Sardà Carbasse J, Peinado-Olarte RL, *et al.* TYGS and LPSN: A database tandem for fast and reliable genome-based classification and nomenclature of prokaryotes. Nucleic Acids Res 2022;50(D1):D801-D807; doi:10.1093/nar/gkab902. 34634793 PMC8728197

[B50] Molnar Z, Babai D. Inviting ecologists to delve deeper into traditional ecological knowledge. Trends Ecol Evol 2021;36(8):679–690; doi: 10.1016/j.tree.2021.04.006. 34024622

[B51] Morgan J, Coe RR, Lesueur R, *et al.* Indigenous peoples and genomics: Starting a conversation. J Genet Couns 2019;28(2):407–418; doi: 10.1002/jgc4.1073. 30629780 PMC7379939

[B52] Nazari-Sharabian M, Aghababaei M, Karakouzian M, *et al.* Water on Mars—A literature review. Galaxies 2020;8:40; doi:10.3390/galaxies8020040.

[B53] Ondov BD, Treangen TJ, Melsted P, *et al.* Mash: Fast genome and metagenome distance estimation using MinHash. Genome Biol 2016;17(1):132; doi: 10.1186/s13059-016-0997-x. 27323842 PMC4915045

[B54] Ormeno-Orrillo E, Martinez-Romero E. A genomotaxonomy view of the *Bradyrhizobium* genus. Front Microbiol 2019;10:1334; doi: 10.3389/fmicb.2019.01334. 31263459 PMC6585233

[B55] Papenfort K, Bassler BL. Quorum sensing signal-response systems in Gram-negative bacteria. Nat Rev Microbiol 2016;14:576–588; doi: 10.1038/nrmicro.2016.89. 27510864 PMC5056591

[B56] Parks DH, Imelfort M, Skennerton CT, *et al.* CheckM: Assessing the quality of microbial genomes recovered from isolates, single cells, and metagenomes. Genome Res 2015;25(7):1043–1055; doi: 10.1101/gr.186072.114. 25977477 PMC4484387

[B57] Payler SJ, Biddle JF, Sherwood Lollar B, *et al.* An ionic limit to life in the deep subsurface. Front Microbiol 2019;10:426. 30915051 10.3389/fmicb.2019.00426 PMC6422919

[B58] Pattengale ND, Alipour M, Bininda-Emonds ORP, *et al.* How many bootstrap replicates are necessary? J Comput Biol 2010;17:337–354. 20377449 10.1089/cmb.2009.0179

[B59] Preisner EC. Linking Microbial Phylogenetic and Functional Gene Diversity to Microbial Mat Ecosystem Function Following Environmental Disturbance. Doctoral Thesis, University of South Carolina: Columbia, SC; 2018.

[B60] Prescott RD, Decho AW. Flexibility and adaptability of quorum sensing in nature. Trends Microbiol 2020;28(6):436–444; doi: 10.1016/j.tim.2019.12.004. 32001099 PMC7526683

[B61] Prescott RD, Zamkovaya T, Donachie SP, *et al.* Islands within islands: Bacterial phylogenetic structure and consortia in Hawaiian lava caves and fumaroles. Front Microbiol 2022;13:934708; doi: 10.3389/fmicb.2022.934708. 35935195 PMC9349362

[B62] Rayne A, Blair S, Dale M*, et al*. Weaving place-based knowledge for culturally significant species in the age of genomics: Looking to the past to navigate the future. Evol Appl 2022;15(5):751–772; doi: 10.1111/eva.13367. 35603033 PMC9108313

[B63] Reddy GSN, Garcia-Pichel F. *Sphingomonas mucosissima* sp. nov. and *Sphingomonas desiccabili*s sp. nov., from biological soil crusts in the Colorado Plateau, USA. Int J Syst Evol Microbiol 2007;57(Pt 5):1028–1034; doi: 10.1099/ijs.0.64331-0. 17473253

[B64] Reyhner J, Gilbert WS, Lockard L. Honoring our Heritage: Culturally Appropriate Approaches for Teaching Indigenous Students. Northern Arizona University, Flagstaff, AZ; 2011.

[B65] Rippka R, Deruelles J, Waterbury J, *et al.* Generic assignments, strain histories and properties of pure cultures of cyanobacteria. J Gen Microbiol 1979;111(1):1–61; doi: 10.1099/00221287-111-1-1.

[B66] Ruan J, Li H. Fast and accurate long-read assembly with wtdbg2. Nat Methods 2020;17:155–158; doi: 10.1038/s41592-019-0669-3. 31819265 PMC7004874

[B67] Santomartino R, Waajen AC, de Wit W, *et al.* No effect of microgravity and simulated Mars gravity on final bacterial cell concentrations on the International Space Station: Applications to space bioproduction. Front Microbiol 2020;11:579156; doi: 10.3389/fmicb.2020.579156. 33154740 PMC7591705

[B68] Sauro F, Pozzobon R, Massironi M, *et al.* Lava tubes on Earth, Moon and Mars: A review on their size and morphology revealed by comparative planetology. Earth-Science Reviews 2020;209; doi: 10.1016/j.earscirev.2020.103288.

[B69] Schulze-Makuch D, Irwin LN, Lipps JH, *et al.* Scenarios for the evolution of life on Mars. J Geophys Res 2005;110(E12); doi: 10.1029/2005JE002430.

[B70] Seemann T. Prokka: Rapid prokaryotic genome annotation. Bioinformatics 2014;30(14):2068–2069; doi: 10.1093/bioinformatics/btu153. 24642063

[B71] Semken S. Sense of place and place-based introductory geoscience teaching for American Indian and Alaska Native undergraduates. Journal of Geoscience Education 2005;53(2):149–157; doi: 10.5408/1089-9995-53.2.149.

[B72] Stackebrandt E, Goebel BM. Taxonomic note: A place for DNA-DNA reassociation and 16S rRNA sequence analysis in the present species definition in bacteriology. Int J Syst Bacteriol 1994;44:846–849; doi: 10.1099/00207713-44-4-846.

[B73] Stamatakis A. RAxML version 8: A tool for phylogenetic analysis and post-analysis of large phylogenies. Bioinformatics 2014;30:1312–1313; doi: 10.1093/bioinformatics/btu033. 24451623 PMC3998144

[B74] Stone F, Howarth FG, Nakamura JM. Lava cave management in Hawai‘i Volcanoes National Park. Proceedings of the 2005 National Cave and Karst Management Symposium: Albany, NY; 2005.

[B75] Swofford DL. PAUP*: Phylogenetic Analysis Using Parsimony (*and Other Methods), Version 4.0 b10. Sinauer Associates, Sunderland, UK; 2002.

[B76] Tanabe TS, Dahl C. HMS-S-S: A tool for the identification of sulphur metabolism-related genes and analysis of operon structures in genome and metagenome assemblies. Molecular Ecology Resources, 2022;22:2758–2774; doi: 10.1111/1755-0998.13642. 35579058

[B77] Tian Y, Zhao Y, Yuan X, *et al.* *Dickeya fangzhongdai* sp. nov., a plant-pathogenic bacterium isolated from pear trees (*Pyrus pyrifolia*). Int J Syst Evol Microbiol 2016;66(8):2831–2835; doi: 10.1099/ijsem.0.001060. 27045848

[B78] Tindall BJ, Rosselló-Móra R, Busse HJ, *et al.* Notes on the characterization of prokaryote strains for taxonomic purposes. Int J Syst Evol Microbiol 2010; 60(Pt 1):249–266; doi: 10.1099/ijs.0.016949-0. 19700448

[B79] Vaser R, Sovic I, Nagarajan N, *et al.* Fast and accurate de novo genome assembly from long uncorrected reads. Genome Res 2017;27(5):737–746; doi: 10.1101/gr.214270.116. 28100585 PMC5411768

[B80] Watanabe N. On four new halophilic species of *Spirillum*. Bot Mag (Tokyo) 1959;72:77–86.

[B81] Waters CM, Bassler BL. Quorum sensing: Cell-to-cell communication in bacteria. Annu Rev Cell Dev Biol 2005;21:319–346; doi: 10.1146/annurev.cellbio.21.012704.131001. 16212498

[B82] Wick RR, Schultz MB, Zobel J, *et al.* Bandage: Interactive visualization of de novo genome assemblies. Bioinformatics 2015;31:3350–3352; doi: 10.1093/bioinformatics/btv383. 26099265 PMC4595904

[B83] Wick RR, Judd LM, Gorrie CL, *et al.* Unicycler: Resolving bacterial genome assemblies from short and long sequencing reads. PLoS Comput Biol 2017;13(6):e1005595; doi: 10.1371/journal.pcbi.1005595. 28594827 PMC5481147

[B84] Yoon SH, Ha SM, Kwon S, *et al.* Introducing EzBioCloud: A taxonomically united database of 16S rRNA gene sequences and whole-genome assemblies. Int J Syst Evol Microbiol 2017;67(5):1613–1617; doi: 10.1099/ijsem.0.001755. 28005526 PMC5563544

