# Supplementary material for: Bridging Place-Based Astrobiology Education with Genomics, Including Descriptions of Three Novel Bacterial Species Isolated from Mars Analog Sites of Cultural Relevance
Source: Astrobiology. 2023 Dec 20;23(12):1348–67. doi: 10.1089/ast.2023.0072 (PMC10750312; doi:10.1089/ast.2023.0072)
Supplement: Supplemental data [file Suppl_DataS1.pdf]

Discovering Genomic Data Science through Astrobiology: looking for life in the Universe
January 13th, 14th, and 15th 2018

Workshop Agenda

<u>**Day 1 (January, 13th 2018): UH Manoa Microbiology, Snyder Building**</u>

Morning Session I

8:30 - 9:00 AM: Coffee/tea "Introduce yourself"

9:00 **AM - 9:15 AM:** Astrobiology in the classroom: an interdisciplinary science and links to Genomic Data Science (GDS). *Becks Prescott*

9:15 **AM - 9:30 AM:** Pacific American Foundation WIRED program and NGSS
Derek Esibill, WIRED Program Director, Pacific American Foundation

9:30 AM - 10:15 AM:Culture-based science education in Hawai'i: Looking back to go forward. *Herb Lee, Director Pacific American Foundation*

10:15 ***AM - 10:30 AM****: Break out sessions: how can you use these concepts in your classroom? Q & A session with speakers*

10:30 AM - 10:45 AM: Coffee/tea break

Morning session II

10:45 AM - 11:00 AM: A look at NGSS. *Derek Esibill*

11:00 ***AM - 11:15 AM****: peer group discussion on NGSS, astrobiology and GDS*

11:15 AM - 11:30 AM: Nucleic Acids: the basics.
Karen Davenport & Shannon Johnson, Los Alamos National Lab

11:30 AM - 12:00 PM*:* <u>Interactive lessons: Transcription and Translation</u>
Karen Davenport & Shannon Johnson, Los Alamos National Lab

12:00 PM - 12:30: Lunch provided by PAF

Afternoon session

12:30 PM - 1:00 PM: *Advanced Studies in Genomics, Proteomics and Bioinformatics (ASGPB) lab tour.*

1:00 PM - 1:30 PM: What would DNA look like on other planets? Astrobiology, Evolution and extreme environments. *Becks Prescott*

1:30 PM - 1:30 PM: *interactive lessons: Pipette exercise & Strawberry extraction*

1:30 PM- 4:30 PM: *DNA extraction from extreme environment microbes*

4:30 PM - 5:00 PM*: Peer group discussion and Q & A*

SEE YOU TOMORROW AT 'IOLANI SULLIVAN CENTER!

Discovering Genomic Data Science through Astrobiology: looking for life in the Universe January 13th, 14th, and 15th 2018

Workshop Agenda

<u>Day 2 (January, 14th 2018): ‘Iolani Sullivan Center</u>

Morning Session I

8:30 - 9:00 AM: Coffee/tea

9:00 AM - 9:15 AM: Welcome to ‘Iolani & The Sullivan Center for Innovation and Leadership. *Yvonne Chan and Megan Kawatachi, ‘Iolani School*

9:15 AM - 9:30 AM: From DNA sample to Sequences: the “next gen” sequencing revolution. *Karen Davenport & Shannon Johnson, Los Alamos National Lab*

9:30 AM - 10:15 AM*:* Interactive lessons: Genome assembly puzzle; An Hour of Code*. Karen Davenport & Shannon Johnson, Los Alamos National Lab*

10:15 AM - 10:30 AM: Bioinformatics: computers meet DNA
Karen Davenport & Shannon Johnson, Los Alamos National Lab

10:30 AM - 10:45 AM: Coffee/tea break

Morning Session II

10:45 AM - 11:00 AM: Quantitative Insights into Microbial Ecology (QIIME): a useful tool for learning scripting in genomics. *Becks Prescott*

11:00 AM - 12:00 PM*: Interactive lab: QIIME and extreme environment microbes Becks Prescott*

12:00 PM - 12:30: Lunch provided by PAF

12:30 PM - 2:30 PM: *Interactive lab: Empowering the Development of Genomics Expertise (EDGE). Karen Davenport & Shannon Johnson, Los Alamos National Lab*

2:30 PM - 3:30 PM: *Break out sessions: careers in GDS and astrobiology (Becks), bioinformatics (Karen & Shannon), NGSS (Derek & Yvonne)*

3:30 - 4:00 PM: Conservation Genetics in your classroom
Yvonne Chan, ‘Iolani school

4:00 PM - 4:30 PM: *Break out sessions: how can you use these concepts in your classroom? Q & A session with speakers*

SEE YOU TOMORROW AT ‘IOLANI SULLIVAN CENTER!

Discovering Genomic Data Science through Astrobiology: looking for life in the Universe
January 13th, 14th, and 15th 2018

Workshop Agenda

Day 3 (January, 15th 2018): ʻIolani Sullivan Center

Morning Session I

8:30 - 9:00 AM: Coffee/tea

9:00 AM - 9:30 AM: Theory of Evolution and Astrobiology in your classroom
Becks Prescott

9:30 AM - 11:00 AM: *Making Phylogenies using chocolate or aliens!*
Becks Prescott

11:00 AM - 11:15 AM: Owning our future. *Herb Lee, Director, PAF*

11:15 AM - 11:45 AM: *Final Q & A session/peer discussion on culture-based science ed*

11:45 AM - 12:00 PM: Astrobiology and our future*. Becks Prescott*

MAHALO FOR COMING!

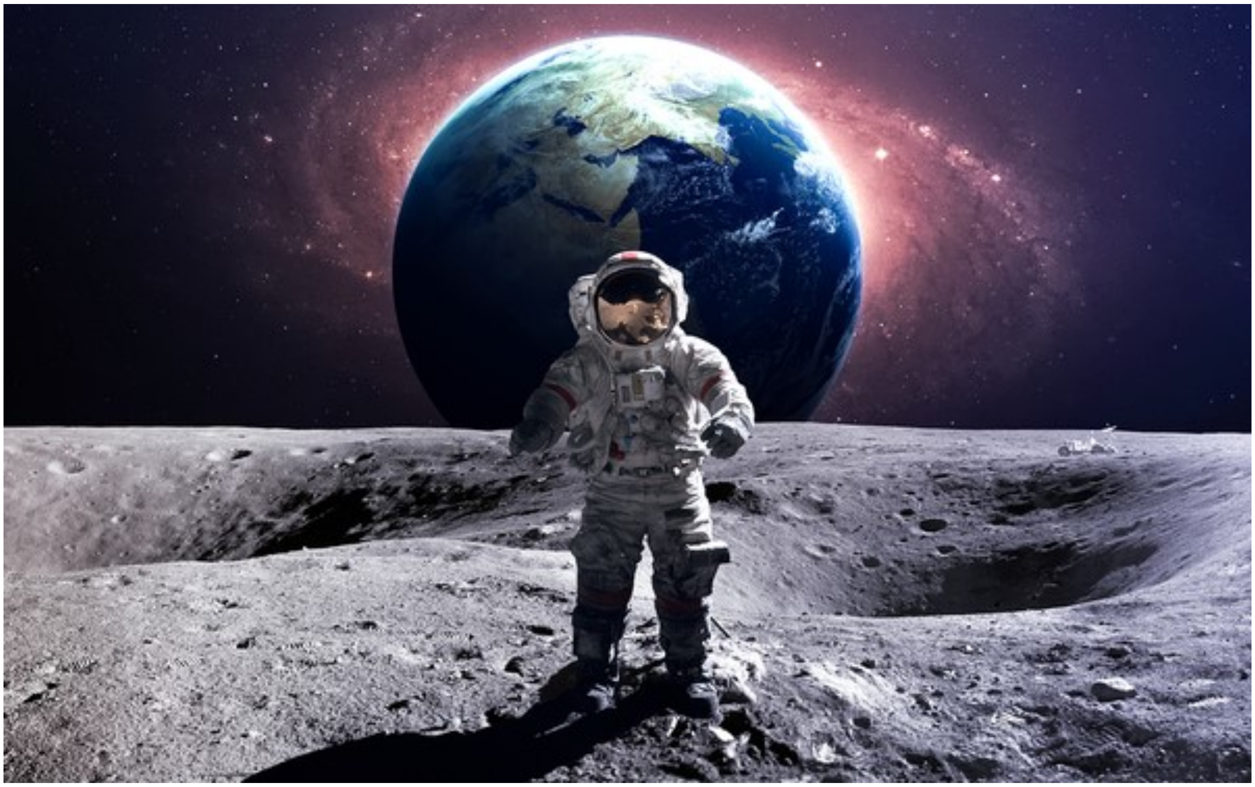

Hawaiʻi Institute of Marine Biology, University of Hawaiʻi at Mānoa GDS workshop
June 24th-28th, 2019

Day 1 – Tuesday, June 25th
Morning Session

8:30 - 9:30 AM	Coffee & Breakfast provided Coconut Island, room check in
9:30 - 9:45 AM	Orientation to Moku o Loʻe (Coconut Island)<br>*Judy Lemus, Interim Director HIMB*
9:45 - 10:00 AM	Welcome and Genomic Data Science in your classroom.<br>*Judy Lemus, HIMB and Derek Esibill, PAF*
10:00 - 10:30 AM	Culture-based science education in Hawaiʻi: Looking back to go forward. *Herb Lee, Director Pacific American Foundation*
10:30 - 10:45 AM	Coffee/tea break
10:45 - 11:00 AM	ʻIolani ʻĀina-Informatics. *Eric ʻIwakeliʻi Tong, ʻIolani School*
11:00 - 11:15 AM	A look at NGSS and discussion. *Derek Esibill, PAF*
11:15 - 11:30 AM	Nucleic Acids: the basics.<br>*Shannon Johnson, Los Alamos National Lab (LANL)*
11:30 - 12:00 PM	Interactive lesson: Transcription and Translation<br>*Shannon Johnson, LANL*
12:00 - 12:30 PM	Lunch provided

Afternoon Session

12:30 - 12:45 PM	DNA and environmental microbiology; Extraction overview<br>*Derek Esibill, PAF & Shannon Johnson, LANL*
12:45 - 1:15 PM	Interactive lab: Strawberry extraction<br>*Derek Esibill, PAF & Shannon Johnson, LANL*
1:15 - 4:15 PM	Interactive lab: DNA extraction with kits and QC check<br>*Derek Esibill, PAF & Shannon Johnson, LANL*
4:15 - 4:30 PM	ʻIolani ʻĀina-Informatics: Papaya and GMOs<br>*Eric ʻIwakeliʻi Tong, ʻIolani School*
4:30 - 5:30 PM	Group Discussion, Q&A (all team members)

Day 2 – Wednesday, June 26th

Morning Session

8:30 - 9:00 AM	Coffee and breakfast provided, Coconut Island
9:00 - 9:15 AM	PCR overview. *Androo Markham, ONT*
9:15 - 10:00 AM	Interactive lab: 16S rRNA PCR. *Androo Markham, ONT*
10:00 - 10:15 AM	Coffee/tea break
10:15 - 11:00 AM	Scotland: teaching in far off places<br>*Andrew McGrath, Plockton High school, Scotland*
11:00 - 12:00 PM	Interactive lab: PCR clean-up & quantification<br>*Androo Markham, ONT*
12:00 - 12:30 PM	Lunch provided

Afternoon Session

12:30 - 1:00 PM	From DNA to Sequences: the “next gen” sequencing revolution. *Shannon Johnson, Los Alamos National Lab*
1:00 - 1:30 PM	Intro Nanopore sequencing & what is 16S?<br>*Androo Markham, ONT*
1:30 - 2:30 PM	Interactive lab: Pooling, Library prep, loading flow cells, start MinION, *Androo Markham, ONT*
2:30 - 2:45 PM	Coffee break
2:45 - 3:15 PM	Q&A on material covered thus far (all team members)
3:15 - 3:45 PM	‘Āina-Informatics: using the MinION in your classroom<br>*Eric ‘Iwakeli‘i Tong*
3:45 - 4:00 PM	Q&A (all team members)
4:00 - 4:15 PM	Discussion for tomorrow’s activities<br>*Judy Lemus & Derek Esibill*
4:15 - 4:45 PM	Check MinION run. *Androo Markham*

Notes for Thursday morning: Please wear clothes and shoes that you don’t mind getting muddy or wet at the fishpond.

Day 3 – Thursday, June 27th.

Morning Session

7:45 - 8:00 AM	Meet at Lighthouse to leave for Waikalua Loko Fishpond<br>**Depart by 8:00 AM**
8:30 - 9:00 AM	Breakfast provided at fishpond
9:00 - 11:30 AM	<u>Outdoor activities:</u> Waikalua Loko Fishpond: Hawaiian culture-based science education & sampling your environments<br>*Derek Esibill & Herb Lee*
11:30 AM	**Depart for Coconut Island**
12:00 PM	Return to Moku o Loʻe (Coconut Island) and break for lunch
12:30 - 1:00 PM	Lunch provided

Afternoon Session

1:30 - 1:45 PM	New and Old Scots - Introduction to Scottish cultural and educational landscape. *Lewis Hou, Science Ceilidh*
1:45 - 2:00 PM	Scottish Folk Dance Demonstration *Lewis Hou, Science Ceilidh*
2:00 - 2:30 PM	Culture-based Science Education in Scotland<br>*Lewis Hou, Science Ceilidh*
2:30 - 2:45 PM	Coffee/tea break
2:45 - 3:15 PM	Hawaiian Hula: *Kamahuialani Barbett*
3:15 - 3:45 PM	<u>Interactive Workshop</u>: Cultural-based Science Education<br>*Lewis Hou & Kamahuialani Barbett*
3:45 - 4:00 PM	ʻĀina-Informatics: Bioethics through a Hawaiʻi cultural lens<br>*Eric ʻIwakeliʻi Tong, ʻIolani School*
4:00 - 5:00 PM	Discussion & Intercultural exchange; culture in your classroom<br>(all team members)
5:00 - 5:30 PM	Q&A; discuss tomorrow's plans<br>(all team members)

Day 4 – Friday, June 28th.
Morning Session

**8:00 AM**	**Move out of rooms at Coconut Island**
**8:15 - 8:30 AM**	Meet al lighthouse to depart for ʻIolani School, Honolulu
**8:30 AM**	**Depart by 8:30**
9:00 - 9:45 AM	Coffee and Breakfast provided - Iolani School
10:00 - 10:15 AM	Welcome to Sullivan Center. *Yvonne Chan, ʻIolani School*
10:15 - 10:30 AM	Bioinformatics: computers meet DNA<br>*Shannon Johnson, LANL*
10:30 - 11:30	Interactive lab: Command line basics.<br>*Androo Markham, ONT*
11:30 - 12:30	Interactive lab: What did we get? Analysis of MinION run.<br>*Androo Markham, ONT*
12:30 - 1:00 PM	Lunch provided

Afternoon Session

1:00 - 1:30 PM	GDS avenues: whole genomes vs. metagenomics vs. amplicons<br>*Shannon Johnson, LANL & Androo Markham, ONT*
1:30 - 2:00 PM	Interactive lesson: Genome assembly puzzle<br>*Shannon Johnson, LANL & Eric ʻIwakeliʻi Tong, ʻIolani School*
2:00 - 3:30 PM	Interactive lab: Empowering the Development of Genomics Expertise (EDGE). *Shannon Johnson, LANL*
3:30 - 4:00 PM	Listening session: how can we help build this into your classroom?<br>(All team members/work in small groups/discussion)
4:00 - 4:30 PM	Closing remarks (all team members)
**4:30 - 5:00 PM**	**Transport from ʻIolani to airport**

Mahalo for joining us!
