# Supplementary material for: Bridging Place-Based Astrobiology Education with Genomics, Including Descriptions of Three Novel Bacterial Species Isolated from Mars Analog Sites of Cultural Relevance
Source: Astrobiology. 2023 Dec 20;23(12):1348–67. doi: 10.1089/ast.2023.0072 (PMC10750312; doi:10.1089/ast.2023.0072)
Supplement: Supplemental data [file Suppl_DataS3.pdf]

Supplementary Figure 1. Assembly graphs of 12, genomes assembled by students and researchers using three methods: SPAdes (Illumina data only), LRASM-Miniasm (MinION data only), and Unicycler (hybrid assembly). Unicycler consistently performed better with more complete genome assemblies. Illumina data was based on sequencing depth of 50X. JS3 was sequenced with ONT technology and assembled with Lrasm-wtdbg2, which does not produce an assembly graph file. It is therefore not included in this table. Assembly graphs are one tool to access the quality of a genome assembly, and are particularly good for educational purposes.

Isolate species ID	Illumina SPAdes	lrasm-miniasm (MinION)	Hybrid Unicycler
**BIC5C1** *Paenibacillus* sp. novel	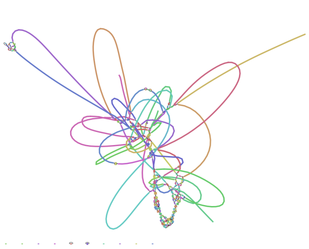	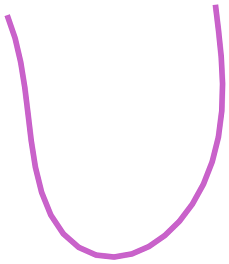	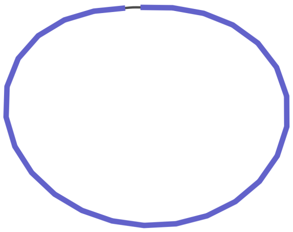
**BIC8F** *Cupriavidus* sp. novel	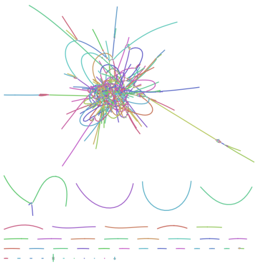	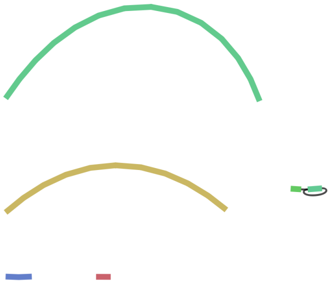	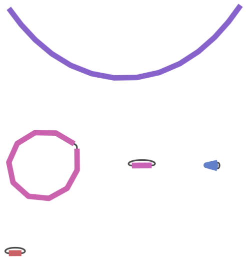
**BIC9C** *Pseudomonas* sp. novel	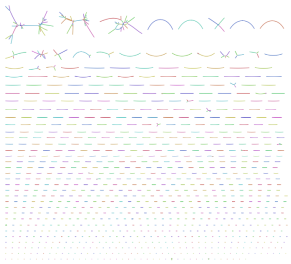	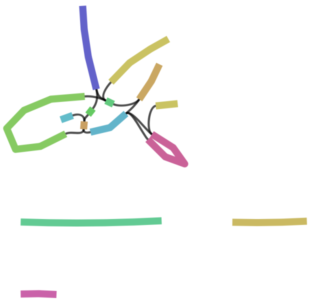	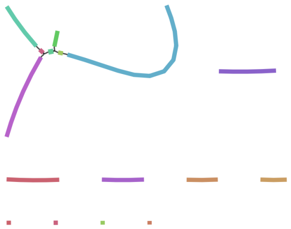

Isolate species ID	Illumina SPAdes	lrasm-miniasm (MinION)	Hybrid Unicycler
**BL16A**<br>*Bradyrhizobium prioratisuperbia* sp. novel	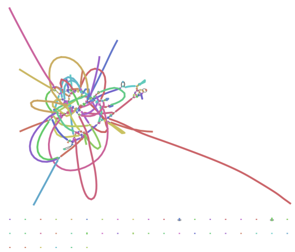	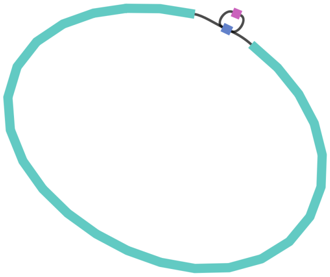	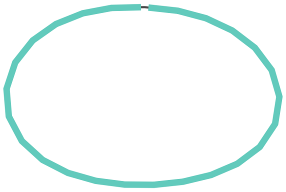
**BL16E**<br>*Pseudoflavitalea* sp. novel	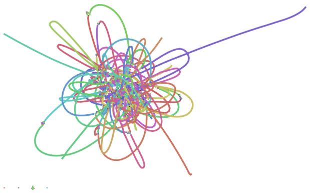	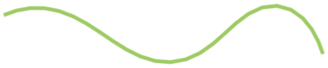	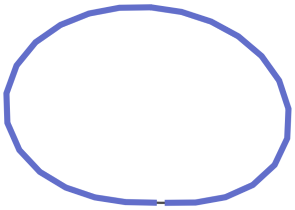
**BL38**<br>*Dermacoccus abyssi*	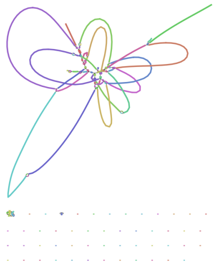	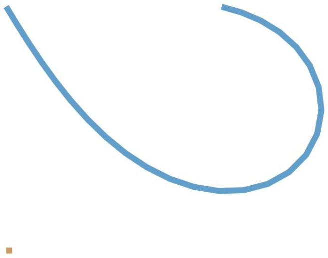	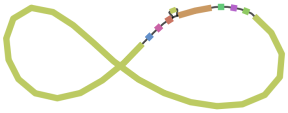
**C9-3**<br>*Pseudomonas* sp. novel	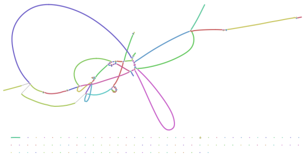	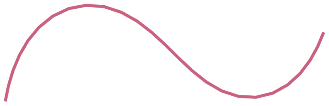	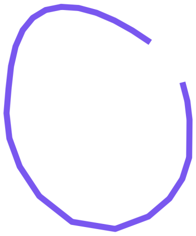

Isolate species ID	Illumina SPAdes	lrasm-miniasm (MinION)	Hybrid Unicycler
**JS2**<br>*Fischerella* sp. novel	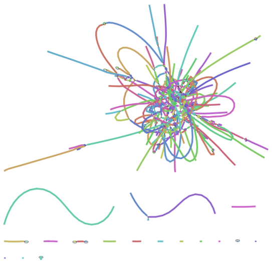	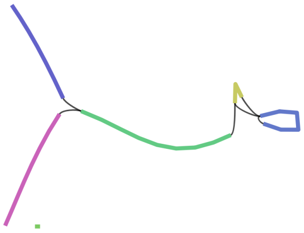	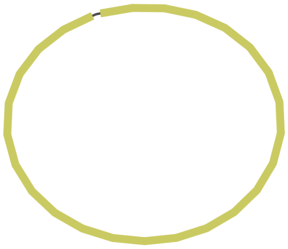
**K61**<br>*Brenneria ulupoensis* sp. novel	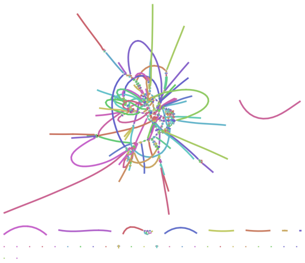	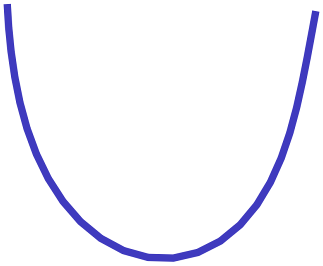	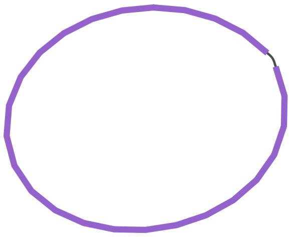
**SD**<br>*Sphingomonas desicabilis*	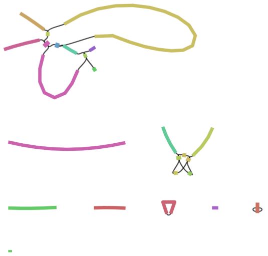	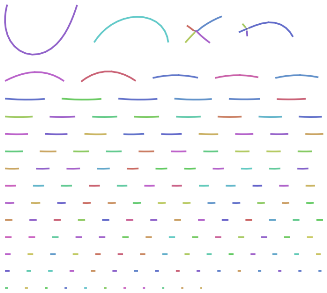	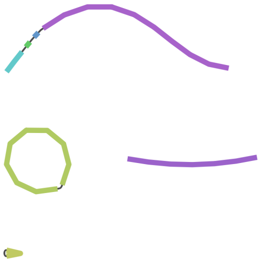

Isolate species ID	Illumina SPAdes	lrasm-miniasm (MinION)	Hybrid Unicycler
**Y38-1Y**<br>*Sphingomonas* sp. novel	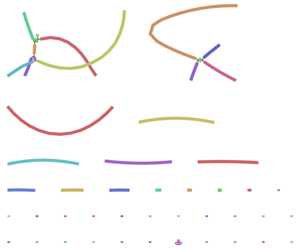	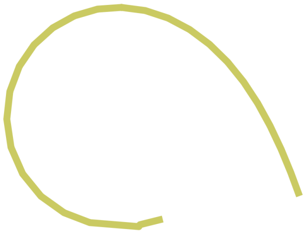	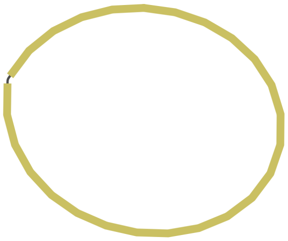
**Y88A**<br>*Rhodococcus kroppenstedtii*	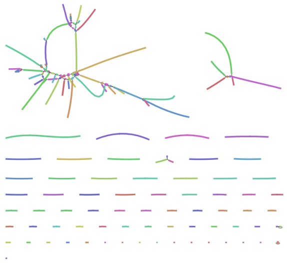	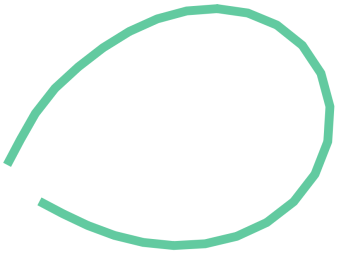	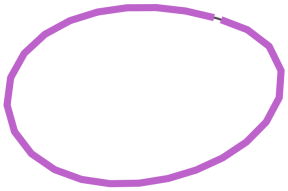
