# Supplementary material for: Bridging Place-Based Astrobiology Education with Genomics, Including Descriptions of Three Novel Bacterial Species Isolated from Mars Analog Sites of Cultural Relevance
Source: Astrobiology. 2023 Dec 20;23(12):1348–67. doi: 10.1089/ast.2023.0072 (PMC10750312; doi:10.1089/ast.2023.0072)
Supplement: Supplemental data [file Suppl_DataS4.pdf]

Table 3. Sequencing data for 13 bacterial genomes generated through MinION and Illumina by students and researchers. Total # reads for MinION data are given for subsampled data used in the best genome assemblies presented here. Mean read length for both sequencing platforms is given after QC and data that was used in assemblies. Total data generated is the total data that was generated by students and researchers before quality control (QC) or subsampling. JS3 Ilumina data was generated but was not included in assemblies because it likely has more than one organism present. Mb = Megabytes; Gb = Gigabytes.

Strain ID	Total # reads after QC		Mean read length (bp)		Total data generated	
	MinION	Illumina	MinION	Illumina	MinION	Illumina
BIC5C1	226,596	1,481,568	3054.34.	227.56	3.28 Gb	294.3 Mb
BIC8F	66,605	717,800	5201.35	171.02	1.46 Gb	167.1 Mb
BIC9C	48,403	375,270	3122.80	171.03	361.8 Mb	68.8 Mb
BL16A	81,021	2,434,258	7884.64	248.63	12.96 Gb	516.6 Mb
BL16E	194,339	2,844,786	2385.3	182.33	4.82 Gb	352.1 Mb
BL38	27,951	2,365,555	6106.98	208.87	341.1 Mb	563.7 Mb
C9-3	164,250	2,167,689	3632.56	215.56	6.19 Gb	481.5 Mb
JS2	112,664	529,712	3450.39	219.74	4.34 Gb	120.1 Mb
JS3	154,042	NA	3029.43	NA	2.1 Gb	44.6 Mb
K61	49,426	935,807	4975.98	177.29	1.06 Gb	261.4 Mb
SD	1,973,903	3,690,475	3113.62	153.24	14 Gb	987 Mb
Y38-1Y	18,844	2,225,580	4290.26	202.83	183.1 Mb	507.9 Mb
Y88A	107,255	369,235	4392.52	170.41	129.3 Mb	940.3 Mb
