# Supplementary material for: Bridging Place-Based Astrobiology Education with Genomics, Including Descriptions of Three Novel Bacterial Species Isolated from Mars Analog Sites of Cultural Relevance
Source: Astrobiology. 2023 Dec 20;23(12):1348–67. doi: 10.1089/ast.2023.0072 (PMC10750312; doi:10.1089/ast.2023.0072)
Supplement: Supplemental data [file Suppl_DataS6.pdf]

Supplementary S6: Detailed descriptions of the novel species *Bradyrhizobium prioritasuperba* BL16A^T, *Paraflavitalea speifideiaquila* BL16E^T, and *Brenneria ulupoensis* K61^T

Cultivation, morphology, and physiology methods

Cultures of *Bradyrhizobium prioritasuperba* BL16A^T, *Paraflavitalea speifideiaquila* BL16E^T, and *Brenneria ulupoensis* K61^T were passed through morphological, physiological and biochemical tests cells to test their potential designations as type strains of novel species.

The Gram reaction was determined in cells grown for 24 hr on R-2A Agar (Hi-Media Laboratories) (BL16A^T, BL16E^T), or GYE Agar (K61^T) at 30 ºC. Motility was assessed in live cells from overnight cultures (on R-2A or GYE Agar at 30 ºC) suspended in sterile distilled water, and from those grown overnight in R-2A Broth (30 ºC), under a 100x oil immersion objective on a Zeiss Axiostar Plus.The presence or absence of flagella was assessed in 48 hr cultures in R-2A (BL16A^T, BL16E^T) or GYE Broth (K61^T) by negative staining with uranyl acetate and transmission electron microscopy.

Each strain's temperature range for growth was determined on R-2A or GYE Agar at 1 ºC intervals between 4 and 40 ºC until no growth was observed within 48 hrs. The salinity range for growth was tested in R-2A or GYE Broth containing sodium chloride from 0 to 5 % w/v, at 30 ºC, with culture turbidity determined at 605 nm in a Thermo Spectronic® 20 Genesys™ spectrophotometer at ~24 hr intervals through 72 hours. The pH range for growth of BL16E^T was determined in R-2A Broth in citrate or high-pH glycine buffers from pH 4.0 to 10.8, at ~1 pH unit intervals, at 30 ºC, with turbidity at 605 nm determined at ~24 hr intervals through 72 hours (Snyder *et al*., 1997).

The capacity for microaerophilic growth was investigated in a candle jar at 30 ºC (Gerhardt *et al*., 1981); anaerobic growth was assessed at 30 ºC in the BBL GasPak® System with the BD GasPak™ EZ Anaerobe Container System Sachet; the latter produces an atmosphere containing <1 % diatomic oxygen and ≥13 % carbon dioxide within 24 h.

Single colonies of each culture were tested for catalase and cytochrome oxidase *c* with 3 % (v/v) hydrogen peroxide (Sigma) and tetramethyl-*p*-phenylenediamine discs (BBL), respectively. Amylase activity was checked on starch medium (Difco), which was flooded with 10% iodine solution after incubation at 30 ºC for 48 hours. Utilization of 71 carbon sources and sensitivity in 23 chemical assays was determined in the Biolog GEN III MicroPlate™ (Biolog Inc.). Constitutive enzyme activities were semi-quantitatively assessed in the API ZYM system (bioMérieux). K61^T was tested in the API 20E system (bioMérieux). These tests were prepared according to the manufacturers' instructions. All results were determined after incubation at 30 ºC for 24 hours; controls in each were inoculated with the cell-free diluent.

Fatty acids in whole cells of each culture grown on R-2A or GYE Agar at 30 ºC for 48 hrs were determined in the MIDI Sherlock Microbial Identification System v. 6.2, by Microbial ID, Inc. (Sasser, 1997). The nucleotide sequence of the 16S rRNA gene in each strain was compared in BLAST and EzBioCloud searches with those in type strains (Altschul *et al*., 1997; Zhang *et al*., 2000). OrthoANIu values were determined through comparison of each strain's genome sequence with the genome sequence of the nearest neighbors in 16S rRNA gene BLAST and EzBioCloud searches (Yoon *et al*., 2017).

Bradyrhizobium prioritasuperba sp. nov. BL16A^{T}

Table 1. Major and distinguishing phenotypic and genotypic characteristics of *B. prioritasuperba* BL16A^{T} and type strains of selected *Bradyrhizobium* species: strains 2-5 share highest 16S rRNA gene nucleotide sequence identities with BL16A^{T}; strains 6-10 share highest dDDH values with BL16A^{T}. **1.** *B. prioritasuperba* BL16A^{T} (data from this work); **2.** *B. algeriense* RST89^{T} (Ahnia *et al*., 2018); **3.** *B. erythrophlei* CCBAU 53325^{T} (Yao *et al*., 2015); **4.** *B. jicamae* PAC68^{T} (Ramirez-Bahena *et al*., 2009); **5.** *B. lablabi* CCBAU 23086^{T} (Chang *et al*., 2011); **6.** *B. sediminis* S2-20-1^{T} (Jin *et al*., 2022); **7.** *B. septentrionale* 1S1^{T} (Bromfield and Cloutier, 2021); **8.** *B. ivorense* CI-1B^{T} (Fossou *et al*., 2020); **9.** *B. altum* Pearl77^{T} (Avontuur *et al*., 2022); **10.** *B. uaiense* UFLA03-164^{T} (Cabral Michel *et al*., 2021). B, biofilm; N, root nodules; RS, river sediment; nr, not reported; –, negative; +, positive. All cells are Gram negative rods. Data for strains 2-10 are from the publications cited.

	**Strain**									
**Strain**	**1**	**2**	**3**	**4**	**5**	**6**	**7**	**8**	**9**	**10**
Source	B	N	N	N	N	RS	N	N	N	N
Color	White-beige	Cream	White	Pearl	Trans-lucent	Color-less	Trans-lucent	Whitish	Cream	Cream
Cell size (µm)	0.6 - 0.7 x 1.6 - 2.8	Nr	0.32 - 0.43 x 0.78 - 2.80	nr	0.5 - 0.7 x 1.0 - 7.0	0.5 - 0.6 x 1.0 – 1.4	0.8 x 2.0	0.6 - 0.7 x 1.1 - 3.4	0.45 x 1.29	nr
Growth in 1% NaCl	+	-	+	+	-	-**	-	-	+	-
Growth at 37ºC	-	-	+	-	+	-	-	-	+	+
TºC range	12-35	nr	4-60*	5-32	10-37	15-30	Nr	20-35	15-37	15-37
TºC optimum	28-30	28-30	28	28	28	25-30	~28	25-30	nr	28
DNA G+C%	63.05	62.12	59.9	64.1	60.14	63.6	63.5	64.2	63.3	63.3

*Strain #3 tolerates 60ºC for 15 mins.
**Long Jin (Y. Kim), College of Biology and the Environment, Nanjing Forestry University, pers. comm. 12/12/2022

BL16A^{T} strain grew microaerophilically in a candle jar at 30 ºC, but not in an atmosphere containing <1 % diatomic oxygen and ≥13 % carbon dioxide in the BBL GasPak® System. However, growth was noted in a 1 % diatomic oxygen atmosphere (99% N_2) in a Plas-Labs Inc. 818 Series Glove Box. Single carbon sources utilized by BL16A^{T}, as evidenced by reduction of tetrazolium redox dye in the Biolog GEN III MicroPlate™, are dextrin, D-maltose, sucrose, D-turanose, D-melibiose, α–D-glucose, D-mannose, D-fructose, D-galactose, L-fucose, L-rhamnose, inosine, D-serine, D-sorbitol, D-glucose-6-phosphate, pectin, glucuronamide, Tween 40, α-hydroxy-butyric acid, acetoacetic acid, propionic acid, sodium butyrate. Tetrazolium redox dye is also reduced in the presence of tetrazolium blue, nalidixic acid, vancomycin, aztreonam, and at pH 5. Constitutive enzymes expressed in the

API ZYM system are alkaline phosphatase, esterase (C_4), esterase lipase (C_8), and naphthol-AS-BI-phosphohydrolase. Amylase is not produced on Starch Agar after incubation at 30 ºC for 24 hours.

The predominant fatty acids in BL16A^T are consistent with those in profiles of related *Bradyrhizobium* species, specifically, high concentrations of *cis*-11- or *cis*-12-octadecenoic acid ($C_{18:1}$ ω7*c*/$C_{18:1}$ ω6*c*), with relatively higher proportions of octadecanoic acid ($C_{18:0}$), *cis*-9,10-methylenehexadecanoic acid ($C_{17:0}$ cyclo), and *cis*-10,11-methylene-octadecanoic acid ($C_{19:0}$ cyclo ω8*c*), and a lower proportion of hexadecanoic acid ($C_{16:0}$) in BL16A^T (**Table 2**). However, the Subcommittee on Taxonomy of Rhizobia and Agrobacteria of the International Committee on Systematics of Prokaryotes noted that fatty acid methyl ester (FAME) profiles are of limited use in discriminating such species (de Lajudie *et al*., 2019); this is evident in the contrasting profiles reported for cf. *B. jicamae* PAC68^T and *B. lablabi* CCBAU 23086^T (**Table 2**).

Table 2. Percentage composition of fatty acid methyl esters in whole cells of *B. prioritasuperba* BL16A^T and type strains of selected *Bradyrhizobium* species: strains 2-5a share highest 16S rRNA gene nucleotide sequence identities with BL16A^T; strains 6-10 share highest dDDH values with BL16A^T. Strains: **1.** BL16A^T (data from this work); **2.** *B. algeriense* RST89^T (Ahnia *et al*., 2018); **3.** *B. erythrophlei* CCBAU 53325^T (Yao *et al*., 2015); **4.** *B. jicamae* PAC68^T (Chang *et al*., 2011); **4a.** *B. jicamae* PAC68^T (Ahnia *et al*., 2018); **5.** *B. lablabi* CCBAU 23086^T (Chang *et al*., 2011); **5a.** *B. lablabi* CCBAU 23086^T (Ahnia *et al*., 2018); **6.** *B. sediminis* S2-20-1^T (Jin *et al*., 2022); **7.** *B. septentrionale* 1S1^T (Bromfield and Cloutier, 2021); **8.** *B. ivorense* CI-1B^T (Fossou *et al*., 2020). Fatty acid composition was not reported in the original description of *B. jicamae* (Ramírez-Bahena *et al*., 2009); two reports here are from later authors. Fatty acids not reported in the original descriptions of *B. altum* Pearl77^T (Avontuur *et al*., 2022) and *B. uaiense* UFLA03-164^T, strains 9 and 10 in Table 1, respectively. – not detected, or not reported. Data for strains 2-8 are from the publications cited. a - The MIDI system is unable to differentiate these fatty acids.

	**Strain**									
**Fatty acid**	**1**	**2**	**3**	**4**	**4a**	**5**	**5a**	**6**	**7**	**8**
$C_{9:0}$	-	0.56	-	-	0.97	-	-	-	-	-
$C_{10:0}$	-	-	-	-	-	-	0.68	-	-	-
$C_{11:0}$ 2-OH	-	-	-	2.71	-	-	-	-	-	-
$C_{11:0}$ 3-OH	-	-	-	1.66	-	0.06	-	-	-	-
$C_{12:0}$	-	-	-	-	2.11	-	4.64	-	-	-
$C_{12:0}$ *anteiso*	-	-	-	-	-	0.03	-	-	-	-
$C_{12:0}$ 2-OH	-	-	-	-	-	0.02	-	-	-	-
$C_{12:0}$ 3-OH	0.39	-	0.1	-	-	-	-	-	-	-
$C_{12:1}$ 3-OH	-	-	-	-	-	-	-	-	-	0.42
$C_{12:1}$ at 11-12	-	-	-	5.02	-	0.28	-	-	-	-
$C_{13:0}$ *iso* 3-OH	-	-	-	3.85	-	-	-	-	-	-
$C_{13:0}$ *anteiso*	-	-	-	-	-	0.02	-	-	-	-
$C_{14:0}$	0.29	0.76	0.2	-	2.45	-	-	-	-	-
$C_{14:0}$ *iso* 3-OH	-	-	-	-	-	0.04	-	-	-	-

[a]$C_{14:0}$ 3-OH/$C_{16:1}$ *iso*	-	-	0.1	-	-	-	-	-	-	-
$C_{15:1}$ *iso* F	-	-	-	-	-	0.16	-	-	-	-
[a]$C_{15:1}$ *iso* H/$C_{13:0}$ 3-OH	-	-	-	6.60	-	-	-	-	-	-
$C_{16:0}$	6.50	19.01	15.3	-	13.39	-	19.62	19.3	10.48	9.68
$C_{16:1}$ *ω9cis*	-	-	-	28.95	-	1.69	-	-	-	-
$C_{16:1}$ *ω5cis*	0.79	-	0.7	-	-	-	-	-	0.49	0.31
[a]$C_{16:1}$ *ω7c*/16:1 ω6*c*	5.37	1.2	2.3	1.93	1.32	0.15	1.23	14.0	1.20	1.83
$C_{16:1}$ *iso* H	-	-	-	1.41	-	0.06	-	-	-	-
$C_{17:0}$	-	-	0.1	-	-	-	0.55	-	-	-
$C_{17:0}$ 2-OH	-	-	-	-	-	0.03	-	-	-	-
$C_{17:0}$ cyclo	8.64	1.04	3.6	-	-	-	-	-	0.46	-
$C_{18:1}$ ω7*c* 11-methyl	5.59	10.89	0.1	-	2.79	-	2.12	-	-	-
$C_{17:1}$ ω9*c*	-	-	-	10.09	-	2.35	-	-	-	-
$C_{17:1}$ ω8*c*	-	-	0.1	-	-	-	0.92	-	-	-
$C_{17:1}$ ω7*c*	-	-	-	6.54	-	-	-	-	-	-
$C_{17:1}$ ω6*c*	-	-	-	-	-	-	0.95	-	-	-
$C_{17:0}$ *anteiso*	-	-	-	-	-	-	1.62	-	-	-
$C_{17:1}$ *anteiso* ω9*c*	-	-	0.2	-	-	-	-	-	-	-
[a]$C_{17:1}$ *anteiso* B/*iso* I	-	-	-	2.52	-	-	-	-	-	-
$C_{17:1}$ *iso* ω10*c*	-	-	-	-	-	0.03	-	-	-	-
$C_{18:0}$	10.47	1.67	0.2	-	2.86	-	3.29	-	1.72	0.4
$C_{18:0}$ *iso*	-	-	-	-	1.39	-	0.95	-	-	-
$C_{18:1}$ ω9*c*	-	-	-	-	1.18	-	2.82	-	-	-
$C_{18:1}$ ω5*c*	-	-	0.5	-	-	-	-	-	0.39	0.47
$C_{18:1}$ ω7*c*	-	-	-	-	-	94.24	-	-	-	86.03
$C_{18:1}$ ω6*c*	-	-	-	17.86	-	-	-	-	-	-
[a]$C_{18:1}$ ω7*c*/$C_{18:1}$ ω6*c*	52.66	63.30	58.4	-	70.94	-	57.74	66.7	81.98	-
[a]$C_{18:2}$ ω6*c*, 9*c*/$C_{18:0}$ *ante*	0.64	-	-	-	0.60	-	2.45	-	-	-

$C_{19:0}$	0.42	-	-	-	-	-	-	-	-	-
$C_{19:0}$ cyclo ω8*c*	8.02	1.55	18.2	-	-	-	-	-	2.76	0.86
[a]$C_{19:1}$ ω11*c*/$C_{19:1}$ ω9*c*	-	-	-	8.64	-	0.69	0.42	-	-	-
[a]$C_{19:1}$ ω7*c*/$C_{19:1}$ ω6*c*	-	-	-	2.21	-	0.17	-	-	-	-
$C_{20:0}$	-	-	-	-	-	-	-	-	0.38	-
$C_{20:2}$ *ω*6,9*c*	0.22	-	0.1	-	-	-	-	-	-	-

On the basis of 1479 nucleotides in the 16S rRNA gene, the nearest type strain neighbors of BL16A^T in the EZBioCloud 16S database are *B. algeriense* RST89^T (1274/1301, 97.9 %), *B. erythrophlei* CCBAU 53325^T (1307/1335, 97.9 %), *B. jicamae* PAC68^T (1379/1411, 97.7%), *B. lablabi* CCBAU 23086^T (1378/1411, 97.7 %), and each of *B. icense* LMTR 13^T, *B. paxllaeri* LMTR 21^T, *B. embrapense* SEMIA 6208^T, *B. viridifuturi* SEMIA 690^T, *B. namibiense* 510^T at 1377/1411 (97.6 %). The nearest type strain neighbors of BL16A^T in BLAST searches at the NCBI are *B. valentinum* LmjM3^T (1443/1479, 97.6%), *B. mercantei* SEMIA 6399^T (1435/1470, 97.6%), *B. viridifuturi* SEMIA 690^T (1435/1470, 97.6%), and *B. jicamae* PAC68^T (1441/1480, 97.4%). A maximum-likelihood (ML) tree based on 16S rRNA gene sequences extracted from the genomes of BL16A^T and related type strains on the Type Strain Genome Server (TYGS) shows BL16A^T close to *B. sediminis* S2-20-1^T (**Fig. 1**).

The assembled genome of BL16A^T was uploaded to the Type (Strain) Genome Server (TYGS) for a whole genome-based taxonomic analysis by comparison through the MASH algorithm with the genomes of all published type strains in the TYGS database (Ondov *et al.*, 2016; Wick *et al.*, 2017; Meier-Kolthoff and Göker, 2019). The analysis used recent methodological updates and features, and information on nomenclature, synonymy and associated taxonomic literature from the List of Prokaryotic names with Standing in Nomenclature (LPSN, https://lpsn.dsmz.de) (Meier-Kolthoff *et al.*, 2022). The results were provided by the TYGS on 17th November, 2022. Ten type strains with the smallest MASH distances were selected; a second ten closely related type strains were determined through comparison with the 16S rRNA gene sequence extracted from the BL16A^T genome using RNAmmer, and BLASTed against the 16S rRNA gene sequence of the 18262 type strains in the TYGS database (Lagesen and Hallin, 2007; Camacho *et al.*, 2009). The top 50 matching type strains were selected on the basis of the highest bitscores, and precise distances were calculated in the Genome BLAST Distance Phylogeny approach (GBDP) under the ‘coverage’ algorithm, and distance formula d_5 (Meier-Kolthoff *et al.*, 2013). These distances were used to determine the 10 type strain genomes closest to BL16A^T. For phylogenomic inference, pairwise comparisons among the genome set were conducted using GBDP and accurate intergenomic distances inferred under the algorithm trimming and distance formula d_5 (Meier-Kolthoff *et al.*, 2013): 100 distance replicates each were calculated, with digital DDH values and confidence intervals calculated using the recommended settings of the GGDC 3.0 (Meier-Kolthoff *et al.*, 2013, 2022). Type-based species clustering used a 70% dDDH radius around each of the 20 type strains (Meier-Kolthoff & Göker, 2019). Pairwise comparisons of the genome in digital DNA:DNA hybridizations (dDDH) with those of type strains on the TYGS showed the nearest neighbors to be *B. sediminis* S2-20-1^T (22.2%), *B.*

septentrionale 1S1^{T} (21.9%), and each of *B. ivorense* CI-1B^{T}, *B. altum* Pear77^{T}, and *B. uaiense* UFLA03-164^{T} at 21.8%, followed by *B. paxllaeri* LMTR 21^{T}, *B. acaciae* 10BB^{T}, *B. diazoefficiens* USDA 110^{T}, *B. quebecense* 66S1MB^{T}, and *B. oropedii* Pear76^{T} at 21.7% (Meier-Kolthoff and Göker 2019). The remaining 10 results returned were all *Bradyrhizobium* spp., with dDDH values ranging from 21.0% for *B. campsiandrae* INPA 394B^{T} to 21.6% dDDH for *B. centrolobii* BR 10245^{T}. dDDH values of <70% indicate the two

Fig. 1. Maximum-likelihood tree based on 16S rRNA gene sequences extracted from the genomes of BL16A^{T} and related type strains, inferred under the GTR+GAMMA model and rooted by midpoint-rooting (Hess and De Moraes Russo, 2007). Branches are scaled in terms of the expected number of substitutions per site. Scale bar represents 0.001 nucleotide substitutions per site. Numbers above branches are support values for the maximum likelihood tree (left) and maximum parsimony tree (right). Species and strain are followed in parentheses by the GenBank accession number of the 16S rRNA sequence for that strain. The input nucleotide matrix comprised 27 operational taxonomic units and 1502 characters, 91 of which were variable and 59 of which were parsimony-informative. The base-frequency check indicated no compositional bias (p = 1.00, α = 0.05). ML analysis under the GTR+GAMMA model yielded a highest log likelihood of -2988.80, whereas the estimated alpha parameter was 0.02. The ML bootstrapping did not converge, so 1000 replicates were conducted: average support was 49.83%. Maximum parsimony analysis yielded a best score of 144 (consistency index 0.72, retention index 0.86) and 50 best trees. Average support for maximum parsimony bootstrapping was 38.46%. The 16S rRNA gene sequence extracted from the BL16A^{T} genome was aligned with 16S rRNA sequences of related type strains: pairwise sequence similarities were calculated for 16S rRNA genes available through the GGDC web server at http://ggdc.dsmz.de/ (Meier-Kolthoff *et al*., 2013, 2022). Phylogenies were inferred using the DSMZ phylogenomics pipeline adapted to single genes (Meier-Kolthoff *et al*. 2014). A multiple sequence alignment was created with MUSCLE, and maximum likelihood and maximum parsimony trees inferred from the alignment with RAxML and TNT, respectively (Edgar, 2004; Goloboff *et al*., 2008; Stamatakis, 2014). Rapid bootstrapping in conjunction with the autoMRE bootstopping criterion and subsequent search for the best tree was used for maximum likelihood (Pattengale *et al*., 2010). One thousand bootstrapping replicates were used in conjunction with tree-bisection-and-reconnection branch swapping, and ten random sequence addition replicates for maximum parsimony. Sequences were checked for a compositional bias using the χ^2 test as implemented in PAUP* (Swofford, 2002).

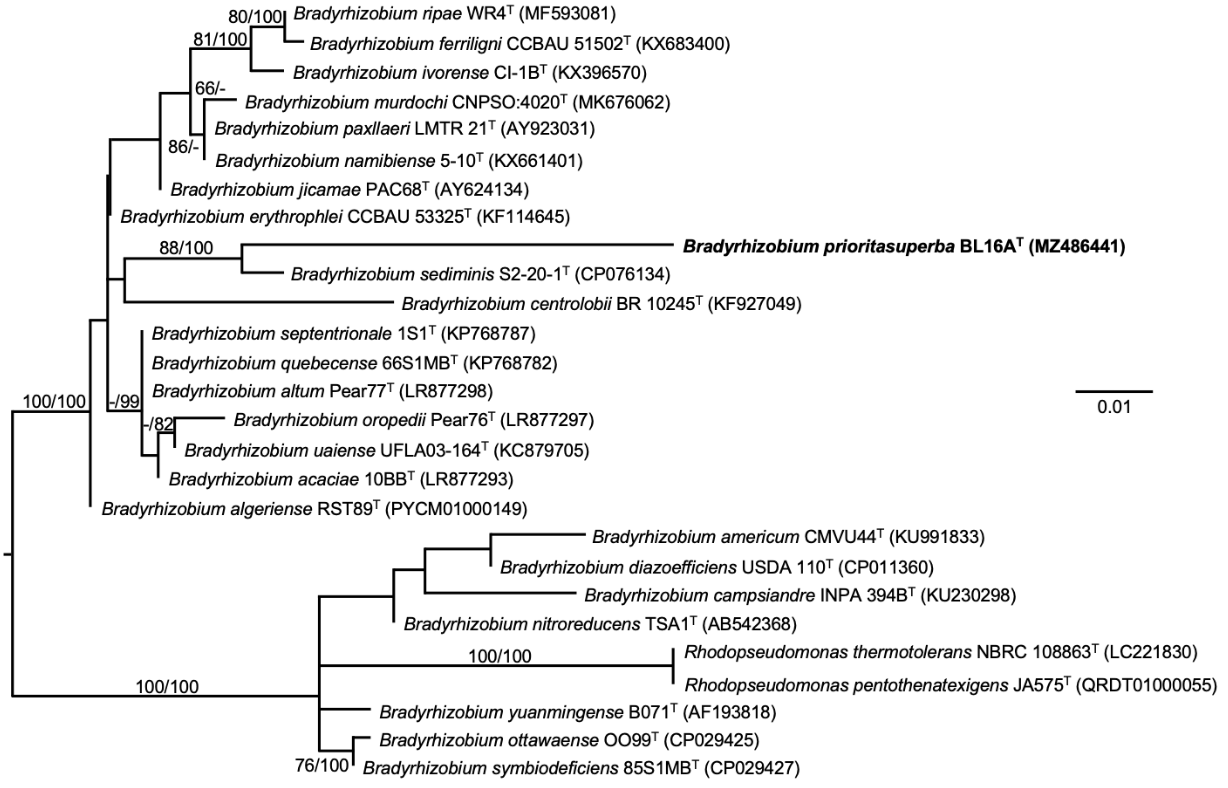

genomes compared belong to different species (Wayne *et al.*, 1987; Richter and Rosselló-Móra, 2007; Goris *et al.*, 2009; Auch *et al.*, 2010; Tindall *et al.*, 2010; Meier-Kolthoff and Göker, 2019). OrthoANIu values of below 95% also suggest that BL16A^T does not belong to an existing species in the *Bradyrhizobium* (Kim *et al.*, 2014; Yoon *et al.*, 2017; Volpiano *et al.*, 2021): highest orthoANIu values among those compared with BL16A^T were 77.43% with *B. ivorense* CI-1B^T, 77.25% with *B. viridifuturi* SEMIA 690^T, 77.09% with *B. mercantei* SEMIA 6399^T, 77.03% with *B. diazoefficiens* USDA 110^T, and 76.88 with *B. paxllaeri* LMTR 21^T.

Paraflavitalea speifideiaquila BL16E^T

Table 3. Major features and constitutive enzyme profiles of BL16E^T and type strains of related species. Strains 2 and 3 share the highest 16S rRNA gene nucleotide sequence identities with BL16E^T. 1. BL16E^T; 2. *Paraflavitalea soli* 5GH32-13T (Heo et al., 2020); 3. *Paraflavitalea devenefica* X16^T (Hou et al., 2021); 4. *Pseudoflavitalea rhizosphaerae* T16R-265^T (Kim et al., 2016); 5. *Pseudoflavitalea soli* KIS20-3^T (Kim et al., 2016); 6. *Parapseudoflavitalea muciniphila* PMP191F^T (Lawson et al., 2020). 7. *Pseudobacter ginsenosidimutans* Gsoil 221^T (Heo et al., 2020); 8. "*Flavitalea flava*" AN120636^T (Liu et al., 2019); 9. *Flavihumibacter cheonanensis* WS16^T (Kim et al., 2014b); 10. *Flavisolibacter ginsengisoli* Gsoil 643T (Yoon & Im, 2007). Aer, aerobic; M, Microaerophilic; R, rod; F, filament; C, coccus; -, negative; +, positive; +w, weakly positive; nr, not reported. Constitutive enzymes determined in the API ZYM system. Data for BL16E^T from this study. Data for strains 2-10 from the original publications.

	**Strain**									
	**1**	**2**	**3**	**4**	**5**	**6**	**7**	**8**	**9**	**10**
Cell size (μm)	0.8-0.9 x 1.5-10	1.3-1.5 x 2.5 - 8.0, 0.8 x 30	0.3-0.5 x 1.0-2.5	0.5-0.7 x 1.2-1.5, 0.3-0.5 x 7.0	0.5-0.6 x 1.0-2.5	? x 1.3-9 .8	0.4-0.7 x 3.0-3.5, 0.3-0.5 x 1.1-2.0	nr	0.5 x 0.9	0.3-0.7 x 3.0 - 6.0
Motile	-	+	-	-	-	-	-	-	-	-
Color	White	yellow	white	light yellow	yellow	oran ge-red	white	yell ow/ ora nge	yell ow	yellow
Aerobic	Aer	Aer	Aer	Aer	Aer	M	Aer	Aer	Aer	Aer
T°C range	11-37	10-37	15-40	10-40	10-37	20-4 5	10-37	15-32	15-37	15-37
pH range	5-8	6-8	5-10	6-9	5-10	5-8	5-8	5-7	6-8	5.5-8
NaCl (%, v/v) range	0	0-1	0-3	0-3	0-1	0-1	0-1	0	0-1	0-1
Shape	R	R/F	R	R	R	R	R	R/C	R	R
Catalase	-	+	-	+	+	w	+	+	+	+
Oxidase	+	-	+	+	+	+	+	-	+	-
DNA GC%	45.8	47.5	47.4	46.2	55.7	43.4	47.1	44. 8	45.9	42.7

Starch hydrolysis	-	+	+w	-	+	nr	+	-		-
Constitutive enzymes										
Alkaline phosphatase	-	+	+	+	+	+	+	+	+	+
Acid phosphatase	-	+	+	+	+	+	+	+	+	+
α−chymotrypsin	-	+	+	-	-	+	+	+	-	+
Trypsin	-	-	+	+	-	+	+	-	+	-
Esterase (C4)	+	+	+	+	+	-	+	+	-	+
Esterase lipase (C8)	-	+w	+	+	-	-	+	+w	-	+
Lipase (C14)	-	-	-	-	-	+w	-	-	-	-
Leucine arylamidase	-	+	+	+	+	+	+	+	+	+
Valine arylamidase	+	+	+	+	+	+	+	+	+	+
Cystine arylamidase	-	+	+	-	+	+	+	nr	-	+
Naphthol-AS-BI-phosphohydrolase	+	+	+	+	+	+	+	+	-	-
α-mannosidase	-	-	+	-	+	-	+	+	+	-
α-galactosidase	+	+	+	+	+	+	+	+	+	+
β-galactosidase	-	+	+	+	+	+	+	+	+	-
α-glucosidase	-	+	+	+	+	+	+	+	+	+
β-glucosidase	-	+	+	+	+	+	+	+	+	+
β-glucuronidase	-	-	-	-	-	+w	+	+w	-	-
α-fucosidase	-	-	+	+	+	-	+	+	+	+w
N-acetyl-β-glucosaminidase	+	+	+	+	+	+	+	+	+	+

Single carbon sources utilized, as evidenced by reduction of tetrazolium redox dye in the Biolog GEN III MicroPlate™, are L-serine, D-galacturonic acid, and glucuronamide. Amylase is not produced on Starch Agar after incubation at 30 ºC for 24 hours. Constitutive enzymes expressed are esterase (C4), valine arylamidase, naphthol-AS-BI-phosphohydrolase, α−galactosidase, and *N*-acetyl-β−glucosaminidase (**Table 3**). Motility was not observed in live cells, but a single polar flagellum was seen on some cells by SEM. Predominant fatty acids are 13-methyl tetradecanoic acid (C15:0 *iso*), 3-hydroxy, 15-methyl hexadecanoic acid ($C_{17:0}$ *iso* 3-OH), and g pentadecenoic acid ($C_{15:1}$ *iso* G) (**Table 4**).

Table 4. Percentage composition of fatty acid methyl esters in BL16E^{T} and type strains of species considered closely related on the basis of 16S rRNA gene nucleotide sequence identities, plus those most closely related on the basis of dDDH and orthoANI values: strains 2 and 3 share the highest 16S rRNA gene nucleotide sequence identities with BL16E^{T}. **1.** BL16E^{T} grown on R-2A medium for 24 hours at 30ºC; **2.** *Paraflavitalea soli* 5GH32-13^{T} (Heo *et al*., 2020; **3.** *Paraflavitalea devenefica* X16^{T} (Hou *et al*., 2021); **4.** *Pseudoflavitalea rhizosphaerae* T16R-265^{T} (Kim *et al*., 2016); **5.** *Pseudoflavitalea soli* KIS20-3^{T} (Kim *et al*., 2016); **6.** *Parapseudoflavitalea muciniphila* PMP191F^{T} (Lawson *et al*., 2020). **7.** *Pseudobacter ginsenosidimutans* Gsoil 221^{T} (Heo *et al*., 2020); **8.** 'Flavitalea flava' AN120636^{T} (Liu *et al*., 2019); **9.** *Flavihumibacter cheonanensis* WS16^{T} (Kim *et al*., 2014b). **10.** *Flavisolibacter ginsengisoli* Gsoil 643^{T} (Yoon & Im, 2007); Data for BL16E^{T} from this study. Data for all other strains are from the original publications. -, not reported, or not detected; tr, <0.5%.

	**Strain**									
**Fatty acid**	**1**	**2**	**3**	**4**	**5**	**6**	**7**	**8**	**9**	**10**
[a]$C_{12:0}$ aldehyde/$C_{14:0}$ 3-OH/$C_{16:1}$ *iso*	-	-	-	-	-	-	-	-	-	2.5
$C_{13:0}$ at 11-12	-	-	-	-	-	-	0.6	-	-	-
$C_{13:0}$ at 12.9999	0.3	-	-	-	-	-	-	-	-	-
$C_{13:0}$ *iso*	0.2	-	-	-	1.1	-	-	-	tr	-
$C_{13:0}$ *iso* 3-OH	-	-	-	-	-	-	-	-	-	-
$C_{14:0}$	1.6	0.6	1.2	0.6	-	-	0.7	-	tr	-
$C_{14:0}$ *iso*	-	-	-	-	-	-	0.5	-	-	-
$C_{15:0}$ 2-OH	0.6	0.5	1.1	1.5	1.0	-	0.6	-	0.5	-
$C_{15:0}$ 3-OH	0.7	-	1.6	0.6	1.0	-	0.5	-	0.6	-
$C_{15:0}$ *anteiso*	1.4	1.0	2.0	3.3	3.1	2.0	6.1	1.3	-	5.0
$C_{15:1}$ *anteiso* A	0.4	-	-	1.2	0.8	1.1	-	-	-	-
$C_{15:0}$ *iso*	39.9	37.5	37.9	33.1	27.7	34.7	42.1	16.4	31.5	31.2
$C_{15:1}$ *iso*	-	-	-	-	-	-	-	-	24.3	4.4
$C_{15:1}$ *iso* G	8.7	15.0	11.3	16.0	14.9	20.1	12.2	21.5	-	-
$C_{15:0}$ *iso* 3-OH	2.6	2.0	2.4	-	3.1	-	2.3	0.6	4.3	1.0
$C_{15:1}$ $\omega 5c$	-	-	-	-	-	4.4	-	-	-	-
$C_{16:0}$	4.0	3.4	2.5	2.9	1.4	7.6	4.1	6.5	1.6	7.5
$C_{16:0}$ 2-OH	0.2	-	-	-	-	-	-	0.7	-	2.9
$C_{16:0}$ 3-OH	5.5	4.3	3.6	2.8	2.8	1.3	-	1.9	1.6	-
$C_{16:1}$ $\omega 5c$	-	-	-	-	-	-	-	9.4	4.9	-
$C_{16:1}$ $\omega 9c$	0.1	-	-	-	-	-	-	-		-
[a]$C_{16:1}$ $\omega 7c$/16:1 $\omega 6c$	1.8	2.5	3.4	5.8	7.7	-	3.5	7.1	2.2	-

[a]$C_{16:1}$ ω7*c*/$C_{16:1}$ ω6*c* and/ or $C_{15:0}$ *iso* 2-OH	-	-	-	-	-	13.0	-	-		-
$C_{16:1}$ *ω*7*c* alcohol	0.1	0.5	-	-	-	-	0.5	-		-
$C_{16:0}$ *iso*	1.1	2.0	2.7	-	-	-	1.3	-	1.5	-
$C_{16:1}$ *iso*	-	-	-	-	-	-	-	-	tr	-
$C_{16:0}$ 10-methyl	-	-	-	-	-	-	-	-	-	2.6
$C_{16:0}$ *iso* 3-OH	1.8	2.5	3.3	0.9	1.7	-	1.2	-	0.6	-
$C_{17:0}$ 2-OH	6.7	8.2	4.7	1.9	1.8	-	1.8	2.2	1.1	-
$C_{17:0}$ 3-OH	1.0	0.9	2.6	1.1	1.6	6.1	1.0	-	0.8	-
$C_{17:0}$ *iso* 3-OH	19.8	17.9	15.4	24.1	26.0	-	17.8	26.5	16.7	11.8
$C_{17:0}$ *anteiso*	0.2	-	-	-	-	-	-	1.1	-	1.0
[a]$C_{17:1}$ *iso* ω9*c* and/or $C_{16:0}$ 10-methyl	-	-	-	-	-	-	-	-	-	-
$C_{17:0}$ *iso*	0.3	-	-	-	-	-	-	2.1	tr	9.1
[b]$C_{17:1}$ *iso* I and/or $C_{17:1}$ *anteiso* B	-	-	-	-	-	-	-	-	-	6.2[b]
[b]$C_{17:1}$ *iso* I and/or $C_{17:1}$ *anteiso*-B	-	-	-	-	-	-	-	-	-	3.1[b]
$C_{18:0}$	-	-	-	-	-	-	-	-	-	2.0
[a]$C_{18:1}$ ω7*c* or $C_{18:1}$ ω6*c*	0.2	-	-	-	-	-	-	-	-	-
[a]$C_{18:1}$ ω7*c*/$C_{18:1}$ ω9*t*/$C_{18:1}$ ω12*t*	-	-	-	-	-	-	-	-	-	1.2
$C_{18:1}$ ω9*c*	0.9	-	0.7	-	-	-	-	-	tr	-
$C_{18:2}$ ω6,9*c* and/or $C_{18:0}$ *anteiso*	-	-	-	-	-	-	-	-	-	1.7
Unknown 13.565	-	-	-	1.2	0.6	-	-	-	-	-
Unknown 16.582	-	-	-	1.6	1.8	-	-	-	-	-
[a]$C_{18:0}$ *anteiso*/$C_{18:2}$ ω6,9*c*	-	-	-	-	-	-	-	0.6	-	-

[a]The MIDI system is unable to differentiate some fatty acids. [b]This combination reported twice by the original author, with different values.

On the basis of 1418 nucleotides in the 16S rRNA gene, the nearest type strain neighbors of BL16E^T in the EZBioCloud 16S database are *Paraflavitalea soli* 5GH32-12^T (1388/1408, 98.6%), *Paraflavitalea devenifica* X16^T (1380/1408, 98.0%), *Pseudoflavitalea soli* KIS20-3^T (1342/1403, 95.7%), and *Pseudoflavitalea rhizosphaerae* T16R-265^T (1330/1402, 95.0%). The nearest type strain neighbors of BL16E^T in BLAST searches at the NCBI are *Flavitalea flava* AN120636^T (1352/1420, 95.2%), *Pseudoflavitalea soli* KIS20-3^T (1342/1407, 95.4%), *Pseudobacter ginsenosidimutans* Gsoil 221^T (1347/1418, 95.0%), and *Pseudoflavitalea rhizosphaerae* T16R-265^T (1343/1418, 94.7%). Such levels of nucleotide identity between the 16S rRNA genes in BL16E^T and its nearest neighbors support the establishment of a new species (Stackebrandt & Goebel, 1994; Stackebrandt, 2006).

A maximum likelihood tree based on 16S rRNA gene sequences extracted from the genomes of BL16E^T and related type strains on the TYGS shows BL16E^T in a distinct cluster with *Paraflavitalea soli* KACC 17331^T and *Paraflavitalea devenifica* X16^T (**Fig. 2**); these strains are also the two nearest neighbors through pairwise comparisons of the strains' genomes in dDDH.

The strain's assembled genome was uploaded to the Type (Strain) Genome Server (TYGS) for a whole genome-based taxonomic analysis by comparison through the MASH algorithm with the genomes of all published type strains in the TYGS database (Ondov *et al.*, 2016; Wick *et al.*, 2017; Meier-Kolthoff & Göker, 2019). The analysis used recent methodological updates and features, and information on nomenclature, synonymy and associated taxonomic literature from the List of Prokaryotic names with Standing in Nomenclature (LPSN, https://lpsn.dsmz.de), and the results were provided by the TYGS on 16th November, 2022 (Meier-Kolthoff *et al.*, 2022). Ten type strains with the smallest MASH distances were selected; a second ten closely related type strains were determined through comparison with the 16S rRNA gene sequence extracted from the BL16E^T genome using

Fig. 2. Maximum-likelihood tree based on 16S rRNA gene sequences extracted from the genomes of BL16E^T and related type strains, inferred under the GTR+GAMMA model and rooted by midpoint-rooting (Hess & De Moraes Russo, 2007). Branches are scaled in terms of the expected number of substitutions per site. Scale bar represents 0.001 nucleotide substitutions per site. Numbers above branches are support values for the maximum-likelihood tree (left) and maximum parsimony tree (right). Species and strain are followed in parentheses by the GenBank accession number of the 16S rRNA sequence for that strain. The input nucleotide matrix comprised 43 operational taxonomic units and 1542 characters, 386 of which were variable and 278 of which were parsimony-informative. The base-frequency check indicated no compositional bias (p = 1.00, α = 0.05). Maximum-likelihood analysis under the GTR+GAMMA model yielded a highest log likelihood of -10433.71, whereas the estimated alpha parameter was 0.15. The maximum likelihood bootstrapping converged after 900 replicates; average support was 56.70%. maximum parsimony analysis yielded a best score of 1736 (consistency index 0.34, retention index 0.55) and a single best tree. Average support for maximum-parsimony bootstrapping was 72.20%. The 16S rRNA gene sequence extracted from the BL16E^T genome was aligned with 16S rRNA sequences of related type strains: pairwise sequence similarities were calculated for 16S rRNA genes available through the GGDC web server at http://ggdc.dsmz.de/ (Meier-Kolthoff *et al*., 2013, 2022). Phylogenies were inferred using the DSMZ phylogenomics pipeline adapted to single genes (Meier-Kolthoff *et al.* 2014). A multiple sequence alignment was created with MUSCLE, and ML and MP trees inferred from the alignment with RAxML and TNT, respectively (Edgar, 2004; Goloboff *et al*., 2008; Stamatakis, 2014). Rapid bootstrapping in conjunction with the autoMRE bootstopping criterion and subsequent search for the best tree was used for maximum-likelihood (Pattengale *et al*., 2010). One thousand bootstrapping replicates were used in conjunction with tree-bisection-and-reconnection branch swapping, and ten random sequence addition replicates for maximum-parsimony. Sequences were checked for a compositional bias using the χ^2 test as implemented in PAUP* (Swofford, 2002).

RNAmmer, and BLASTed against the 16S rRNA gene sequence of the 18262 type strains in the TYGS database (Lagesen & Hallin, 2007; Camacho *et al*., 2009). The top 50 matching type strains were selected on the basis of the highest bitscores, and precise distances were calculated in the Genome BLAST Distance Phylogeny approach (GBDP) under the 'coverage' algorithm, and distance formula d_5 (Meier-Kolthoff *et al*., 2013). These distances were used to determine the 10 type strain genomes closest to BL16E^T. For phylogenomic inference, pairwise comparisons among the genome set were conducted using GBDP and accurate intergenomic distances inferred under the algorithm 'trimming' and distance formula d_5 (Meier-Kolthoff *et al*., 2013): 100 distance replicates each were calculated, with digital DDH values and confidence intervals calculated using the recommended settings of the GGDC 3.0 (Meier-Kolthoff *et al*., 2013, 2022). Type-based species clustering used a 70% dDDH radius around each of the 17 type strains (Meier-Kolthoff & Göker, 2019). Subspecies

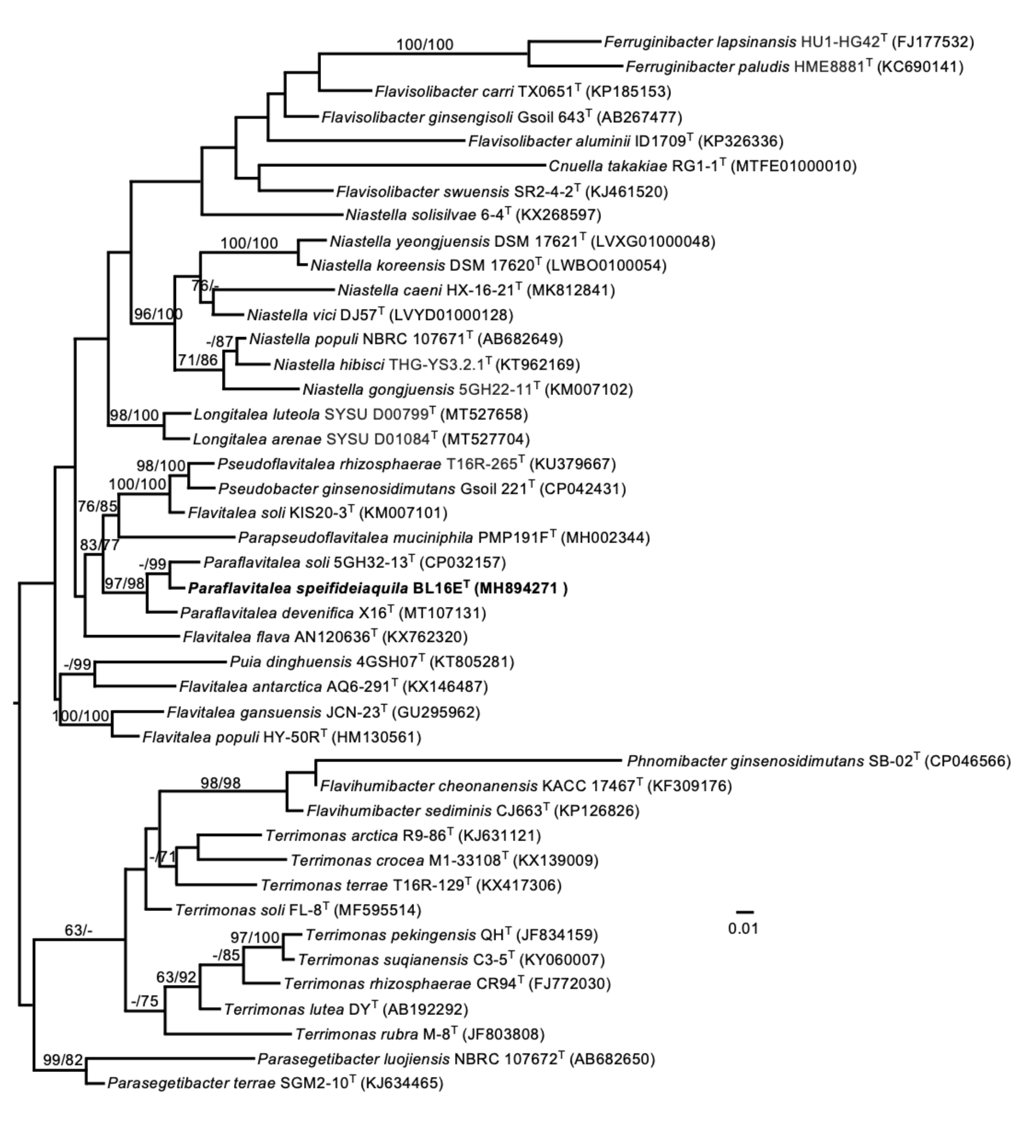

clustering was done using a 79% dDDH threshold (Meier-Kolthoff *et al*., 2014). Pairwise comparisons of the BL16E^T genome in digital DNA:DNA hybridizations (dDDH) with those of type strains on the TYGS showed the nearest neighbors to be *Paraflavitalea soli* KACC 17331^T (23.3%), *Flavisolibacter ginsengisoli* DSM 18119^T (23.1%), *Paraflavitalea devenifica* X16^T (21.5%), and *Flavihumibacter cheonanensis* WS16^T (21.3%) (Meier-Kolthoff and Göker 2019). The dDDH value with *Pseudoflavitalea rhizosphaerae* KACC 18655^T was 18.7%. dDDH values of <70% indicate the two genomes compared belong to different species (Wayne *et al*., 1987; Richter & Rosselló-Móra, 2007; Goris *et al*., 2009; Auch *et al*., 2010; Tindall *et al*., 2010; Meier-Kolthoff & Göker, 2019). OrthoANIu values of below 95% also suggest that BL16E^T does not belong to an existing species (Kim *et al*., 2014a; Yoon *et al*., 2017; Chun *et al*., 2018; Volpiano *et al*., 2021): orthoANIu values between these and BL16E^T were 79.57% with *Paraflavitalea soli* KACC 17331^T, 77.53% with *Paraflavitalea devenefica* X16^T, 68.16% with *Flavisolibacter ginsengisoli* DSM 18119^T, 64.11% with *Flavihumibacter cheonanensis* WS16^T, and 63.73% with *Pseudoflavitalea rhizosphaerae* KACC 18655^T.

***Brenneria ulupoensis* sp. nov. K61^T**

Motility was not observed in live cells, nor were flagella seen on cells observed by SEM. However, the strain does possess the genomic machinery for flagella, so the strain is likely motile in conditions not provided here.

Single carbon sources utilized, as evidenced by reduction of tetrazolium redox dye in the Biolog GEN III MicroPlate™, are dextrin, sucrose, β–D-methyl-glucoside, *N*-acetyl-D-glucosamine, α–D-glucose, D-mannose, D-fructose, D-galactose, D-mannitol, glycerol, L-aspartic acid, L-glutamic acid, L-serine, D-galacturonic acid, L-galacturonic acid, D-gluconic acid, D-saccharic acid, methyl pyruvate, citric acid, and bromo-succinic acid. Amylase is not produced on Starch Agar after incubation at 30 ºC for 24 hours. Constitutive enzymes expressed are alkaline phosphatase, acid phosphatase, esterase (C4), leucine arylamidase, naphthol-AS-BI-phosphohydrolase, β–galactosidase, and β–glucosidase. Tests positive in API 20E for production of β–galactosidase and ornithine decarboxylase, utilization of citrate as the sole carbon source nitrate reduction to nitrite, indole production from tryptophan, and fermentation of glucose, mannose, inositol, sorbitol, rhamnose, sucrose, melibiose, amygdalin, and arabinose.

Predominant fatty acids are hexadecanoic acid ($C_{16:0}$), *cis*-9-hexadecenoic acid ($C_{16:1}$ $\omega 7c$) or *cis*-10-hexadecenoic acid ($C_{16:1}$ ω6*c*), and *cis*-9,10-methylenehexadecanoic acid ($C_{17:0}$ cyclo; see **Table 5**).

Table 5. Percentage composition of fatty acid methyl esters in K61^T and type strains of species considered closely related on the basis of 16S rRNA gene nucleotide sequence identities, plus those most closely related on the basis of dDDH and orthoANI values: strains 2 and 3 share the highest 16S rRNA gene nucleotide sequence identities with K61^T. **1.** K61^T; **2.** *Brenneria bubanii* 4F2^T (Tóth and Lakatos, 2023); **3.** *Brenneria alni* NCPPB 3934^T (Li *et al*., 2019); **4.** *Brenneria corticis* CFCC 11842^T (Li *et al*., 2019); **5.** *Brenneria goodwinii* LMG 26270^T (Li *et al*., 2019); **6.** *Brenneria nigrifluens* LMG 2694^T (Li *et al*., 2019); **7.** *Brenneria populi* subsp. *populi* D9-5^T (Li *et al*., 2019); **8.** *Brenneria populi* subsp. *brevivirga* D8-10-4-5^T (Li *et al*., 2019); **9.** *Brenneria roseae* subsp. *roseae* (Brady *et al*., 2014); **10.** *Brenneria roseae* subsp. *americana* (Brady *et al*., 2014); **11.** *Brenneria tiliae* (Kile *et al*., 2022); **12.** *Dickeya oryzae* ZYY5^T (Wang *et al*., 2020); **13.** *Dickeya dadantii* subsp. *dieffenbachiae* LMG 25992^T (Brady *et al*., 2012); **14.** *Dickeya dadantii* subsp. *dadantii* LMG 25991^T (Brady *et al*., 2012); **15.** *Dickeya solani* IPO 2222^T (Tian *et al*., 2016); **16.** *Dickeya fangzhongdai* JS5^T (Tian *et al*., 2016); **17.** *Dickeya aquatica* 174/2^T (Tian *et al*., 2016); **18.** *Dickeya chrysanthemi* NCPPB 402^T (Tian *et al*., 2016); **19.** *Dickeya zeae* DSM 18068^T (Wang *et al*., 2020).

	**Strain**																		
**Fatty acid**	**1**	**2**	**3**	**4**	**5**	**6**	**7**	**8**	**9**	**10**	**11**	**12**	**13**	**14**	**15**	**16**	**17**	**18**	**19**
$C_{12:0}$	3.7	6.1	6.6	3.2	5.5	3.5	5.8	6.8	4.1	4.6	3.3	0.9	0.5	0.6	0.9	0.8	1.5	0.9	-
C12:0 aldehyde? (unknown)	8.7	-	-	-	-	-	-	-	-	-	-	-	-	-	-	-	-	-	-
C13:0	-	-	-	-	-	-	-	-	-	-	-	-	-	-	0.5	0.8	1.0	1.1	-
C13:0 3-OH/iso-C15:1 H	-	-	-	-	-	-	-	-	-	-	-	-	-	-	2.0	2.0	2.9	3.1	-
$C_{14:0}$	5.6	4.0	2.3	3.3	4.9	5.6	4.1	3.6	4.2	4.5	5.9	9.5	7.1	8.2	7.5	7.4	9.7	7.3	8.6
$C_{14:0}$ 2-OH	-	-	-	-	-	-	-	-	-	-	-	0.9	-	-	-	0.7	-	-	1.0

$C_{14:0}$ 3-OH/*iso*-$C_{16:0}$	-	-	-	-	-	-	-	-	-	-	-	7.9	-	-	-	-	-	-	8.6
$C_{14:0}$ 3-OH/*iso*-$C_{16:1}$	-	-	-	-	-	-	-	-	-	-	-	-	10.2	12.8	-	-	-	-	-
$C_{14:0}$ 3-OH/*iso*-$C_{16:1}$ I	-	-	-	-	-	-	-	-	-	-	-	-	-	-	10.5	9.3	9.3	8.6	-
$C_{14:0}$ 3-OH	-	6.7	-	8.4	11.4	11.7	9.7	7.6	8.7	8.5	8.6	-	-	-	-	-	-	-	-
$C_{15:0}$ 3-OH	-	-	-	1.1	-	-	-	1.5	14.8	15.4	-	-	-	-	-	-	-	-	-
$C_{15:1}$ ω8*c*	-	-	-	-	-	-	-	-	-	-	-	-	-	-	-	0.3	0.6	-	-
$C_{16:0}$	35.0	39.9	31.7	21.5	32.1	35.7	26.2	28.9	36.8	35.9	34.2	24.1	28.9	26.2	24.2	21.9	16.1	15.5	29.8
$C_{16:1}$ 2-OH	-	-	-	-	-	-	-	-	-	-	-	-	-	-	1.3	0.6	-	-	0.7
$C_{16:1}$ *ω7c*	-	14.3	20.5	13.9	10.4	14.4	15.4	18.4	-	-	20.4	-	-	-	-	-	-	-	-
[a]$C_{16:1}$ *ω7c*/16:1 ω6*c*	18.3	-	-	-	-	-	-	-	-	-	-	35.5	-	-	23.7	14.9	23.2	22.8	25.9
[a]$C_{16:1}$ ω7*c* and/or *iso*-$C_{15:0}$ 2-OH	-	-	-	-	-	-	-	-	-	-	-	-	34.5	36.4	-	-	-	-	-
$C_{17:0}$	0.2	-	-	-	-	-	-	-	-	-	-	-	-	-	4.3	5.6	4.3	6.2	1.2
$C_{17:1}$ ω6*c*	-	-	-	-	-	-	-	-	-	-	-	-	-	-	-	-	-	2.5	-
$C_{17:1}$ ω8*c*	-	-	-	-	-	-	-	-	-	-	-	-	-	-	2.0	2.1	4.7	6.2	-
$C_{17:0}$ cyclo	16.9	17.4	14.4	11.3	18.0	14.1	11.7	4.7	16.4	16.9	13.5	-	-	-	3.0	4.8	6.4	-	2.0
iso-$C_{17:1}$ I and/or *anteiso*-$C_{17:1}$ B	-	-	-	-	-	-	-	-	-	-	-	-	-	-	-	1.8	3.6	4.7	-
iso-$C_{17:1}$ ω5*c*	-	-	-	-	-	-	-	-	-	-	-	-	-	-	2.3	-	-	-	-
$C_{18:0}$	0.4	-	-	-	-	-	-	-	-	-	-	-	-	-	1.1	1.6	1.2	1.5	0.5
[a]$C_{18:1}$ ω7*c* or $C_{18:1}$ ω6*c*	9.4	-	-	-	-	-	-	-	-	-	-	16.4	-	-	-	-	-		17.9
$C_{18:1}$ ω7*c*	-	5.2	18.5	12.1	13.4	10.7	21.4	21.2	13.4	13.1	11.4	-	13.3	11.8	16.7	24.6	15.5	18.6	-
$C_{19:0}$ cyclo ω8*c*	0.7	3.0	1.5	1.1	2.2	2.4	2.1	-	-	-	-	-	-	-	-	-	-	1.0	-

Fatty acids for which just one of the 19 strains contained ≤0.5% of the total are not shown.
[a]The MIDI system is unable to differentiate some fatty acids.

On the basis of 1529 nucleotides in the 16S rRNA gene, the nearest type strain neighbors in the EZBioCloud 16S database are *Dickeya dadantii* subsp. *dadantii* NCPPB 898^{T} (1412/1462, 96.58%), *Dickeya dadantii* subsp. *dieffenbachiae* LMG 25992^{T} (1412/1462, 96.58%), *Dickeya dianthicola* CFBP 1200^{T} (1372/1422, 96.48%), *Dickeya solani* IPO 2222^{T} (1410/1462, 96.44%), *Dickeya fangzhongdai* JS5^{T} (1365/1421, 96.06%), and *Brenneria roseae* subsp. *roseae* FRB 222T (1288/1342, 95.98%). The nearest type strain neighbors of K61^{T} in BLAST searches at the NCBI are *Musicola keenii* A3967^{T} (1481/1531, 96.73%), *Dickeya oryzae* ZYY5^{T} (1479/1536, 96.29%), *Dickeya dadantii* subsp. *dieffenbachiae* LMG 25992^{T} (1448/1498, 96.66%), *Pectobacterium parmentieri* RNS 08-42-1A^{T} (1461/1534, 95.24%), and *Brenneria nigrifluens* DSM 30175^{T} (1442/1505, 95.81%).

An maximum likelihood phylogenetic tree based on 16S rRNA gene sequences extracted from the genomes of K61^{T} and related type strains on the TYGS showed K61^{T} as closest to *Musicola keenii* A3967^{T} and *Brenneria rubrifaciens* ATCC 29291^{T} (**Fig. 3**). However, the former placed eighteenth among 19 strains on the basis of percentage dDDH determined in TYGS. The genus *Musicola* was recently established through reclassification of *Dickeya paradisiaca* as *Musicola paradisiaca* comb. nov., and the description of *Musicola keenii* sp. nov. (Hugouvieux-Cotte-Pattat *et al*., 2021a). In this respect, 10 *Dickeya* species are also among the 19 top dDDH results. Moreover, 18 strains in the ML tree were within 1.5% dDDH (19.6 - 21.1%) of each other, while the nineteenth, *Brenneria rubrifaciens* ATCC 29291^{T}, was not among the 19 returned in the dDDH results.

Fig. 3. Maximum-likelihood tree based on 16S rRNA gene sequences extracted from the genomes of K61^{T} and related type strains, inferred under the GTR+GAMMA model and rooted by midpoint-rooting (Hess & De Moraes Russo, 2007). Branches are scaled in terms of the expected number of substitutions per site. Scale bar represents 0.001 nucleotide substitutions per site. Numbers above branches are support values for the maximum-likelihood tree (left) and maximum-parsimony tree (right). Species and strain are followed in parentheses by the GenBank accession number of the 16S rRNA sequence for that strain. The input nucleotide matrix for the 16S rRNA gene phylogenetic tree comprised 20 operational taxonomic units and 1548 characters, 146 of which were variable and 106 of which were parsimony-informative. The base-frequency check indicated no compositional bias ($p = 1.00$, $\alpha = 0.05$). The maximum likelihood analysis under the GTR+GAMMA model yielded a highest log likelihood of -4149.73, while the estimated α–parameter was 0.02. Maximum-likelihood bootstrapping did not converge, so 1000 replicates were conducted; average support was 42.18%. The maximum parsimony analysis yielded a best score of 363 (consistency index 0.50, retention index 0.60) and 2 best trees. Average support for maximum parsimony bootstrapping was 54.71%. The 16S rRNA gene sequence extracted from the K61^{T} genome was aligned with 16S rRNA sequences of related type strains: pairwise sequence similarities were calculated for 16S rRNA genes available through the GGDC web server at http://ggdc.dsmz.de/ (Meier-Kolthoff *et al*., 2013, 2022). Phylogenies were inferred using the DSMZ phylogenomics pipeline adapted to single genes (Meier-Kolthoff *et al.* 2014). A multiple sequence alignment was created with MUSCLE, and maximum-likelihood and maximum-parsimony trees inferred from the alignment with RAxML and TNT, respectively (Edgar, 2004; Goloboff *et al*., 2008; Stamatakis, 2014). Rapid bootstrapping in conjunction with the autoMRE bootstopping criterion and subsequent search for the best tree was used for maximum-likelihood (Pattengale *et al*., 2010). One thousand bootstrapping replicates were used in conjunction with tree-bisection-and-reconnection branch swapping, and ten random sequence addition replicates for MP. Sequences were checked for a compositional bias using the χ^2 test as implemented in PAUP* (Swofford, 2002).

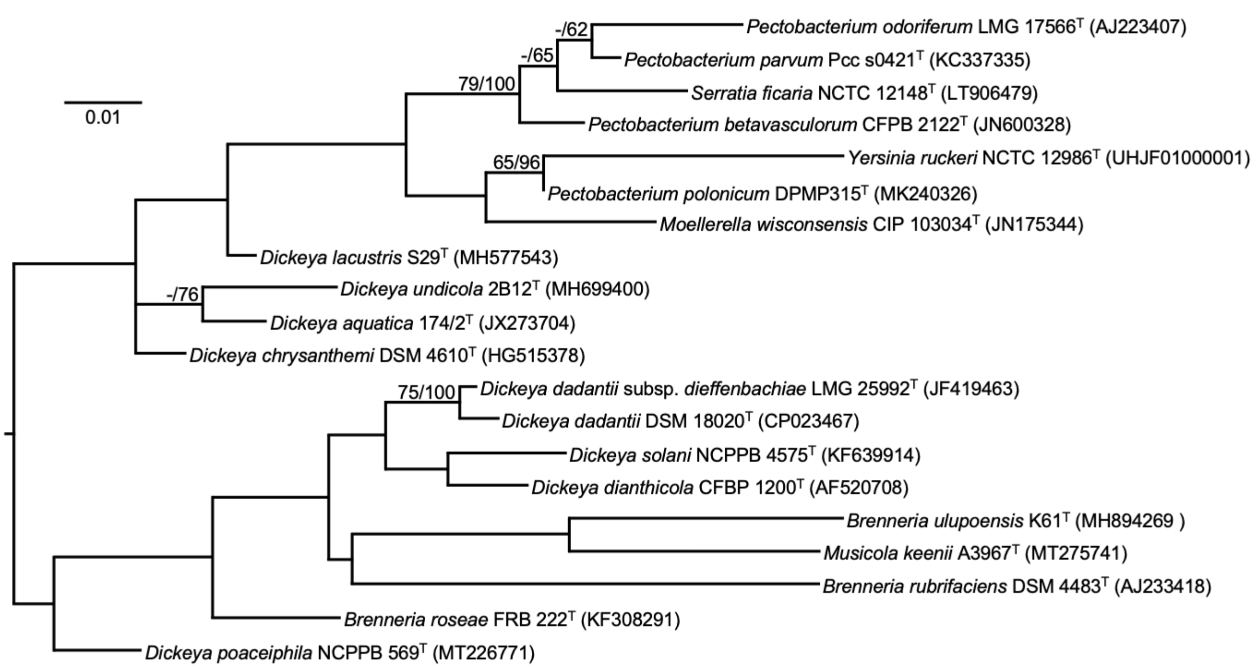

The assembled genome was uploaded to the Type (Strain) Genome Server (TYGS) for a whole genome-based taxonomic analysis by comparison through the MASH algorithm with the genomes of all published type strains in the TYGS database (Ondov *et al*., 2016; Wick *et al*., 2017; Meier-Kolthoff & Göker, 2019). The analysis used recent methodological updates and features, and information on nomenclature, synonymy and associated taxonomic literature from the List of Prokaryotic names with Standing in Nomenclature (LPSN, https://lpsn.dsmz.de), and the results were provided by the TYGS on 17th November, 2022 (Meier-Kolthoff *et al*., 2022). Ten type strains with the smallest MASH distances were selected; a second ten closely related type strains were determined through comparison with the 16S rRNA gene sequence extracted from the K61^T genome using RNAmmer, and BLASTed against the 16S rRNA gene sequence of the 18262 type strains in the TYGS database (Lagesen & Hallin, 2007; Camacho *et al*., 2009). The top 50 matching type strains were selected on the basis of the highest bitscores, and precise distances were calculated in the Genome BLAST Distance Phylogeny approach (GBDP) under the 'coverage' algorithm, and distance formula d_5 (Meier-Kolthoff *et al*., 2013). These distances were used to determine the 10 type strain genomes closest to K61^T. For phylogenomic inference, pairwise comparisons among the genome set were conducted using GBDP and accurate intergenomic distances inferred under the algorithm trimming and distance formula d_5 (Meier-Kolthoff *et al*., 2013): 100 distance replicates each were calculated, with digital DDH values and confidence intervals calculated using the recommended settings of the GGDC 3.0 (Meier-Kolthoff *et al*., 2013, 2022). Type-based species clustering used a 70% dDDH radius around each of the 19 type strains (Meier-Kolthoff & Göker, 2019). Subspecies clustering was done using a 79% dDDH threshold (Meier-Kolthoff *et al*., 2014). Pairwise comparisons of the K61^T genome in digital DNA:DNA hybridizations (dDDH) with the genomes of type strains on the TYGS showed the nearest neighbors to be *Brenneria roseae* LMG27714^T (21.1%), *Moellerella*

wisconsensis ATCC 35017^{T} (21.0%), *Dickeya solani* IPO 2222^{T} (20.9%), and each of *Pectobacterium parvum* s0421^{T}, *Pectobacterium odoriferum* NCPPB 2795^{T}, *Pectobacterium betavasculorum* NCPPB 2795^{T}, *Pectobacterium polonicum* DPMP315^{T}, and *Dickeya dianthicola* NCPPB 453^{T} at 20.7% (Meier-Kolthoff and Göker, 2019). These were followed by eight *Dickeya* species and subspecies at 20.1 – 20.5%. dDDH values of <70% indicate the two genomes compared belong to different species (Wayne *et al*., 1987; Richter & Rosselló-Móra, 2007; Goris *et al*., 2009; Auch *et al*., 2010; Tindall *et al*., 2010; Meier-Kolthoff & Göker, 2019). Genome size and GC% are given in **Table 6** for 28 related species.

Table 6. Major features of the genomes of K61^{T} and type strains of related species. Strains 2 and 3 share the highest 16S rRNA gene nucleotide sequence identities with K61^{T}. All extant *Musicola* (2, 3), *Dickeya* (4-16), and *Brenneria* spp. (17-28) are shown. **1.** K61^{T}; **2.** *Musicola keenii* A3967^{T} (Hugouvieux-Cotte-Pattat *et al*., 2021a); **3.** *Musicola paradisiaca* NCPPB 2511^{T} (Hugouvieux-Cotte-Pattat *et al*., 2021a) =*Dickeya paradisiaca* NCPPB 2511^{T}; **4.** *Dickeya aquatica* 174/2^{T} (Parkinson *et al*., 2014); **5.** *Dickeya chrysanthemi* NCPPB 402^{T} (Samson *et al*., 2005); **6.** *Dickeya dadantii* DSM 18020^{T} (Samson *et al*., 2005); **7.** *Dickeya dianthicola* CFBP 2015^{T} (Samson *et al*., 2005); **8.** *Dickeya dieffenbachiae* (Samson *et al*., 2005)/*Dickeya dadantii* subsp. *dieffenbachiae* NCPPB 2976^{T} (Brady *et al*., 2012; Pritchard *et al*., 2013); **9.** *Dickeya fangzhongdai* DSM 101947^{T} (Tian *et al*., 2016); **10.** *Dickeya lacustris* LMG 30899^{T} (Hugouvieux-Cotte-Pattat *et al*., 2019); **11.** *Dickeya oryzae* ZYY5^{T} (Wang *et al*., 2020); **12.** *Dickeya parazeae* S31^{T} (Hugouvieux-Cotte-Pattat *et al*., 2021b); **13.** *Dickeya poaceiphila* NCPPB 569^{T} (Hugouvieux-Cotte-Pattat *et al*., 2020); **14.** *Dickeya solani* IPO 2222^{T} (van der Wolf *et al*., 2014); **15.** *Dickeya undicola* 2B12^{T} (Oulghazi *et al*., 2019); **16.** *Dickeya zeae* NCPPB 2538^{T} (Samson *et al*., 2005); **17.** *Brenneria alni* NCPPB 3934^{T} (Hauben *et al*., 1998); **18.** *Brenneria bubanii* 4F2^{T} (Tóth and Lakatos, 2023); **19.** *Brenerria corticis* CFCC 11842^{T} (Li *et al*., 2019); **20.** *Brenneria goodwinii* FRB 141^{T} (Denman *et al*., 2012); **21.** *Brenneria izadpanahii* Iran 50^{T} (Bakhshi Ganje *et al*., 2021); **22.** *Brenneria izibisi* KBI 423^{T} (Gasic *et al*., 2022); **23.** *Brenneria nigrifluens* ATCC 13028^{T} (Hauben *et al*., 1998); **24.** *Brenneria populi* D9-5^{T} (Li *et al*., 2015); **25.** *Brenneria roseae* LMG 27714^{T} (Brady *et al*., 2014); **26.** *Brenneria rubrifaciens* ATCC 29291^{T} (Hauben *et al*., 1998); **27.** *Brenneria salicis* ATCC 15712^{T} (Hauben *et al*., 1998); **28.** *Brenneria tiliae* WC1b.1^{T} (Kile *et al*., 2022). Data for K61^{T} from this study. Data for other strains from genome sequences of the type strain specified. n/a, not available.

**Strain**	**1**	**2**	**3**	**4**	**5**	**6**	**7**	**8**	**9**	**10**	**11**	**12**	**13**	**14**
bp	5,052,060	4,390,312	4,689,459	4,501,560	4,700,353	4,997,541	4,785,831	4,810,532	5,032,447	4,367,773	4,587,177	4,711,277	4,317,154	4,919,833
GC%	49.3	54.4	55.0	53.6	54.3	56.4	55.74	56.5	56.8	53.1	53.4	53.7	52.7	56.2
**Strain**	**15**	**16**	**17**	**18**	**19**	**20**	**21**	**22**	**23**	**24**	**25**	**26**	**27**	**28**
bp	4,349,790	4,563,790	4,126,956	4,887,228	5,236,912	5,360,730	5,330,697	3,896,770	4,891,702	n/a	4,508,067	4,030,443	4,045,881	5,161,293
GC%	54.5	53.6	51.1	52.9	56.2	53.1	53.7	51.8	55.9	54.9-55.7	51.6	52.3	52.0	56.5

References

Ahnia H, Bourebaba Y, Duran D, Boulila F, Palacios JM, Rey L, Ruiz-Argueso T, Boulila A, Imperial J (2018) *Bradyrhizobium algeriense* sp. nov., a novel species isolated from effective nodules of *Retama sphaerocarpa* from Northeastern Algeria. *Syst Appl Microbiol* 41:333-339

Altschul SF, Madden TL, Schäffer AA, *et al*. Gapped BLAST and PSI-BLAST: a new generation of protein database search programs. *Nucleic Acids Res* 1997; 25: 3389–3402 https://doi.org/10.1093/nar/25.17.3389

Auch AF, Jan M, von Klenk H-P, Göker M (2010) Digital DNA-DNA hybridization for microbial species delineation by means of genome-to-genome sequence comparison. *Stand Genomics Sci* 2:117–134

Avontuur JR, Palmer M, Beukes CW, *et al.* (2022) *Bradyrhizobium altum* sp. nov., *Bradyrhizobium oropedii* sp. nov. and *Bradyrhizobium acaciae* sp. nov. from South Africa show locally restricted and pantropical *nodA* phylogeographic patterns. *Mol Phylogenet Evol* 167:107338 https://doi.org/10.1016/j.ympev.2021.107338

Bakhshi Ganje M, Mackay J, Nicolaisen M, *et al*. Comparative genomics, pangenome, and phylogenomic analyses of *Brenneria* spp., and delineation of *Brenneria izadpanahii* sp. nov. *Phytopathology*. 2021; 111:78-95. doi: 10.1094/PHYTO-04-20-0129-FI.

Brady CL, Cleenwerck I, Denman S, *et al*. Proposal to reclassify *Brenneria quercina* (Hildebrand and Schroth 1967) Hauben et al. 1999 into a new genus, *Lonsdalea* gen. nov., as *Lonsdalea quercina* comb. nov., descriptions of *Lonsdalea quercina* subsp. *quercina* comb. nov., *Lonsdalea quercina* subsp. *iberica* subsp. nov. and *Lonsdalea quercina* subsp. *britannica* subsp. nov., emendation of the description of the genus *Brenneria*, reclassification of *Dickeya dieffenbachiae* as *Dickeya dadantii* subsp. *dieffenbachiae* comb. nov., and emendation of the description of *Dickeya dadantii*. *Int J Syst Evol Microbiol* 2012; 62:1592-1602.

Brady C, Hunter G, Kirk S, *et al*. Description of *Brenneria roseae* sp. nov. and two subspecies, *Brenneria roseae* subspecies roseae ssp. nov and *Brenneria roseae* subspecies americana ssp. nov. isolated from symptomatic oak. *Syst Appl Microbiol* 2014; 37:396-401.

Bromfield ESP and Cloutier S (2021) *Bradyrhizobium septentrionale* sp. nov. (sv. septentrionale) and *Bradyrhizobium quebecense* sp. nov. (sv. septentrionale) associated with legumes native to Canada possess rearranged symbiosis genes and numerous insertion sequences. *Int J Syst Evol Microbiol* 71:004831 DOI 10.1099/ijsem.0.004831

Cabral Michel D, Azarias Guimaraes A, Martins da Costa E, *et al.* (2021) *Bradyrhizobium uaiense* sp. nov., a new highly efficient cowpea symbiont. *Arch Microbiol* 2020 202:1135-1141 https://doi.org/10.1007/s00203-020-01827-w

Camacho, C., Coulouris, G., Avagyan, V. *et al.* BLAST+: architecture and applications. *BMC Bioinformatics* 2009;10 421 https://doi.org/10.1186/1471-2105-10-421.

Chang YL, Wang JY, Wang ET, *et al*. (2011) *Bradyrhizobium lablabi* sp. nov., isolated from effective nodules of *Lablab purpureus* and *Arachis hypogaea*. *Int J Syst Evol Microbiol* 61:2496-2502

Chun J, Oren A, Ventosa A, *et al*. (2018) Proposed minimal standards for the use of genome data for the taxonomy of prokaryotes. *Int J Syst Evol Microbiol* 68:461-466

de Lajudie PM, Andrews M, Ardley J, *et al.* (2019) Minimal standards for the description of new genera and species of rhizobia and agrobacteria. *Int J Syst Evol Microbiol* 69:1852-1863 DOI 10.1099/ijsem.0.003426

Denman S, Brady C, Kirk S, *et al*. *Brenneria goodwinii* sp. nov., associated with acute oak decline in the UK. *Int J Syst Evol Microbiol* 2012; 62:2451-2456.

Edgar RC (2004) MUSCLE: multiple sequence alignment with high accuracy and high throughput. *Nucleic Acids Res* 32:1792-1797 doi:10.1093/nar/gkh340

Fossou RK, Pothier JF, Zeze A, *et al.* (2020) *Bradyrhizobium ivorense* sp. nov. as a potential local bioinoculant for *Cajanus cajan* cultures in Cote d'Ivoire. *Int J Syst Evol Microbiol* 70:1421-1430 DOI 10.1099/ijsem.0.003931

Gasic K, Zlatkovic N, Kuzmanovic N. Polyphasic study of phytopathogenic bacterial strains associated with deep bark canker of walnut in Serbia revealed a new species, *Brenneria izbisi* sp. nov. *Front Plant Sci* 2022; 13:1055186.

Gerhardt P, Murray RGE, Costilow RN, *et al.* (editors). *Manual of Methods for General Bacteriology*. Washington, DC: American Society for Microbiology; 1981. 524 pp.

Goloboff PA, Farris JS, Nixon KC (2008) TNT, a free program for phylogenetic analysis. Cladistics 24:774-786 doi:10.1111/j.1096-0031.2008.00217.x

Goris J, Konstantinidis KT, Klappenbach JA, *et al.* (2007) DNA–DNA hybridization values and their relationship to whole-genome sequence similarities. *Int J Syst Evol Microbiol* 57:81–91

Hauben L, Moore ER, Vauterin L, *et al*. Phylogenetic position of phytopathogens within the *Enterobacteriaceae*. *Syst Appl Microbiol* 1998; 21; 384-397.

Heo J, Weon HY, Cho H, *et al. Paraflavitalea soli* gen. nov., sp. nov., isolated from greenhouse soil. *J Microbiol* 2020; 58:17-23.

Hou X, Liu H, Shang Y, *et al. Paraflavitalea devenefica* sp. nov., isolated from urban soil. *Int J Syst Evol Microbiol* 2021; 71:4587.

Hess PN and De Moraes Russo CA (2007) An empirical test of the midpoint rooting method. *Biol J Linn Soc* 92:669-674 doi:10.1111/j.1095-8312.2007.00864.x

Hugouvieux-Cotte-Pattat N, Brochier-Armanet C, Flandrois JP, *et al. Dickeya poaceiphila* sp. nov., a plant-pathogenic bacterium isolated from sugar cane (*Saccharum officinarum*). *Int J Syst Evol Microbiol* 2020; 70:4508-4514.

Hugouvieux-Cotte-Pattat N, Jacot des-Combes CJ, Briolay J, *et al*. Proposal for the creation of a new genus *Musicola* gen. nov., reclassification of *Dickeya paradisiaca* (Samson *et al*. 2005) as *Musicola paradisiaca* comb. nov. and description of a new species *Musicola keenii* sp. nov. *Int J Syst Evol Microbiol* 2021a;71: 5037

Hugouvieux-Cotte-Pattat N, Jacot-des-Combes C, Briolay J. *Dickeya lacustris* sp. nov., a water-living pectinolytic bacterium isolated from lakes in France. *Int J Syst Evol Microbiol* 2019; 69:721-726.

Hugouvieux-Cotte-Pattat N, Van Gijsegem F. Diversity within the *Dickeya zeae* complex, identification of *Dickeya zeae* and *Dickeya oryzae* members, proposal of the novel species *Dickeya parazeae* sp. nov. *Int J Syst Evol Microbiol* 2021b; 71:5059.

Jin C-Z, Wu X-W, Zhuo Y, *et al.* (2022) Genomic insights into a free-living, nitrogen-fixing but non nodulating novel species of *Bradyrhizobium sediminis* from freshwater sediment: three isolates with the smallest genome within the genus *Bradyrhizobium*. *Syst Appl Microbiol* 45:126353 https://doi.org/10.1016/j.syapm.2022.126353.

Kile H, Arnold D, Allainguilaume J, *et al. Brenneria tiliae* sp. nov., isolated from symptomatic *Tilia* x *moltkei* and *Tilia* x *europaea* trees in the UK. *Int J Syst Evol Microbiol* 2022; 72:5515.

Kim M, Oh H-S, Park S-C, *et al.* (2014a) Towards a taxonomic coherence between average nucleotide identity and 16S rRNA gene sequence similarity for species demarcation of prokaryotes. *Int J Syst Evol Microbiol* 64:346-351

Kim SJ, Cho H, Ahn JH, *et al. Pseudoflavitalea rhizosphaerae* gen. nov., sp. nov., isolated from rhizosphere of tomato, and proposal to reclassify *Flavitalea soli* as *Pseudoflavitalea soli* comb. nov. *Int J Syst Evol Microbiol* 2016; 66:4167-4171.

Kim WH, Lee S, Ahn TY (2014b) *Flavihumibacter cheonanensis* sp. nov., isolated from sediment of a shallow stream. *Int J Syst Evol Microbiol* 64:3235-3239

Lagesen K and Hallin P. RNAmmer: consistent and rapid annotation of ribosomal RNA genes. *Nucleic Acids Res* 2007;35: 3100–3108.

Lawson PA, Patel NB, Mohammed A, *et al.* (2020) *Parapseudoflavitalea muciniphila* gen. nov., sp. nov., a member of the family *Chtinophagaceae* isolated from a human peritoneal tumour and reclassification of *Pseudobacter ginsenosidimutans* as *Pseudoflavitalea ginsenosidimutans* comb. nov. *Int J Syst Evol Microbiol* 70:3639-3646

Li Y, Fang W, Xue H, *et al. Brenneria populi* sp. nov., isolated from symptomatic bark of *Populus* x *euramericana* canker. *Int J Syst Evol Microbiol* 2015; 65:432-437.

Li Y, Zheng MH, Wang HM, *et al. Brenneria corticis* sp. nov., isolated from symptomatic bark of *Populus* x *euramericana* canker. *Int J Syst Evol Microbiol* 2019; 69:63-67.

Liu MJ, Jin CZ, Ersiman A, *et al. Flavitalea flava* sp. nov., a bacterium isolated from a soil sample, and emended description of the genus *Flavitalea*. *Antonie Van Leeuwenhoek* 2019; 112:275-281.

Meier-Kolthoff JP and Göker M (2019) TYGS is an automated high-throughput platform for state-of-the-art genome-based taxonomy. *Nat Commun* 10:2182. DOI: 10.1038/s41467-019-10210-3

Meier-Kolthoff JP, Göker M, Spröer C, *et al.* (2013) When should a DDH experiment be mandatory in microbial taxonomy? *Arch Microbiol* 195:413-418 doi:10.1007/s00203-013-0888-4

Meier-Kolthoff JP, Hahnke RL, Petersen J, *et al.* (2014) Complete genome sequence of DSM 30083^T, the type strain (U5/41^T) of *Escherichia coli*, and a proposal for delineating subspecies in microbial taxonomy. *Stand Genomic Sci* 10:2 doi:10.1186/1944-3277-9-2

Meier-Kolthoff JP, Sardà Carbasse J, Peinado-Olarte RL, *et al.* (2022) TYGS and LPSN: a database tandem for fast and reliable genome-based classification and nomenclature of prokaryotes. *Nucleic Acids Res* 50:D801-D807 doi:10.1093/nar/gkab902

Ondov
Oulghazi S, Pedron J, Cigna J, *et al. Dickeya undicola* sp. nov., a novel species for pectinolytic isolates from surface waters in Europe and Asia. *Int J Syst Evol Microbiol* 2019; 69:2440-2444.
Parkinson N, DeVos P, Pirhonen M, *et al. Dickeya aquatica* sp. nov., isolated from waterways. *Int J Syst Evol Microbiol* 2014; 64:2264-2266.
Pattengale ND, Alipour M, Bininda-Emonds ORP, *et al.* (2010) How many bootstrap replicates are necessary? *J Comput Biol* 17:337–354
Pritchard L, Humphris S, Saddler GS, *et al*. Draft genome sequences of 17 Isolates of the plant pathogenic bacterium Dickeya. Genome Announc 2013; 1 (6), e00978-13.
Ramirez-Bahena MH, Peix A, Rivas R, *et al. Bradyrhizobium pachyrhizi* sp. nov. and *Bradyrhizobium jicamae* sp. nov., isolated from effective nodules of *Pachyrhizus erosus*. *Int J Syst Evol Microbiol* 2009; 59:1929-1934.
Richter M and Rosselló-Móra R (2009) Shifting the genomic gold standard for the prokaryotic species definition. *Proc Natl Acad Sci USA* 106:19126–19131
Samson R, Legendre JB, Christen R, *et al*. Transfer of *Pectobacterium chrysanthemi* (Burkholder *et al*. 1953) Brenner *et al*. 1973 and *Brenneria paradisiaca* to the genus *Dickeya* gen. nov. as *Dickeya chrysanthemi* comb. nov. and *Dickeya paradisiaca* comb. nov. and delineation of four novel species, *Dickeya dadantii* sp. nov., *Dickeya dianthicola* sp. nov., *Dickeya dieffenbachiae* sp. nov. and *Dickeya zeae* sp. nov. *Int J Syst Evol Microbiol* 2005; 55:1415-1427.
Sasser M (1997) Identification of bacteria by gas chromatography of cellular fatty acids. MIDI Technical Note 101, Newark, DE: MIDI, Inc.
Snyder LR, Kirkland JJ, Glajch JL. Appendix IV. Preparing buffered mobile phases. In: Snyder LR, Kirkland JJ, Glajch JL. *Practical HPLC Method Development*. Second Edition. John Wiley & Sons, Inc., Hoboken NJ, 1997, pp. 735-739.
Stackebrandt E (2006) Defining taxonomic ranks. In: Dworkin M, Falkow S, Rosenberg E, Schleifer K-H, Stackebrandt E (eds). *The Prokaryotes*. 3rd edition, vol. 1, Symbiotic Associations, Biotechnology, Applied Microbiology. Springer, New York, pp. 29-57
Stackebrandt E and Goebel BM (1994) Taxonomic note: a place for DNA-DNA reassociation and 16S rRNA sequence analysis in the present species definition in bacteriology. *Int J Syst Bacteriol* 44:846–849
Stamatakis A (2014) RAxML version 8: a tool for phylogenetic analysis and post-analysis of large phylogenies. Bioinformatics 30:1312-1313 doi:10.1093/bioinformatics/btu033
Swofford DL (2002) PAUP*: Phylogenetic Analysis Using Parsimony (*and Other Methods), Version 4.0 b10. Sinauer Associates, Sunderland.
Tian Y, Zhao Y, Yuan X, *et al*. (2016) *Dickeya fangzhongdai* sp. nov., a plant-pathogenic bacterium isolated from pear trees (*Pyrus pyrifolia*). *Int J Syst Evol Microbiol* 66:2831-2835
Tindall BJ, Rosselló-Móra R, Busse HJ, *et al.* (2010) Notes on the characterization of prokaryote strains for taxonomic purposes. *Int J Syst Evol Microbiol* 60:249-266
Tóth T, Lakatos T. *Brenneria bubanii* sp. nov., isolated from decaying plant tissues. *Int J Syst Evol Microbiol* 2023; 73: 5854.
van der Wolf JM, Nijhuis EH, Kowalewska MJ, *et al.* (2014) *Dickeya solani* sp. nov., a pectinolytic plant-pathogenic bacterium isolated from potato (*Solanum tuberosum*). *Int J Syst Evol Microbiol* 64:768-774
Volpiano CG, Sant'Anna FH, Ambrosini A, *et al.* (2021) Genomic metrics applied to Rhizobiales (Hyphomicrobiales): Species reclassification, identification of unauthentic genomes and false type strains. *Front Microbiol* 12:614957 doi:10.3389/fmicb.2021.614957
Wang X, He SW, Guo HB, *et al. Dickeya oryzae* sp. nov., isolated from the roots of rice. *Int J Syst Evol Microbiol* 2020;70: 4171-4178.
Wayne LG, Brenner DJ, Coglwell RR, *et al.* (1987) Report of the *Ad Hoc* Committee on Reconciliation of Approaches to Bacterial Systematics. *Int J Syst Bacteriol* 37:463-464
Wick RR, Judd LM, Gorrie CL, *et al.* (2017) Unicycler: Resolving bacterial genome assemblies from short and long sequencing reads. *PLoS Comput Biol* 2017;13(6):e1005595. https://doi.org/10.1371/journal.pcbi.1005595
Yao Y, Sui XH, Zhang XX, *et al. Bradyrhizobium erythrophlei* sp. nov. and *Bradyrhizobium ferriligni* sp. nov., isolated from effective nodules of *Erythrophleum fordii*. *Int J Syst Evol Microbiol* 2015; 65:1831-1837.

Yoon MH and Im WT (2007) *Flavisolibacter ginsengiterrae* gen. nov., sp. nov. and *Flavisolibacter ginsengisoli* sp. nov., isolated from ginseng cultivating soil. *Int J Syst Evol Microbiol* 57:1834-1839
Yoon SH, Ha SM, Lim JM, Kwon SJ, Chun J (2017) A large-scale evaluation of algorithms to calculate average nucleotide identity. *Antonie van Leeuwenhoek* 110:1281–1286
Zhang Z, Schwartz S, Wagner L, Miller W. A greedy algorithm for aligning DNA sequences. *J Comput Biol* 2000;7:203-14.
